# Target-distractor synchrony affects performance in a novel motor task for studying action selection

**DOI:** 10.1371/journal.pone.0176945

**Published:** 2017-05-05

**Authors:** Sebastian James, Olivia A. Bell, Muhammed A. M. Nazli, Rachel E. Pearce, Jonathan Spencer, Katie Tyrrell, Phillip J. Paine, Timothy J. Heaton, Sean Anderson, Mauro Da Lio, Kevin Gurney

**Affiliations:** 1 Adaptive Behaviour Research Group, Department of Psychology, The University of Sheffield, Sheffield, United Kingdom; 2 Insigneo Institute for in-silico Medicine, The University of Sheffield, Sheffield, United Kingdom; 3 Department of Automatic Control Systems Engineering, The University of Sheffield, Sheffield, United Kingdom; 4 Department of Industrial Engineering, Università degli Studi di Trento, Trento, Italy; 5 School of Mathematics and Statistics, The University of Sheffield, Sheffield, United Kingdom; Centre national de la recherche scientifique, FRANCE

## Abstract

The study of action selection in humans can present challenges of task design since our actions are usually defined by many degrees of freedom and therefore occupy a large action-space. While saccadic eye-movement offers a more constrained paradigm for investigating action selection, the study of reach-and-grasp in upper limbs has often been defined by more complex scenarios, not easily interpretable in terms of such selection. Here we present a novel motor behaviour task which addresses this by limiting the action space to a single degree of freedom in which subjects have to track (using a stylus) a vertical coloured target line displayed on a tablet computer, whilst ignoring a similarly oriented distractor line in a different colour. We ran this task with 55 subjects and showed that, in agreement with previous studies, the presence of the distractor generally increases the movement latency and directional error rate. Further, we used two distractor conditions according to whether the distractor’s location changes asynchronously or synchronously with the location of the target. We found that the asynchronous distractor yielded poorer performance than its synchronous counterpart, with significantly higher movement latencies and higher error rates. We interpret these results in an action selection framework with two actions (move left or right) and competing ‘action requests’ offered by the target and distractor. As such, the results provide insights into action selection performance in humans and supply data for directly constraining future computational models therein.

## Introduction

Selecting an appropriate target for action in an unpredictable environment is crucial to survival for all animals [1, 2]. As an example, consider the choice between grasping for a nutritious blue fruit whilst ignoring the distraction of poisonous red berries. Such an interaction is dependent upon an integration of perception and action [3]. The simple task of perceiving the target berry and grasping it is typically coordinated by a rapid eye movement toward the target, followed by goal-directed hand and arm movements [4–6]. Individuals must have the ability to rapidly distinguish between relevant and irrelevant information within the environment, and selectively initiate the most suitable movement; a process referred to as *action selection*; a term which began to be used within the context of biological motor control in the late 1980s [7–9].

The ability to select the appropriate movement requires the individual to choose between an abundance of conflicts within competing brain systems [10]. In our example, the blue berries must be selected as the grasp target in preference to the red berries, but it may also be necessary for the animal to select the action of fleeing if it detects the approach of a predator or returning to the safety of its nest if it is sated or tired. Action selection may refer either to the within-goal selection of a movement (towards the blue berry or towards the red berry) or to a selection between different goals (continue feeding or return to nest).

In this paper, we are concerned with the former, within-goal selection choice. We wanted to investigate motor control and action selection, and to this end we developed a task in which we could measure reaction times, error rates and trajectories of stylus movements towards a target line in the presence of a differently coloured distractor line. Here, we present the task, which we call the *line task*, along with a sample implementation of the task with a healthy cohort of subjects.

For many years, the saccadic system has been used for the investigation of action selection and decision making [11–16]. A well structured set of experimental protocols has been developed by researchers in this field. Pro-saccades, for which the subject must move their eyes towards a target, are elicited in ‘gap, step and overlap’ paradigms [17]. The anti-saccade, in which the subject is required to saccade *away* from a target, has a proposed protocol [18]. Saccades provide a good window through which to view sensorimotor decisions because they can be considered to have only two degrees of freedom and the anatomy is reasonably well understood [19–21]. The predictive properties of the superior colliculus [22], frontal eye field [23] and lateral intraparietal area [14, 24] have all been investigated. However, the saccadic system is highly specialised for eye movements and an understanding of it may not generalise to motor control of limb movements. Furthermore, saccades and reaching decisions are closely connected [25]. Although an observation of a primate making a reaching movement will show that the saccade precedes the reach (as described in the berries example), internal neural control processes for the two movements are not necessarily sequential [26] and the reaching movement has been shown to be specified prior to the onset of the saccade [25]. Reaching movements affect both saccade trajectories [27] and latencies [5, 28, 29] and they may be planned in an eye-centered coordinate system [27, 30]. Because of this close relationship, it is important to conduct behavioural studies using both reach and saccadic eye movements to reveal internal, decision making processes.

The simplicity of saccadic paradigms is usually lost in experiments on limb control. Thus, many reach to grasp experiments involve the recognition of targets and distractors within a three dimensional space. Consequently, higher-order visual processes such as depth perception are employed [31]. Additionally, tasks requiring prehension movements involve the recognition of object size, orientation and shape [32]. Both the ventral and dorsal streams are therefore likely to be engaged whilst completing reach and grasp tasks [33]. As well as high order visual processing, these tasks require intricate grasp movements and complex motion throughout a three dimensional space [3, 34–37]. A consequence of the complexity associated with these tasks is that participants may be selecting from not a few, but from many competing actions or affordances [1].

There does exist a class of experiments known as *step-tracking tasks* which study reach movements in response to simple, quick movements of a target. For example, in one early, electronic trajectory-tracking apparatus, Trumbo and colleagues displayed a vertical hairline target on the 5 inch screen of an oscilloscope [38]. Subjects were required to match the location of a second line to the target by controlling the position of their arm which was fixed but allowed to pivot at the elbow [39]. Hallett and co-workers used a similar apparatus to that used by Trumbo et al. in an investigation of EMG activity in biceps and triceps muscles [40]. Other researchers performed step-tracking experiments for wrist movements, tracking a target moving in one dimension [41, 42] or two [43]. In each of these step-tracking experiments the subject would move a manipulandum lever to position a cursor image so that it matched the location of a target image. This task forms a simplified framework by which task selection, as expressed in limb movements and distinct from (or indeed, together with [39]) saccadic eye movement, can be studied. However, dissociates the end-points of the saccade and limb movements in a way which is not typical of natural behaviour; in the example, the target of the animal’s saccade to the blue berry matches the target of the reach to grasp the berry. Studies which investigate the positional reference frames employed by the brain for saccadic and reach movements may not be expected to observe the same results for the arm movements of the step-tracking tasks as for natural reach-to-grasp movements.

Experiments to investigate the nature of arm movements are typically laboratory based. Like the step-tracking experiments described above, they usually consist of a screen to display the stimuli and an input system which the subject uses to interact with the display. The input may be via a set of buttons [44, 45], a joystick [2] or a computer keyboard [46]. Movement trajectories are collected using specialised equipment [47, 48] or may only be inferred from information about the trajectory end point [2].

Here, addressing the issues of complexity, lab-bound constraint and target/effector end-point dissociation, we present the *line task*; a novel, inexpensive and easy to use experimental apparatus for the study of one dimensional target selection and reach. The task is designed to minimise the cognitive complexity of the target selection and reach-tasks which it is intended to probe. Thus, it uses vertical lines which move in one dimension only (horizontally), meaning that subjects could plan their movements internally in a one dimensional space, even though the end point moves in two dimensions and the arm movement is carried out in multiple rotational dimensions. Colour is used to allow subjects to distinguish between target and distractor stimuli. No other object features need to be recognised by the subject’s visual processing systems. The apparatus provides the ability to record reaction times and detailed trajectory information. Because it is implemented on standard tablet computer hardware, the line task is not constrained to the laboratory and can be used by individuals in their own homes to generate movement data which can then be collected via the internet for analysis by the researcher.

The device was tested and evaluated on an action selection task (with synchronous and asynchronous distractor) on a large sample size of 55 test subjects, to verify that the device met the design requirements. The task was chosen to demonstrate the particular utility of the device design; tracking a target line in the presence of a distractor line. This task revealed for the first time a particular deficit in action selection with an asynchronous distractor: increased latency compared to a synchronous distractor. This new result is distinct from previous action selection investigations because the results do not have potential confounding factors associated with higher level cognitive loading and explicit 3D visual/movement transformations, illustrating the value of the line task.

## Materials and methods

### Line task

**Fig 1:**
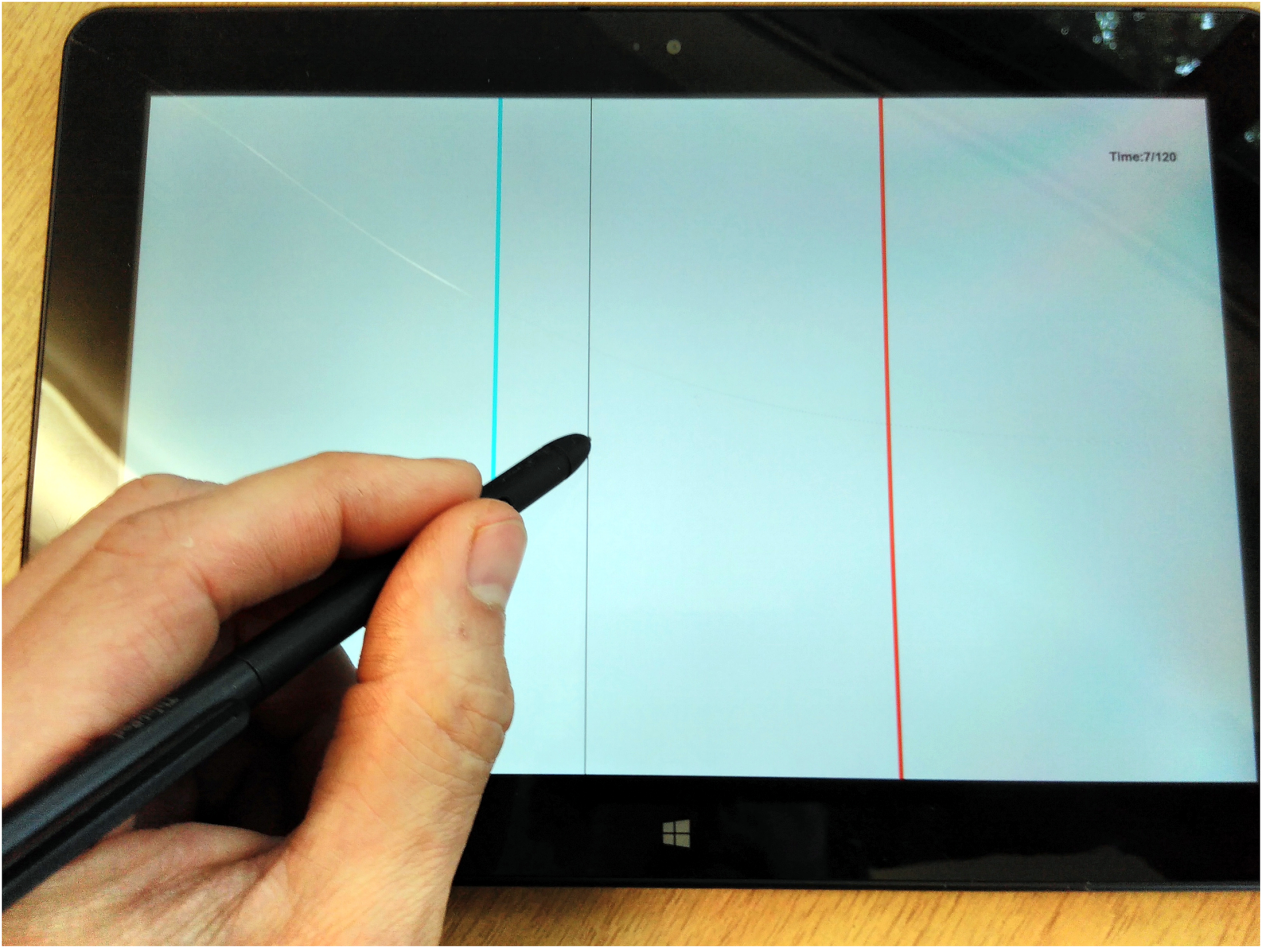
The line task in operation. The cyan target line is visible, along with a red distractor and the narrower, black stylus line.

The line task was presented on a tablet PC (see Fig 1 and [49]). It requires participants to move a stylus to the location of a vertical, cyan line (the target) whose position changes randomly, possibly in the presence of a red line (the distractor), whose position also changes. There were three experimental conditions for the line task. One in which there was ‘No Distractor’ (ND), one in which the distractor changed position asynchronously with respect to the target line (‘Asynchronous Distractor’ or AD), and one in which the distractor line changed location whenever the target’s position changed (‘Synchronous Distractor’ or SD). In each condition, the target line would spend a randomized period of time *T*_*t*_ at each location before simulaneously disappearing from the old location and appearing at the new location. *T*_*t*_ is drawn from a normal distribution with mean 1.6 s and variance 0.636:

$$T_{t}∼N\left(\mu =1.6,\sigma^{2}=0.636\right)\;\text{seconds}$$ (1)

In the synchronous distractor condition, the time between distractor location changes is identical to *T*_*t*_. The new target location, *x*, was computed by first generating a random location offset, *δx*, then adding this to the current location, subject to the bounds of the edge of the screen:

$$\begin{array}{c} \delta x∼\frac{w}{4}\;U\left(-1,1\right) \end{array}$$ (2)

$$\begin{array}{c} x=\left\{\begin{array}{cc} x^{\prime }+\delta x & \text{for}\;\frac{w}{10}<x^{\prime }+\delta x<\frac{9w}{10}\\ x^{\prime }-\delta x & \text{otherwise} \end{array}\right. \end{array}$$ (3)

where *x*′ is the previous location of the target, *w* is the width of the screen in pixels and *U*(−1,1) is a uniformly distributed random 32 bit floating point number between -1 and 1. In the asynchronous distractor condition, the time between target location changes is the same as in the ND and SD condition with *T*_*t*_ ∼ *N*(*μ* = 1.6, *σ*^2^ = 0.636) s. The time between distractor location changes was given by:

$$T_{d}∼N\left(\mu =0.8,\sigma^{2}=0.158\right)\;\text{seconds}$$ (4)

Note the shorter periods; the asynchronous distractor changes location more quickly than the target. The locations of the asynchronous distractor are calculated according to:

$$\delta x∼\frac{w}{2}\;U\left(-1,1\right)$$ (5)

$$\begin{array}{c} x=\left\{\begin{array}{cc} x^{\prime }+\delta x & \text{for}\;\frac{w}{10}<x^{\prime }+\delta x<\frac{9w}{10}\\ x^{\prime }-\delta x & \text{otherwise} \end{array}\right. \end{array}$$ (6)

which differs from the computation of the target locations only in that the distractor location is permitted to change by up to half the screen width, which allows the distractor to appear either side of the next target location, even if that is at the most distal location possible. In the synchronous distractor condition, the distractor line changes location simultaneously with the target line, and by an equal and opposite distance. If the distractor line would need to be displayed beyond the edge of the screen, then it was placed *w*/20 pixels from the edge.

### Experimental design

The line task experiment had a one way, repeated measures design. The dependent variables were the movement latency and the error rate. Movement latency refers to the time taken to initiate a movement after target stimulus onset. Significant movement away from the target counted as an error. The definitions of error and latency are given in *Latency extraction*, below. The related samples independent variable was the distractor condition, with three levels (synchronous, asynchronous and no-distractor). A linear mixed effects model was fitted with distractor condition as the fixed effect and individual included as the random effects component. An alpha value of 0.05 was selected for significance testing.

### Apparatus and stimuli

The line task was presented on a Lenovo ThinkPad 10 (20C1) tablet computer with a 218 mm x 137 mm (1920 pixel x 1200 pixel) colour display. The tablet was positioned in landscape orientation and thus the height was 137 mm and the width was 218 mm (see Fig 1). A vertical cyan (RGB24 representation: #fcfc00) target line was displayed on the white (#fcfcfc) background of the display, along with a vertical black (#000000) line indicating the current horizontal position of the participant’s stylus (the vertical location of the stylus was not recorded or displayed). In some cases, a third, vertical red (#fc0000) distractor line was also displayed. All lines extended from the top of the display to the bottom; the full height of the display. Target and distractor lines were 8 pixels wide; the black line indicating current position was 2 pixels wide and overlaid the target or distractor lines when co-located with either of them. A stylus (ThinkPad Tablet Digitizer Pen, manufacturer: Wacom) was used to track the target line. Use of this particular stylus disables the capacitive touch screen on the ThinkPad, preventing contact with the heel of the hand from registering as an erroneous stylus position. The position of the stylus was digitised approximately every 5 ms. A software timer guaranteed that the stylus position was recorded at least every 5 ms, though if the system was busy, a longer gap could occur between recordings. For each datum, the time was recorded with a precision of 1 ms. The refresh rate of the tablet display screen was 60 s^−1^. The line task was programmed in C++ using the Qt framework version 5.3.

### Participants

Overall, 61 psychology undergraduate students participated in the study. 6 participants were removed prior to analysis due to errors in the implementation of the tasks (the target’s mean jump time had been set to values other than 1.2 s for one or more conditions). This left 55 participants (9 males and 46 females). 47 of these participants were right handed; 8 were left handed. All participants had normal to corrected vision. The chronological age of the 55 participants ranged from 18 to 28 years old with a mean age of 19.2 years (SD = 2.1).

The majority of participants were an opportunity sample. These participants were recruited via the University of Sheffield’s online research participation system, in order to gain course credit. The experiment was conducted by five different experimenters, who were all involved in the recruitment and data collection. The study received ethical approval from the Psychology department’s ethics sub-committee. Written informed consent was also collected from all participants.

### Procedure

Each participant completed the line tracking tasks individually within the same dimly lit cubicle room. On arrival, the participants were asked to sign a consent form which outlined the experimental aim. They were then given verbal instructions to follow the blue target line with the stylus pen in all trials, while ignoring other stimuli. Participants were explicitly told that they must complete the tasks “as quickly and as accurately as possible”, implicitly allowing each participant a decision between movement speed and accuracy. The tablet was placed flat on the table, directly in front of the participant. The tablet’s display brightness setting was set to automatically adjust based on the surrounding light level. To start the tasks, the participants were verbally instructed to press the play button with the stylus pen when they were ready. Participants then completed a 30 second practice task in which the mean jump time was set to 3 seconds (thus slower than the main tasks during which data was recorded). After the practice task, participants had a chance to ask any further questions about the tasks, after which they proceeded to complete the three task conditions (ND, SD and AD), each of which lasted for 120 seconds. Before the participant started the tasks, the experimenter would leave the room and dim the lights. The sequence in which the task conditions were presented was determined randomly for each participant. When participants had completed the first task, they informed the experimenter. The end of a task was indicated by a return to the line task application’s start-up screen. The experimenter would then enter the room and adjust the settings on the tablet for the next task. The participant would then proceed to complete the next 120 second task condition. This process was repeated until the participant had completed all three task conditions.

### Latency extraction

The data collected, and all of the scripts used to analyse them are available at https://github.com/ABRG-models/linetask2014.

The raw data generated by the line task application consisted of position data for the target line, the distractor line (if any) and the stylus position, sampled at approximately 5 ms intervals. For each subject, one raw data output file was produced for each of the experimental conditions. These raw data files were analysed by a script (**lt_analyse.m**) implemented in Octave (version 3.8.2), which algorithmically recognises the beginning and end of a stylus movement, returning latency to first movement for each trial, along with movement error information. The volume of data necessitated an automated approach; there were a total of 17617 individual target and distractor events to analyse. In order to verify that the latency extraction script was producing reliable results, an alternative method based on resampling and movement filtering was also applied to the ND and SD cases, although this method was not extended to handle the many subtleties of the more complex AD condition.

#### Primary latency extraction method

The first latency extraction method was based on the raw stylus position information and its first derivative.

**Fig 2:**
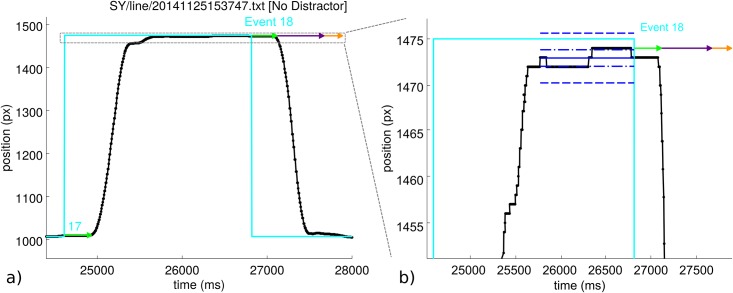
**a)** An example event—number 18 from this data set. The horizontal position of the vertical target and stylus lines is plotted against time. The black line shows the stylus position. The cyan line shows the position of the target. At event 18, the position of the target shifts from 1475 px to 1007 px. Event 17 is also visible. The grey dashed box indicates the region of the graph which is expanded in plot b). **b)** The expanded region from plot a) which shows the result of the movement onset analysis in more detail. Blue lines indicate the ‘stable stylus position’ period which precedes the target event. Blue dot-dash lines indicate 1 standard deviation in position over this period; blue dashed lines indicate 3 standard deviations. The latency to first movement is shown as a green arrow, with a purple arrow showing the algorithm’s estimate of the length of the first, uninterrupted smooth motion and the orange arrow indicating the detected time for the motion to complete. Here, the subject moved in the correct direction.

The script begins by identifying the times of target and distractor events. A target/distractor event is the event that the target/distractor changes position on the screen. To determine the latency to movement for a given target event it is necessary to find the time at which the stylus first begins to move. The script computes the average position of the stylus prior to the event; the ‘stable stylus position’. The mean and standard deviation of the stylus position data from the time of the end of the previous movement to the time of the current target event is calculated. Motion is detected if, post-event, the stylus position departs further than 3 pre-event standard deviations from the pre-event mean position and has a speed greater than 0.05 pixels per millisecond (50 px/s; 5.7 mm/s, cf. [50]). The displacement at this time step gives the initial stylus direction. To determine as closely as possible the start of the movement, the algorithm steps back along the trajectory until the stylus position is within 1 pre-event standard deviation from the pre-event mean position (dash-dot blue lines in Fig 2). This gives the movement latency, shown in the figures as a green arrow.

After a target event, if the motion was initially in the direction of the target line’s new position, then this was recorded as a correct movement. If the motion was in the opposite direction, this was recorded as a movement error. Feedback on whether or not the movement was in error was *not* given to the subject during the task. Note that a very brief movement in the wrong direction was counted as an error; in most cases subjects correct their movements quickly (in around 100 ms). A motion towards the distractor line following a distractor event was also recorded as a movement error. In the asynchronous condition, a distinction was made in the analysis between target and distractor movement errors.

It was not possible to measure a latency for every event; some were omitted from this analysis. Most of these events were also omitted from error analysis. A list of the reasons for omitting events is given in S1 Appendix.

The analysis of events was significantly more complex in the asynchronous distractor condition. In both the no-distractor condition and the synchronous distractor condition, each event was well separated from preceding and subsequent events. In most cases, as long as a stable stylus position was achieved before the event, then the latency and the movement direction error could be easily determined, as in Fig 2. The difficulty in the asynchronous distractor condition was that distractor events could occur at any time, and had been programmed to occur in larger numbers, so that instead of analysing 50 to 60 target events (or target+distractor combined events for the synchronous condition) it was necessary to analyse roughly 50 to 60 target events and also over 100 distractor events for each trial.

#### Movement errors

**Fig 3:**
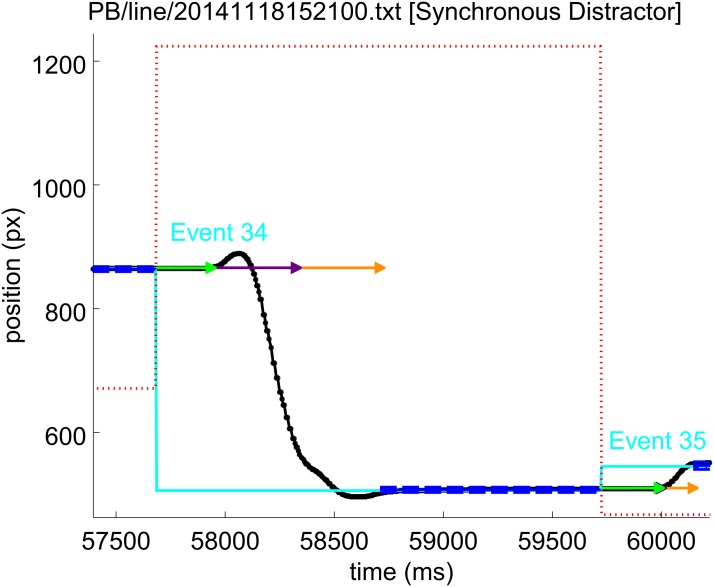
An example movement error event. The line colours have the meanings given in Fig 2. The red dotted line is the horizontal position of the vertical red distractor line. Following event 34, this subject moved the stylus towards the distractor (red dotted line). Within 100 ms of the movement, the subject corrected the direction and moved to the target position.

In the statistical analysis for the no distractor condition, movement errors were always associated with target events. In the synchronous condition, because a distractor event always occurred simultaneously with the target event, movement errors could have been associated with either event; the target event was chosen to match the no-distractor condition. For the asynchronous distractor condition, movement errors which correlate with target events were distinguished from those which followed distractor events. For a target event, a trajectory which was initially directed *away* from the target was recorded as a movement error. For a distractor event, a movement *towards* the distractor was considered to be a movement error. Fig 3 shows an example movement error event.

#### Alternative latency extraction method

To verify the accuracy of the latency extraction described above, an alternative approach was developed, and the results compared for the no-distractor and synchronous distractor conditions.

This method relies on the computation of the sign of the stylus velocity. Because the touch position is quantized, the stylus velocity is exactly zero as long as the readout position is the same pixel. If the stylus drifts slowly then a series of short pulses are observed in the velocity. Voluntary movements are sustained for some time and hence, the velocity maintains the same sign for the same interval.

**Fig 4:**
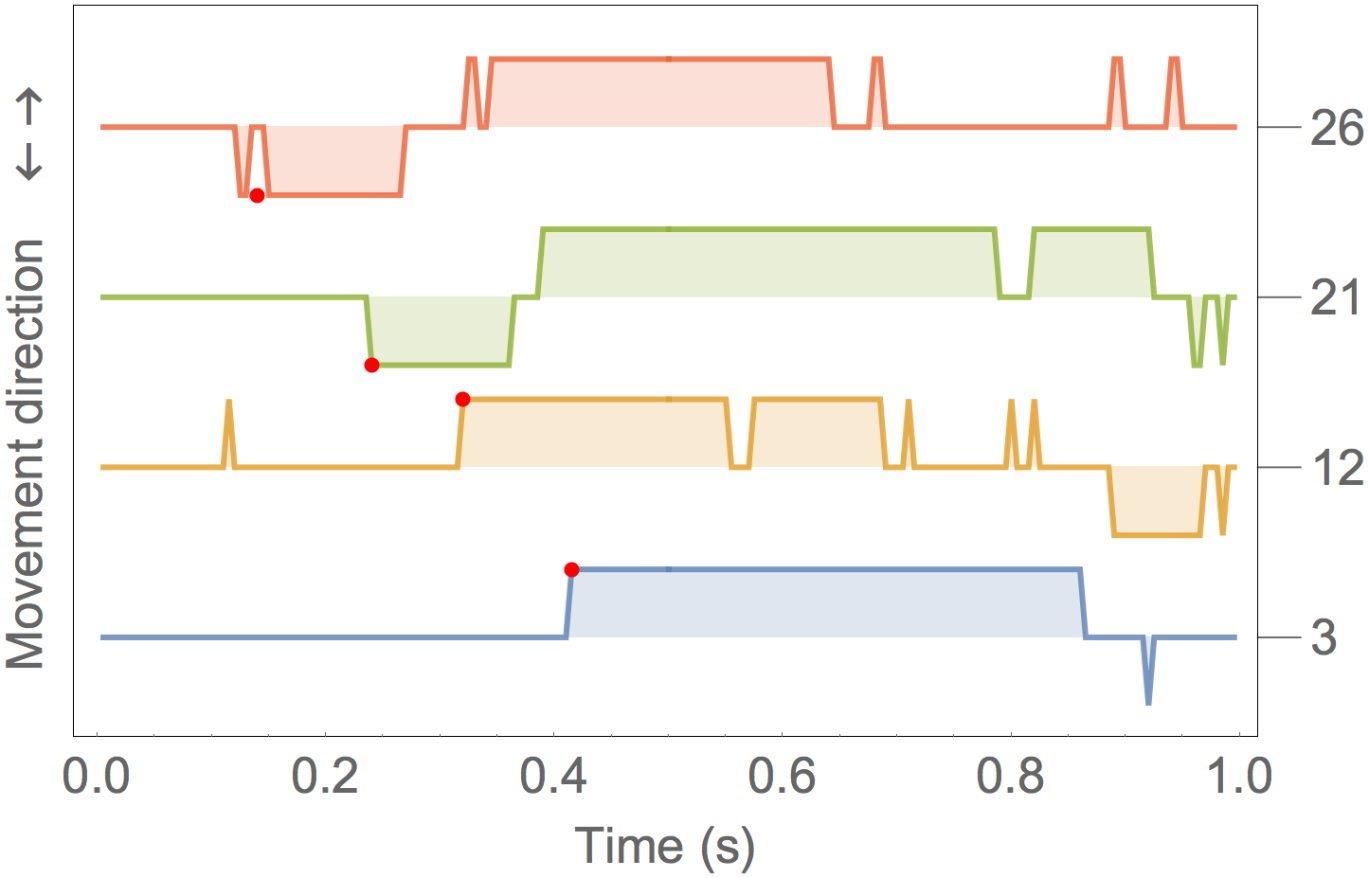
The sign of the stylus velocity plotted for 4 example events, numbered 3, 12, 21 and 26 on the right hand axis. Each event is aligned with the target position change at 0.

Fig 4 gives an example. In the event 3, the stylus remained stationary over the same pixel until time t = 0.4 s. Then the sign of the velocity remains positive (towards the target) until t = 0.85 s. This is an intentional target reach movement. Note that at about t = 0.9 s there is a negative velocity spike which represents the stylus stepping back one pixel. Event 12 is more complex; in this case at about t = 0.1 s there is a spike because the stylus steps to the next pixel. If spikes are ignored, the real movement is made of three steps two initial steps towards the target and one backwards (because the target was overshot). Example 21 represents an error: at t = 0.25 s the stylus moves away from target, but then, at t = 0.38 s the direction of movement is correct towards the target. Example 26 is another error example: in this case the movement starts slowly in the wrong direction and looks like a spike preceding the sustained phase.

In order to detect the time when an intentional movement begins, spikes needs to be ignored. This has been done by filtering the velocity sign signal with a median filter (the radius of the median filter determines the duration of the neglected spikes and was 40 ms in our implementation, which also means that only movements lasting longer are considered intentional). The red dots in Fig 4 represent the detected movement onset. The sign of the velocity at that point indicates whether the movement is correct or an error.

The method may produce outliers of two kinds: a) if there is no movement, no delay is computed (a condition that had instead to be explicitly omitted above), b) if the subject is distracted and reacts too late, a long reaction time is produced which is filtered as an outlier.

This method has been used with only two conditions: a) target jump size greater than 20 pixels (as above) and b) drift of stylus smaller or equal to 1 pixel in the first 0.15 s. These two conditions are intentionally different from those listed above so that we can compare the results of a different method with slightly different omission criteria.

This second method has confirmed the estimation carried out with the primary method: the mean reaction time with this method is systematically slightly shorter than the primary method due to differing criteria for event omission. Table 2 includes the results for the alternative method alongside those for the primary method.

### Statistical analysis

The statistical analyses reported here were carried out in R [51] and python.

**Fig 5:**
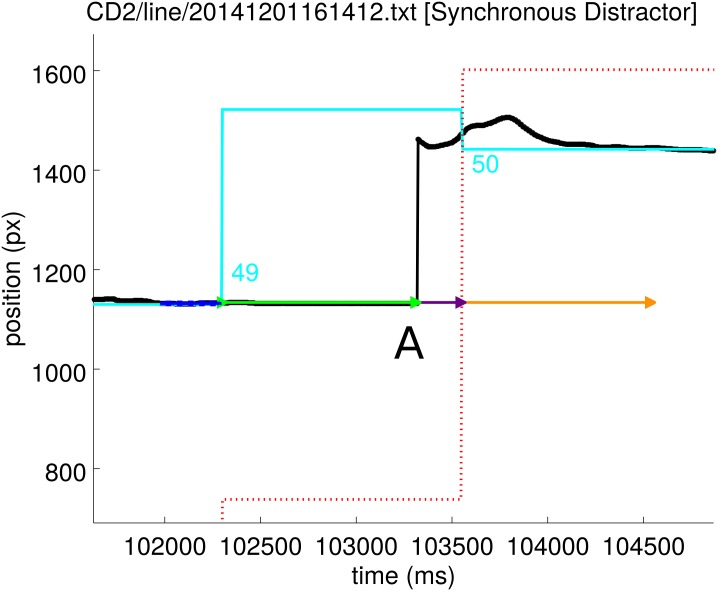
An example event during which the stylus (black line) lifted and it seems that movement did not occur until 1013 ms after the target event (label ‘A’) when the stylus made contact with the screen and data could again be recorded. This latency was recorded by the latency extraction script, but was then excluded by the median absolute deviation method employed to exclude outliers. The cyan line is the target position, the red dotted line is the distractor position. For the meaning of other coloured lines, refer to Figs 2 and 3.

Prior to applying analyses, outlier data were excluded from the latency measurements for each individual. For details, code and data, see S2 Appendix and https://github.com/ABRG-models/linetask2014. Causes of outlier data included an individual becoming distracted from the task and missing one or more targets or the stylus lifting from the screen and the position failing to update, which occurred for the example shown in Fig 5. Outliers were excluded using a “median absolute deviation from the median” method [52] with the modified Z-score threshold set to 3.5. After excluding outliers, the mean latency in each condition was computed for each individual.

## Results

Other than where indictated, the results presented here have been derived from analysis of latencies and error rates produced by the primary latency extraction method.

### Latency extraction

The mean number of target events in the tasks was 64.7 with standard deviation (SD) of 6.9. Of these, the Octave latency extraction script was able to measure latency to first movement in 53.1 (SD = 7.8) cases. The script was most successful in collecting latencies from the no-distractor task condition, returning a latency for 89% of trials across individuals (range 75% to 100%). The presence of a distractor line increased the variability of the stylus trajectory, reducing the number of target events for which a latency could be determined. In the synchronous distractor condition, latencies were returned from 79.1% of trials (range 58% to 96%); in the asynchronous condition from 78% of trials (range 57% to 100%).

### Analysis of latencies

**Fig 6:**
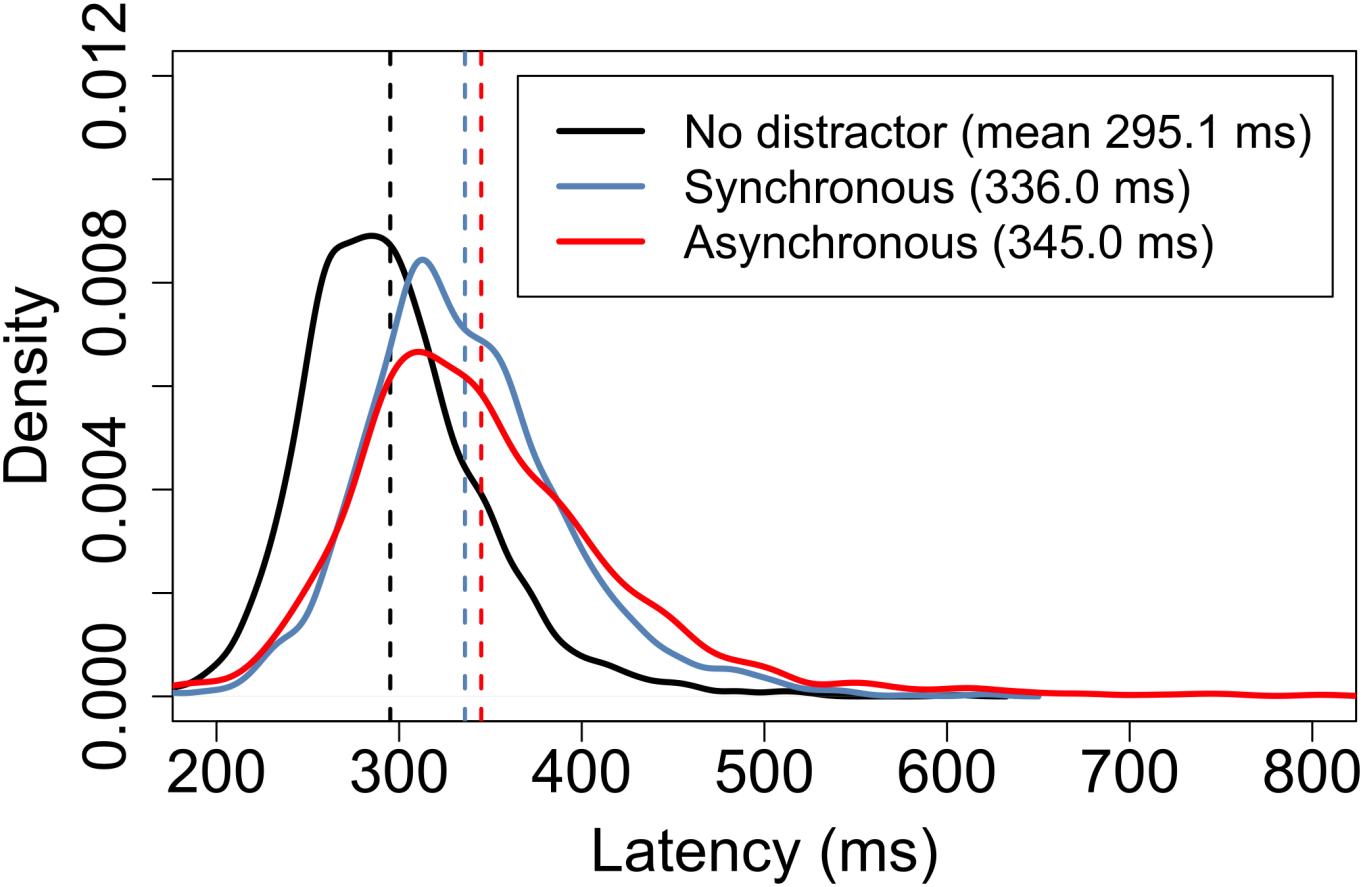
Probability density function approximations for 2013 latency measurements for each of three experimental conditions. The graph indicates the wide variances in movement onset latencies which are typical in any animal behavioural experiment. This graph shows latencies for correct movements only. Black indicates data for the no distractor condition, blue for the synchronous distractor condition and red for the asynchronous distractor. Dashed lines indicate the means. Although the synchronous and asynchronous curves appear very similar, bootstrap resampling of the two samples indicates that there is a very low probability that they are generated by the same population distribution.

Fig 6 shows probability density functions for a subset of the measurements taken from all 55 subjects in the three conditions. Here, the maximum possible equal number of correct-movement latency measurements from each condition were randomly sampled for each subject, giving datasets of length 2013 for each condition.

**Fig 7:**
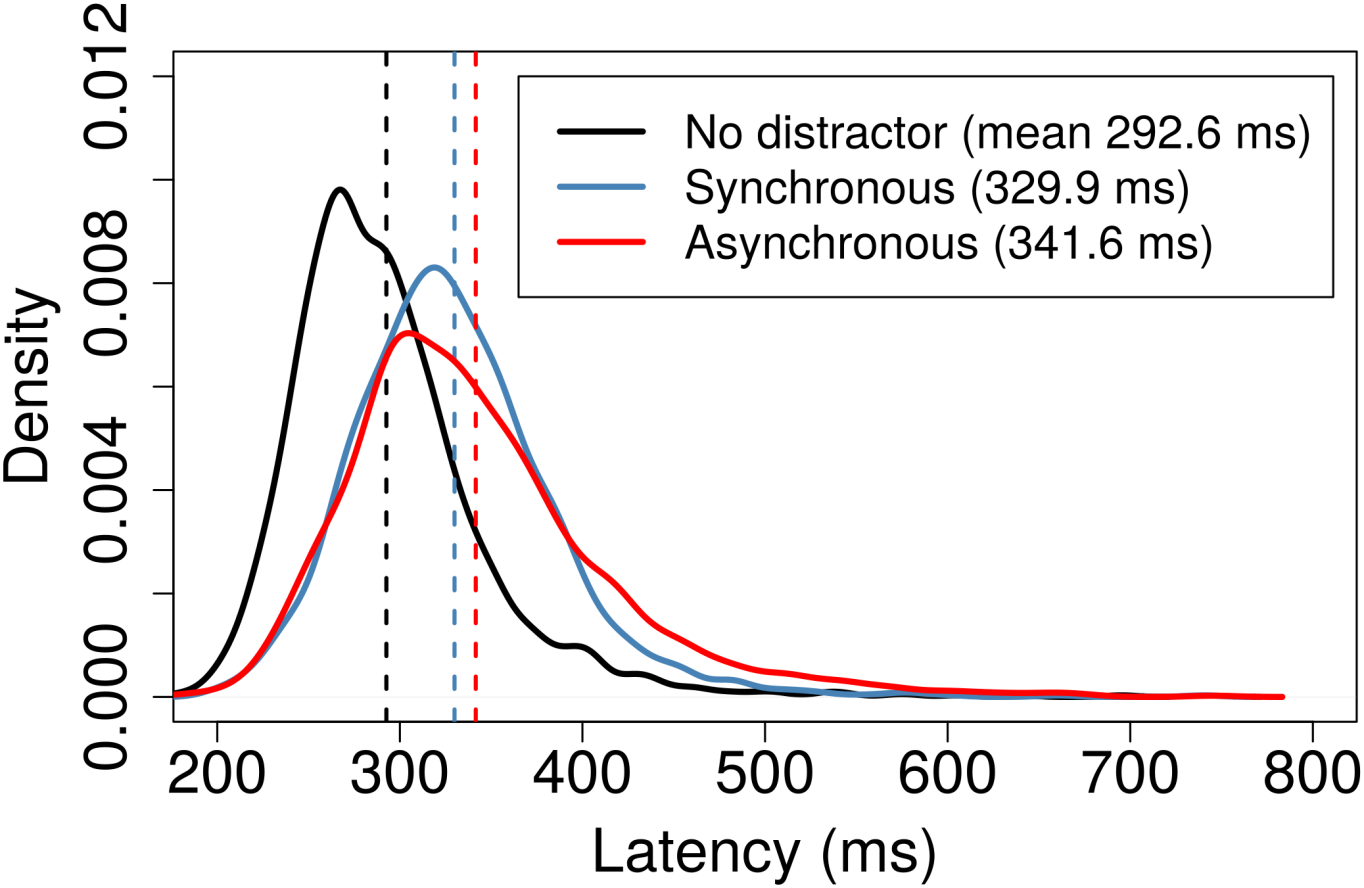
Probability density function approximations for latency measurements determined using the alternative method. The presentation matches Fig 6: Black indicates data for the no distractor condition, blue for the synchronous distractor condition and red for the asynchronous distractor. Dashed lines indicate the means.

Fig 7 shows the result of the same analysis of the latencies determined by the alternative latency extraction method. Inspection of Fig 7 shows that the latency distributions for the alternative extraction method preserve the form of those shown in Fig 6 for the primary method. The sample means and standard deviations are summarised in Table 1. To estimate the standard error of the sample mean, we created bootstrap samples of 2013 observations drawn with replacement and then computed the mean for each of 4×10^6^ replications.

**Table 1:** Summary of latency means with standard deviations, for the primary and alternative latency extraction methods. A bootstrap estimate of the standard error of the mean is also given.

Condition	Mean (ms)	Std. Dev.	Std. Err.
	Primary latency extraction method		
ND	295.1	51	1.1
SD	336.0	57	1.3
AD	345.0	79	1.8
	Alternative latency extraction method		
ND	292.6	53	1.3
SD	329.9	56	1.5
AD	341.6	71	2.0

We constructed a linear mixed effects model on the latencies to determine whether there was a statistically significant difference in latency to first movement predicted by the fixed factor of the three conditions ‘No Distractor’, ‘Synchronous Distractor’ and ‘Asynchronous Distractor’. The individual subject was used as the random factor to give an model of the form (using R-like notation):

latency∼condition+(1|subject)+ϵ (7)

**Fig 8:**
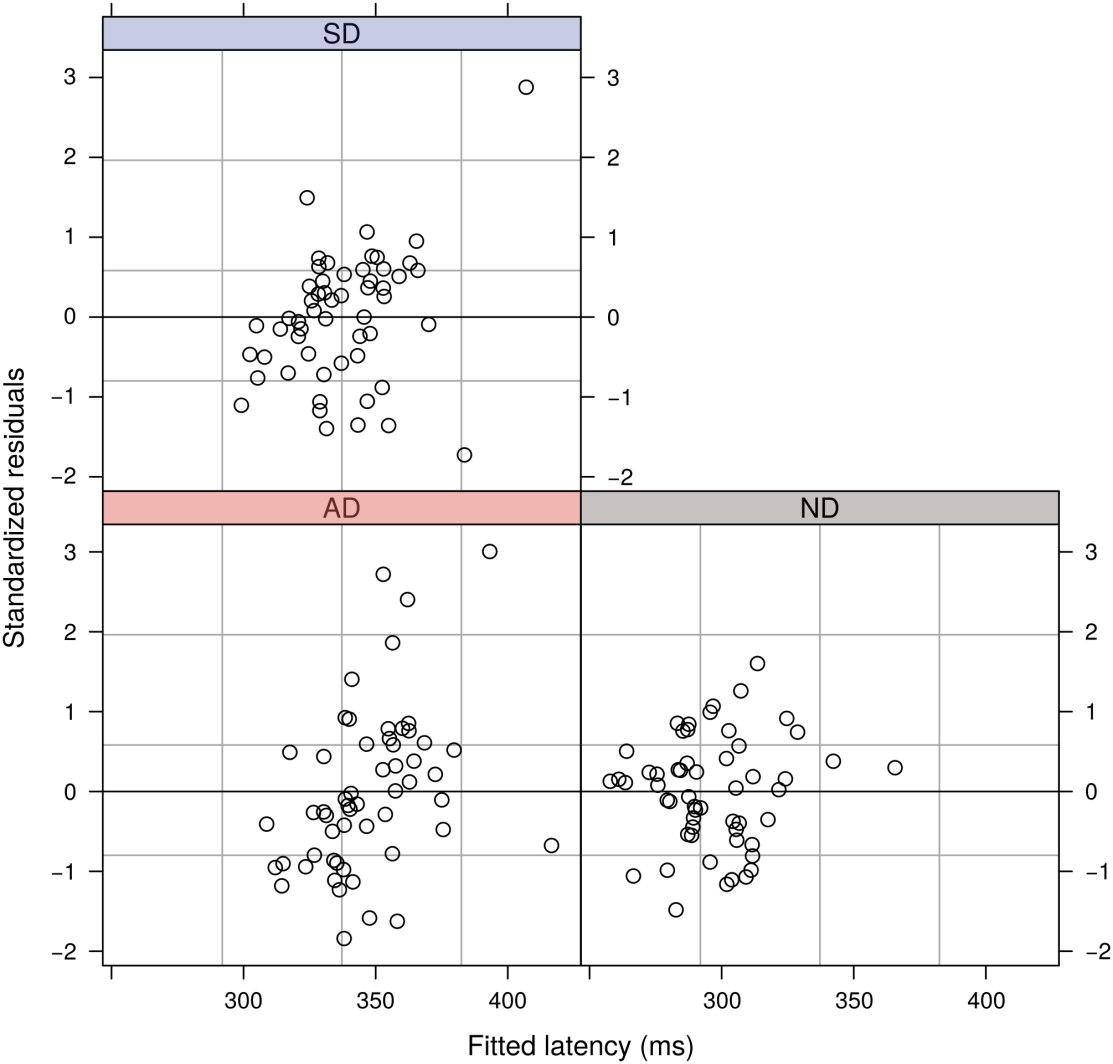
Standardized residuals indicating the proximity of each individual subject’s mean latency in the three conditions from the fit of the linear mixed effects model.

The model showed that the different task conditions elicited statistically significant changes in latency to first movement (*χ*^2^(2) = 130.53, p<2.2×10^−16^). Plots of the residuals from the model (Fig 8) do not suggest any evidence for heteroscedasticity or non-normality.

To determine differences and confidence intervals between pairs of conditions we applied a bootstrap analysis to the data. We computed an estimate of the standard error of the difference between the means in the three conditions. These results are summarised in Table 2. The Z-scores indicate that the effect size is large for either of the distractor conditions compared with the no distractor condition, but small for the SD/AD pair.

**Table 2:** Summary of bootstrapped two-sample analyses of latency values. The difference between the sample means is shown (e.g. for ND/SD, the SD condition has a latency which is 40.9 ms longer than for ND) along with the computed estimate of the standard error of the difference and the corresponding Z-score. The last column shows the result of the Studentized t-test to determine the probability that the two samples are drawn from the same population. For comparison, the corresponding results for the alternative latency extraction method are shown here.

Conditions	Difference (ms)	SE	Z-score	t-test p-value
	Primary latency extraction method			
ND/SD	40.9	1.67	24.5	<2.4×10^−7^
ND/AD	49.8	2.07	24.1	<2.4×10^−7^
SD/AD	8.92	2.24	3.98	0.000015
	Alternative latency extraction method			
ND/SD	37.3	2.00	24.5	<1×10^−3^
ND/AD	49.0	2.34	20.9	<1×10^−3^
SD/AD	11.6	2.45	4.73	<1×10^−3^

**Fig 9:**
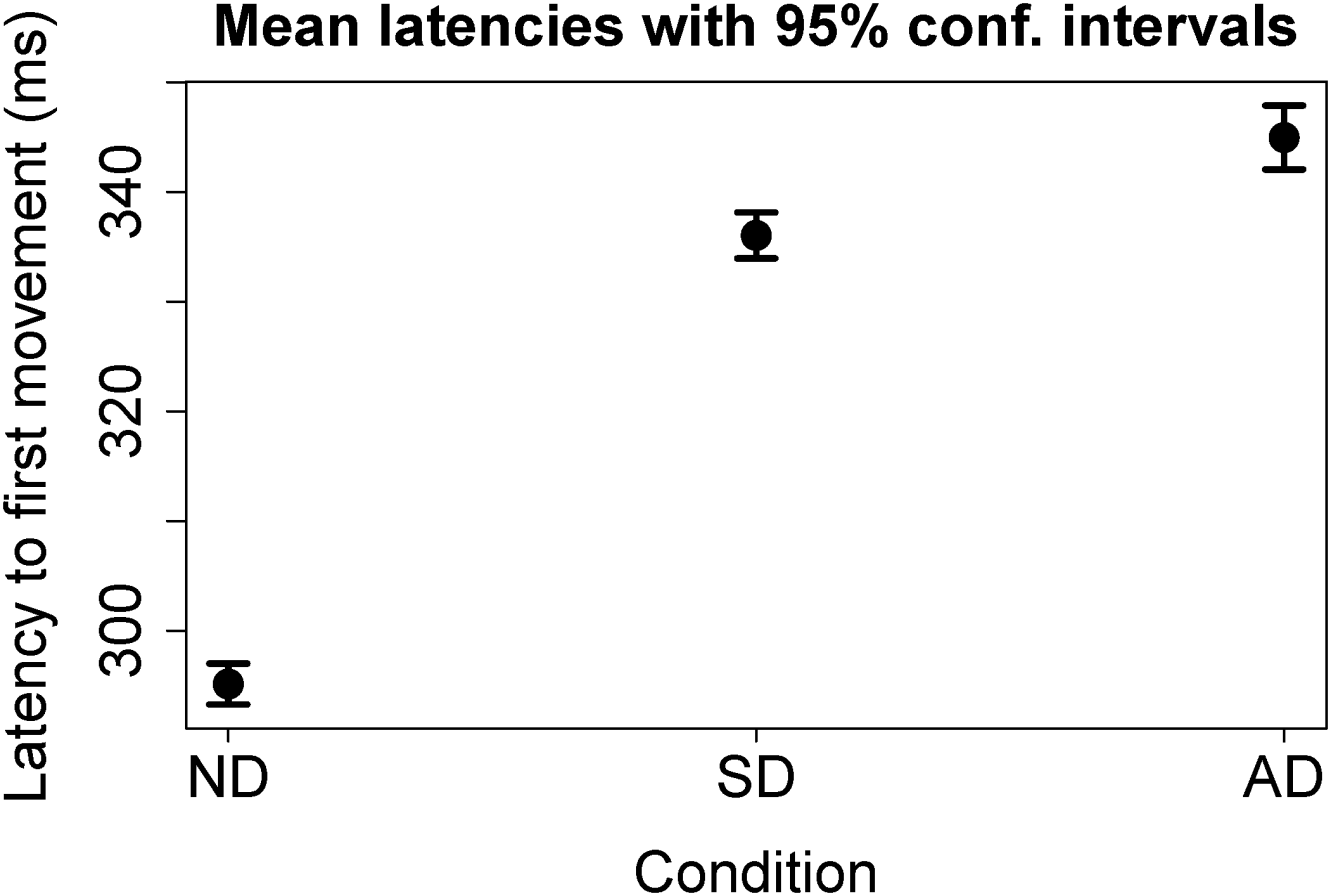
Mean latencies to first movement in the three experimental conditions ‘No Distractor’ (ND), ‘Synchronous Distractor’ (SD) and ‘Asynchronous Distractor’ (AD) as determined using the primary latency extraction method. In this plot, the error bars show 95% confidence intervals, determined by a bootstrap analysis of the sample means.

To obtain a better estimate of the probability that the distributions were drawn from the same population, especially as the apparent effect size between the SD and AD conditions is small, we applied a bootstrapped, Studentized t-test. This test rejected the null hypothesis that distributions were drawn from the same population in each case: For ND compared with either AD or SD: p<2.5×10^−7^; for AD compared with SD: p = 0.000015 (4×10^6^ resamples). The mean latencies, with 95% confidence intervals computed from a bootstrap analysis of the sample means, are shown in Fig 9.

We analysed the latencies for error movements in each condition to compare these with latencies for correct movements. In each condition, the mean latency for error movements was significantly shorter than for correct movements (p<0.00002 SD; p = 0.0001 AD; p = 0.005 ND) by about 100 ms. Refer to Table 3 for the results.

**Table 3:** Mean latencies for movements directed towards the target (non-error) and for movements which were in error and directed away from the target. Standard error and the probability of the two samples being drawn from the same population were calculated using the bootstrap method described in the text.

Condition	Mean latency (ms)		Difference	std. err.	p value
	Non-error	Error			
No Distractor	294.9	218.9	-76	21	p = 0.005
Synchronous Distractor	335.9	274.4	-61	4	p<0.00002
Asynchronous Distractor	344.9	229.0	-116	19	p = 0.0001

We analysed the asynchronous data alone to answer the following questions: 1) Does the recency of the last distractor event affect the latency for a target or distractor event movement? 2) Does the relative location of the distractor affect target latency? Put another way, if the distractor is in the opposite direction from the target, do latencies differ from the cases where the distractor lies in the same direction as the target? In each case, we found no significant difference or trend.

**Fig 10:**
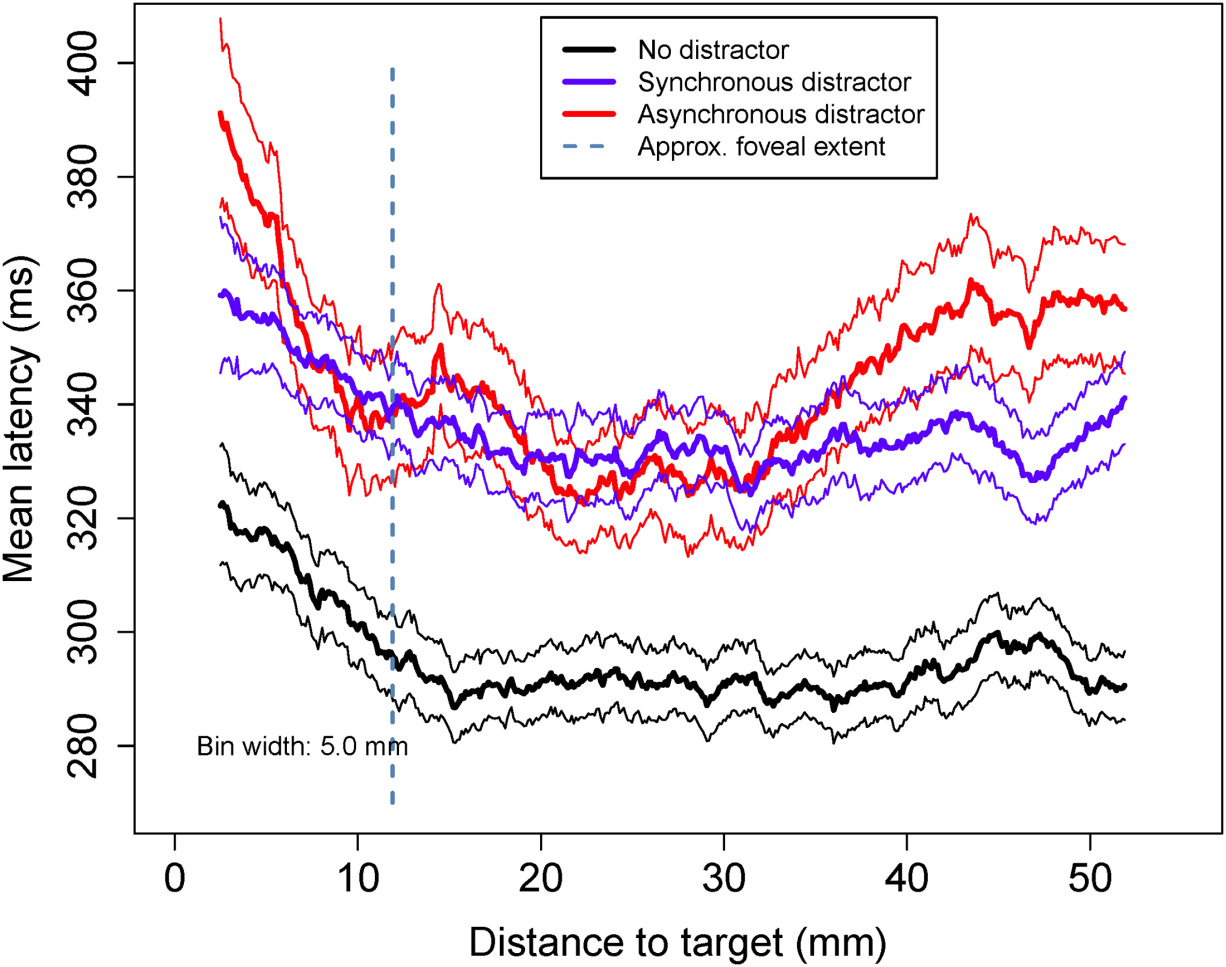
‘Moving bin’ mean latencies to first movement versus distance from stylus to target. A bin of width 5 mm is moved through the data. At each distance, the mean is computed (thicker lines) and the standard error of the mean is computed using the bootstrap method. The thinner lines show the corresponding 95% confidence intervals about the mean. The approximate location of the foveal region is shown by the dashed line, assuming that the eye to screen distance is 400 mm. A marked increase in latency is seen for all conditions within the foveal region. In the non-foveal region, distance is predictive only for the asynchronous distractor.

To examine whether the latency has a dependence on the distance between the stylus starting position and the target, we arranged all the latency values in order of distance to target and then computed a moving mean, using a bin with a width corresponding to 5 mm on the screen. At each bin position, we compute both the mean, and the 95% confidence interval by bootstrapping. Fig 10 shows the result of this analysis. We assume an approximate distance from eye to tablet of 400 mm, and take the fovea as having an effective radius of 1.7deg, at which point the cone density has dropped to 1/2e of its maximum [53] and rod density is comparable with cone density [54]. This boundary is shown as a dashed line in Fig 10. The graph shows that the mean no distractor latency is shorter than the latencies in the distractor conditions regardless of distance to target. There is a notable upward trend in latency at short distances, particularly within the high-acuity foveal region. At longer and very short distances to the target (greater than 30 mm or less than 7 mm) asynchronous latencies are longer. Analysing latency data for targets for which distance is in the non-foveal region, a regression analysis shows that ND and SD latencies are independent of distance, whereas the latency in the presence of asynchronous distractors *is* predicted by distance, F(1,1846) = 14.85, p<0.001.

**Fig 11:**
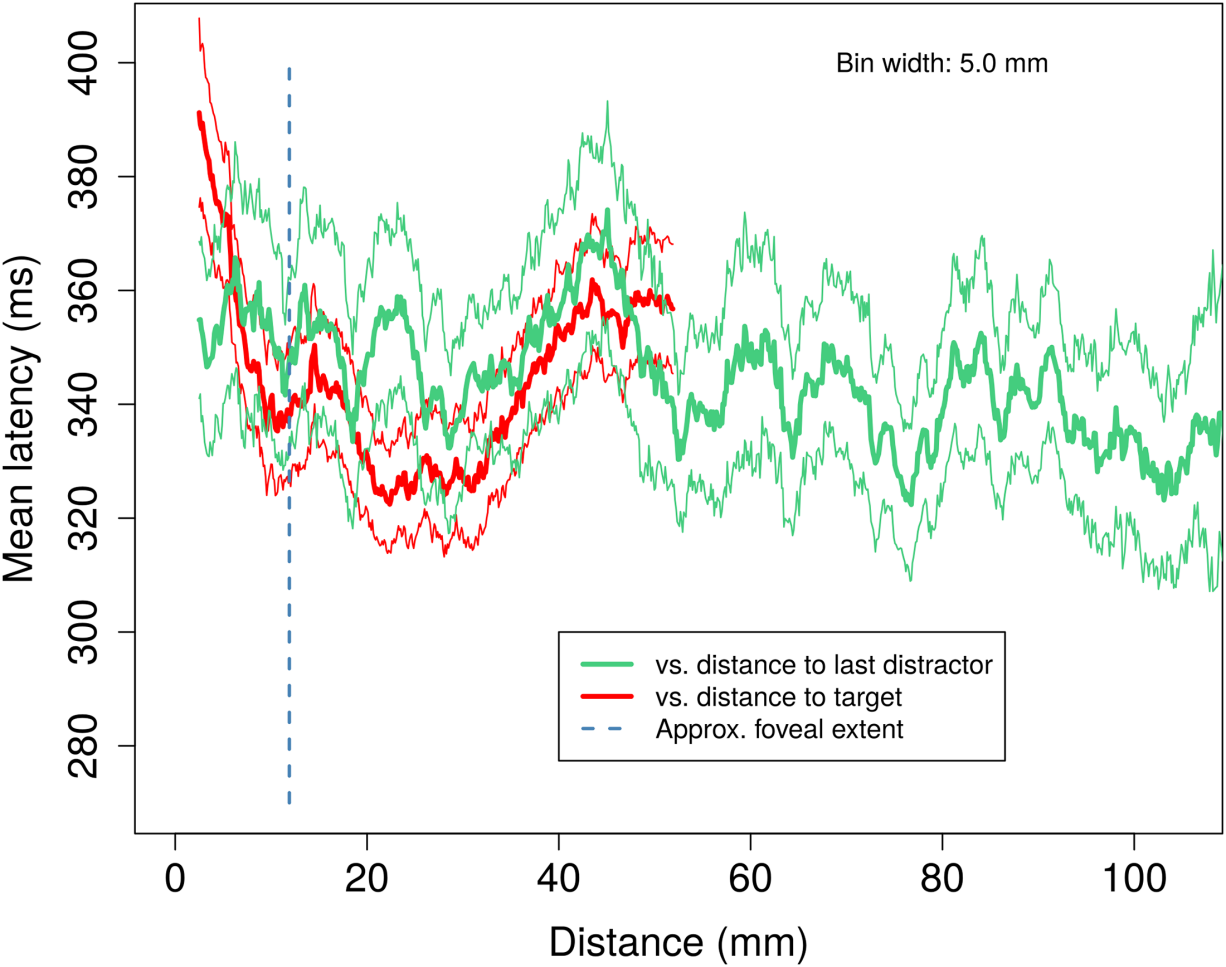
‘Moving bin’ mean latencies to first movement versus distance computed as in Fig 10 for the asynchronous distractor condition only. Here, latencies to first movement are shown with respect to the distance to the target (red), which is the same data as the red plot in Fig 10, along with latencies to first movement plotted with respect to the distance to the distractor which was visible when the target event occurred. As in Fig 10, the approximate location of the foveal region is shown by the dashed line.

We repeated this analysis to determine if there is any correlation between the absolute distance to the last distractor in the asynchronous condition and the latency to first movement. Fig 11 shows the result of this analysis and suggests a possible trend that as the last distractor becomes more distal, the latency reduces very slightly. This trend is not statistically significant, although a linear model reproduces the trend for this tiny effect (Slope -0.005 ms/mm, F(1,2230) = 2.799, p = 0.094).

### Error rates

We defined the error rate, *R*_*C*_, for an individual completing the line task in condition *C* as *the number of movement errors per target event*:

$$R_{C}=\frac{E_{C}}{T}$$ (8)

*E*_*C*_ is the total number of movement errors during the task and *T* is the number of target events (excluding those omitted from the latency extraction). Note that according to this definition, the asynchronous distractor error rate, *R*_*AD*_, could have a value exceeding 1, as movement errors could be caused both by target events and by distractor events. That is, the number of events in the asynchronous condition is:

$$N_{AD}=T+D$$ (9)

where *D* is the number of distractor events. The number of events in the synchronous (*N*_*SD*_) and no-distractor (*N*_*ND*_) conditions is smaller, and is given by:

$$N_{SD}=N_{ND}=T$$ (10)

Due to these additional distractor events, it was not possible to make a direct comparison between the error rate for the asynchronous distractor condition and those for the no-distractor and synchronous distractor conditions. Whilst the number of events is larger in the asynchronous condition, the nature of the events is different and so a definition of the error rate of *R*_*C*_ = *E*_*C*_/*N*_*C*_ would have no greater validity than Eq 8.

A Wilcoxon signed rank test between the ND and SD conditions indicated that the SD error rate was statistically significantly higher than the ND error rate (W = 23, p<10^−9^). Cliff’s Delta indicated a large effect size (|*d*| = 0.91).

**Fig 12:**
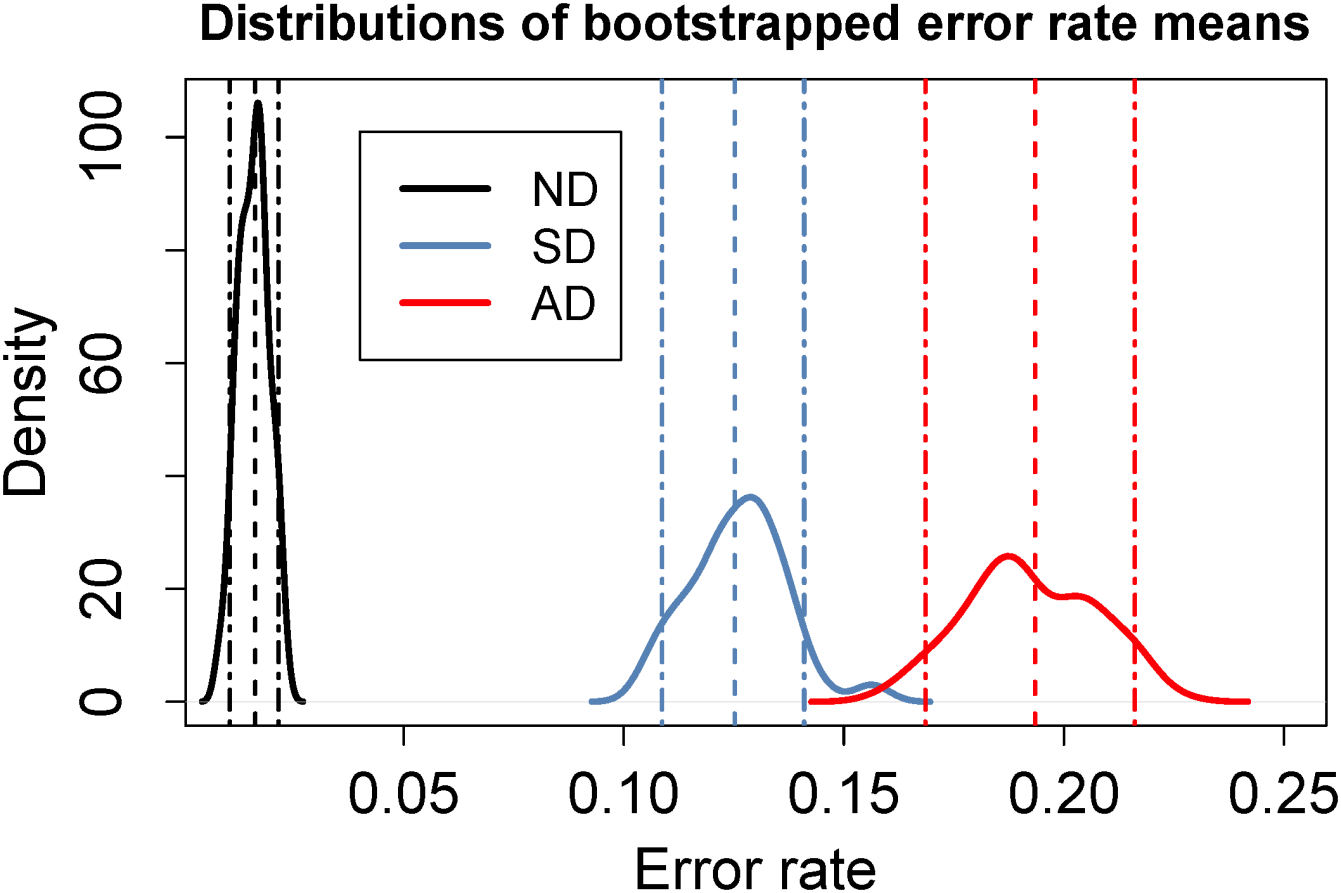
Probability density functions for the error rate sample means computed from 100 data resamples of 55 data points for each condition. The error rate is computed as the proportion of movement errors per target event. Data for the ND condition are shown in black and SD in blue. Dashed lines show the sample means; dot-dash lines show the 95% confidence intervals for the sample means. It is clear from this graph that the synchronous distractor increases the movement error rate compared with the no-distractor condition.

The error rates are given in Table 4 and Fig 12.

**Table 4:** Mean movement error rates with corresponding standard deviation and median absolute deviation statistics.

Condition	Mean error rate	Std. dev.	Med. abs. dev.
No Distractor	0.016	0.023	0.000
Synchronous Distractor	0.125	0.080	0.079
Asynchronous Distractor	0.193	0.119	0.097

## Discussion

We have presented a new, minimally complex task for the investigation of target-distractor reaching movements. The line task constrains arm movements to a two dimensional plane (because the stylus is constrained by the surface of the tablet) and guides the subject to carry out approximately one-dimensional reach movements. Because the nature of the movements is simple, it is easier to interpret the behaviour within an action-selection framework in which we consider the action-space to be one-dimensional.

As a tablet-based experiment, the line task can be conducted either within or outside the laboratory, with the potential for automated, large-scale data collection. Together with the task, we have provided algorithms which automate the process of extracting latency and movement error data from the raw trajectories.

The psychophysical results showed that latencies in either distractor condition were significantly longer than latencies in the no-distractor condition. This was in broad agreement with previous studies [2, 32, 44]. The results showed that latencies in the asynchronous distractor condition were slightly, but significantly longer than those in the synchronous condition and were dependent on the distance to the target, indicating the value of conducting an easy to deliver experiment with a large number of participants.

In both of the distractor conditions, latencies for error movements *towards* the distractor were significantly shorter than correct movements towards the target. Similarly, in the no distractor condition, latencies for error movements away from the target were significantly shorter than correct movements towards the target. Error rates were significantly higher in the synchronous condition when compared with the no-distractor condition. The error rate was significantly higher in the asynchronous condition, although as discussed, interpretation of this result is difficult because the event rate in the asynchronous condition is higher than in the other conditions.

Over the last four decades, much theoretical and experimental work has been carried out towards a goal of understanding action selection in the presence of environmental distractors. J. J. Gibson introduced the term *affordance* for an action which is physically possible and ‘afforded by the environment’ [55]. In our task, both target and distractor lines present affordances for movement. Gibson’s work inspired more recent theories of attention and action selection which we will discuss here; indeed, the theory to which we will devote most of our discussion is named the *affordance competition hypothesis*. These more recent theories make use of neurophysiological data collected from motor and pre-motor regions of the brain alongside behavioural measurements.

Although we are considering reach movements, it has been natural to consider action selection and attention in terms of the visual system and visual perception. Rizzolatti and co-workers presented psychophysical results both from choice selection and oculomotor experiments and developed a premotor theory of selective attention. In their theory, attention is intimitely bound to neural activity which is preparatory to goal-directed and spatially coded movements [56]. Although the theory makes the prediction that attended distractors may modify trajectories, it is not sufficiently quantitative to make predictions about the movement latencies which are the main topic of this paper.

Houghton, Tipper and co-workers produced a large body of work based on the idea that action and attention are linked [3, 27, 35, 44, 57, 58]. In their model of selective attention [57, 58], multiple objects are perceived in an ‘object field’. A competition occurs to determine the attended object. A ‘property matching’ system in the model selectively excites target-matching objects and inhibits non-target objects. The model makes a number of predictions which are not applicable to the line task (such as the negative priming and inhibition of return effects [57]), but it does predict that the existence of distractors will slow responses to targets, in agreement with our results.

Based on neurophysiological data from Georgopoulus, Kalaska and co-workers which indicated that neurons in motor cortex [59] and parietal cortex [60] produce a signal related to reach direction, Tipper, Howard and Jackson extended the ideas in the selective attention model to cover action selection in reach experiments [35]. In their new model, which is referred to as the ‘response vector model’ by Welsh and co-workers [31], the object field of the selective attention model becomes a field of potential reach vectors, interpreted as population codes. These population codes excite and inhibit one another via on-centre, off-surround excitation and inhibition. This assists in the competition between incompatible actions (such as reach left and reach right). Additional inhibitory processes selectively inhibit distractors, based on the subject’s goals (e.g. ‘ignore the red distractor’). This additional mechanism is required to prevent intermediate reaches between competing reach vectors which are too closely aligned for the off-centre inhibition mechanism to determine a winner.

Welsh & Elliott extended a discussion of the response vector model by considering the timing required by inhibitory processes. The response vector model does not incorporate a quantitative treatment of the time required to build up inhibitory suppression and so it is not able to predict how the effects of synchronous and asynchronous distractor presentation in the line task might differ and thus cannot help to explain the small difference in latencies which we detected between the SD and AD conditions in this work. The ‘response activation model’ [31] considers that the potential reach vectors found in overlapping regions of motor cortex participate in a race. When a movement is expressed, a component of the final movement will originate from the non-target, predicting trajectory deviations towards the non-target. In one experiment in which subjects were required to reach forwards over distances of up to 0.4 m, Welsh & Elliott [31] introduced a ‘stimulus-onset asynchrony’ between the onset of non-target and target stimuli. They found that if the non-target (indicated by colour) was illuminated 250 ms or 750 ms before the target, then response times were shortened by as much as 68 ms compared with a case in which there were no distractors. They argue that the most likely reason for this response facilitation is that the non-target induces motor activity which then aids the target in achieving sufficient activity to produce a movement. They go on to suggest that if this were true, then the non-target activity should be seen in trajectory variations. They presented trajectory data from their experiments which suggest that motor activity induced by the non-target would be inhibited by 750 ms after the non-target onset. We did not replicate their result; instead, we find that our asynchronous distractor condition, which produces a variable delay between distractor and target (mean(SD) 573(450) ms), *increases* the latency compared to the no distractor condition (by 50 ms; see Table 2). Note that there are important differences between the experiments; Welsh & Elliott presented targets over larger distances than those used in the line task and their subjects had to make reach movements forwards from their bodies, rather than sideways on a tablet.

A more recent model is the affordance competition hypothesis [1], which is qualitatively similar to the response vector and activation models but which has closer links to the neuroanatomy of reach and action selection. Under this hypothesis, potential actions are continously specified, based on incoming sensory information, whilst selection processes (both cortical and sub-cortical) simultaneously determine which are to be enacted. It extends previous ideas of evidence integration [61, 62] and sub-cortical action selection [10, 63, 64] by incorporating evidence that activity within motor cortical areas participates in action selection and specification.

The affordance competition hypothesis contrasts with some more traditional ideas in cognitive psychology, which describe staged or serial processes. In the traditional description, sensory information is first collected to build an internal representation of the external world. This representation, along with internal motivations, then determines or selects the next action. Finally, a process of action specification is engaged, controlling muscle activation to achieve the desired action. The difficulty with this framework is that neural activity relating to sensory information, motor specification and cognitive value judgements can be found within the same brain nuclei and are seen to build up concurrently within a task [23, 65].

The affordance competition hypothesis suggests that action selection begins at an early stage within motor cortical brain regions with activity building up in networks which specify multiple potential actions. Cortical and subcortical [66] processes take part in supression of non-optimal actions. The model of reaching decisions presented by Cisek [1, 67] and supported by neural activity reported in [65] shows activity in directionally sensitive neurons in posterior parietal cortex (PPC), dorsal premotor cortex (PMd), primary motor cortex (M1) and in ‘colour sensitive’ pre-frontal cortex (PFC). A spatial cue is first presented which indicates two possible direction options. With this cue, activity is first seen to build in the neurons in PPC and PMd which encode these two directions. Later, a colour cue indicates which direction will provide the reward. On presentation of this colour cue, activity for the correct direction is driven by activity in ‘colour-sensitive’ pre-frontal cortex, whilst neuronal activity encoding incorrect directions is supressed. Finally a ‘go’ cue causes a further increase in activity in PMd and ungates the connection from PMd to primary motor cortex (M1), providing the motor command. The model represents a type of sequential evidence accumulation model [62], in that evidence for competing actions is repeatedly sampled until one action reaches a threshold and is un-gated.

We now see fit to interpret our task in this framework. In the synchronous distractor condition of the line task, each event is a two-alternative forced choice between potential moves towards red and cyan lines. It is similar to the experimental condition described in Fig 3(c) in [1] in which two choices are first presented, then a colour cue indicates the correct target. In our task, the cyan line is always the target, and so we would expect the details of the evidence accumulation to differ. We might expect to see activity in neurons encoding a move towards the cyan target line build up faster than activity in those encoding a move towards the red distractor. The direction of movement made, and whether or not this is an error will depend on the *rate* at which evidence of the colour of the lines builds up, the *magnitude of the activity threshold* required to un-gate a movement and the *amplitude of noise* within the system. The no-distractor condition provides a control condition in which there is no activity (other than noise) in competing neurons. The model of Cisek [1] predicts that the presence of the distractor will increase the latency to first movement in the synchronous distractor condition because cross-inhibition between competing potential actions (‘move left’ and ‘move right’) will reduce the rate of evidence accumulation in motor cortical areas.

It is not clear, however, that the affordance competition hypothesis predicts a shorter latency for error movements; only that evidence for error movements can be detected as early as activity is seen in PMd and PPC. The response vector/activation models are similarly unable to predict this finding. To explain this finding we would require either that the rate of evidence accumulation caused by the distractor line is greater than the rate caused by the target line, or that the threshold to un-gate movement differed for the distractor line. Neither of these seem plausible, though a colour effect should be ruled out by reversing the colours used for target and distractor and repeating the measurements.

It is interesting to consider what Cisek’s model would predict for in the two different distractor conditions and whether it has the ability to predict the latency difference which we have observed. In Cisek’s model, visual input is passed into PPC, with a short time constant of 0.3 s, and also into colour-sensitive PFC, with a much longer time constant of 100 s (See Fig 1.A from [67] and the related supplementary material from that paper). We would expect to see activity in the red-sensitive PFC relating to the distractor line and in the blue sensitive PFC for the target line along with activity for both lines in PPC. The PFC output interacts multiplicatively with PPC output to produce input to PMd layers of the model. Given the long time constant in the PFC, it could be argued that activity from previous distractors would persist during the presentation of a new target line and slow the decision making process in PMd. However, the behaviour of PFC is not modelled in detail in the Cisek model, and instead, the determination of which colour refers to a target is made by providing excitatory input to one colour-sensitive PFC population or the other. In the Cisek modelling, this occurs on the presentation of the colour code (a central red or blue circle). Given that our experiment is carried out in blocks, and the target is always blue, there would be a constant excitatory input to the blue-sensitive PFC, which would suppress activity in red PFC (red PFC neurons inhibit blue PFC neurons and vice-versa). It is hard to see how much activity could build up in PPC relating to the distractor, due to the multiplicative enhancement of the signal to PMd, and the feedback from PMd to PPC. To apply the Cisek model to our task, it would likely be necessary to increase the noise level in the PFC relative to the amount of constant excitatory input, otherwise the model would not reproduce the errors seen in the experiment. In any case, we might expect the concurrent presentation of the distractor in the synchronous condition to have a more competitive effect, slowing the decision making process, when compared with the asynchronous condition; a reverse of the observed trend.

The threshold required for activation of M1 is thought to be governed by activity in related thalamo-cortical loops through the basal ganglia [66, 68]. Channels have been identified in these loops [69], and have been postulated to carry motor commands [70–72]. Output nuclei of selected channels through the basal ganglia are disinhibited, enabling the execution of actions via brainstem [73] and cerebellar [74] activity. The same reach movement could be selected in any of the no-distractor, synchronous and asynchronous conditions of the line task; for example, the target could specify a movement to the left of 40 mm. In all conditions, the same channel in basal ganglia is (presumably) selected. The architecture of the basal ganglia ensures that the threshold required to select the channel is kept relatively constant [75, 76], regardless of the number of competing inputs. Using this argument, we suggest that an increase in the un-gating threshold activity in PMd which would be observed in the asynchronous condition of the line task is unlikely.

In order to explain a lengthened asynchronous latency, the evidence accumulation seen in PMd should be slowed, which could be achieved with a blanket inhibition of PMd provided by higher cortical areas. This could be driven by experiencial learning that in the asynchronous condition, early evidence from edge detection regions of visual cortex does not always imply the need for a movement, as it does in the synchronous condition.

The simplicity of the design of the line task means that the influence of distractor interference on action selection mechanisms can be probed in large-scale subject populations. Tracking tasks are valuable for the study of sensory motor function, enabling the collection of objective and quantitative data, as well as producing detailed information about task performance. It is not necessary to provide training to carry out tracking tasks, as making point or reach movements is entirely natural. Tracking tasks can be equally well completed by patients with neurological disorders [77] as by healthy subjects.

The employment of experimental tracking tasks means that considerable control can be held over stimuli, such as the signalling of targets and distractors [78]. In spite of this, Watson and colleagues [78] suggest that two dimensional tasks provide more precise measurements and considerably more information regarding sensory-motor function. Moreover, it is suggested that two dimensional tracking tasks are more sensitive than one dimensional tracking tasks in their ability to detect dysfunctional motor performance within Parkinson’s patients. The apparent limitation of using one dimensional rather than two dimensional pursuit tracking tasks should be considered in future research.

The line task can be accused of lacking ecological validity, in that it does not represent tasks common to everyday life: In the line task, reaches are small (limited by the tablet size) and follow one dimensional lines as targets; in daily life, reaches may be large (up to 0.5 m) and are usually made to three dimensional targets for grasping. The line task purposefully trades ecological validity for a simple environment with few potentially confounding factors, as discussed in the introduction.

Future implementations of the line task could vary aspects of the distractor’s parameters. For example, the rate of distractor event presentation, the number of distractor lines or distractor and target thicknesses could be varied. A future experiment should be run to exclude the possibility that there is a colour effect, with the target and distractor colours being reversed for some subjects, or between conditions. Gaps between target and distractor presentation could be investigated, analogous to the ‘gap, step and overlap’ paradigm used in saccadic eye movement experiments. Investigations in which the target position was updated *during* the initial stylus movement could give insight into selection and online error correction within motor systems. Whilst not discussed here, the dynamics of the trajectories recorded by the line task could be analysed in detail, with particular attention being paid to the ‘first smooth movement’. In order to more carefully study the effect of the distance to the target, it would be necessary to record or control the distance from each subject’s eyes to the tablet surface. Consideration should always be given to learning effects in each condition of the line task [79]. Here, the order in which conditions were presented was randomised, but it may be preferable to implement an independent measures design, with each group completing the opposite order of distractor conditions.

In conclusion, the line task is a tablet-based application that can be used to record data for healthy or neurologically impaired subjects in the laboratory, clinic or at home. As such, it is an effective and convenient tool for the detailed investigation of action selection for reaching movements.

## Supporting information

S1 Appendix A list of omit reasons. A list of the reasons for which it may have been impossible to extract a latency and error measurement for an event.
(PDF)

S2 Appendix Statistical analysis. PDF version of statistical analysis ipython notebook.
(PDF)
