# Supplementary material for: Target-distractor synchrony affects performance in a novel motor task for studying action selection
Source: PLoS One. 2017 May 5;12(5):e0176945. doi: 10.1371/journal.pone.0176945 (PMC5419578; doi:10.1371/journal.pone.0176945)
Supplement: S1 Appendix — A list of the reasons for which it may have been impossible to extract a latency and error measurement for an event. (PDF) [file pone.0176945.s001.pdf]

Event omission reasons in the line task

Sebastian James

This is supplementary information for the paper entitled ‘Target-distractor Synchrony Affects Performance in a Novel Motor Task for Studying Action Selection’.

The full list of reasons for omission is given below, with the descriptive code for the reason in bold typeface and the number of occurrences (out of 17617 events) in italics.

Target position change less than minimum jump size: If the target line’s position changed less than a threshold of 20 pixels, it was omitted from analysis. This was used to allow the output of the script to be compared with an alternative method for measuring latency and error rates, described later. *410 occurrences.*

Stable position later than event onset: If the stylus did not achieve a stable pre-event position, then it was not safe to measure a latency for the movement for the event. This usually occurred when two target events followed each other in quick succession and the subject had not had time to reach the first target position before it moved for a second time. *120 occurrences.*

Too fast (targ) and ***Too fast (distractor):*** Any event with a movement latency below 100 ms (the value of the parameter A.fastest_brain_decision in the lt_analyse_latency.m script) was omitted. Previous research suggests that participants cannot make movements faster than this [1, 2]. The cause of such apparently short latencies in the raw data was usually the lack of a stable pre-event stylus position; the subject was not able to settle at the previous target position. *130 occurrences (target), 20 occurrences (distractor).*

No movement detected No movement was detected for a target event, and therefore no latency could be determined. This may have occurred if the target position change was very small. *21 occurrences.*

Stable stylus period too short If the stable stylus position period was less than 200 ms for targets (A.min_stableposition_period[illegible] or 150 ms for distractors (A.min_stableposition_period_dist) then the event was omitted, which is to say that a latency was measured only if the stylus remained in a stable position for at least the above periods of time. It was the second most common reason for omitting an event from latency and error measurements. *731 occurrences (target), 1743 occurrences (distractor).*

Drift too great during stable stylus period If the stylus position at the end of the stable stylus period had moved more than 15 px (A.max_move_thresh) with respect to the stylus position at the start of the stable stylus position period, then the event was omitted with this reason. *21 occurrences (target), 18 occurrences (distractor).*

Drift too great during stable period (avg) If the average speed of the stylus during the stable stylus period was greater than a threshold of 0.01 px/ms (0.011 cm/s) for a target event (A.max_avg_drift_speed) or 0.02 px/ms (0.023 cm/s) for a target event (A.max_avg_drift_speed_dist) then the event was omitted. Because the mean time between target events was set to 1.2 s, a fast rate, this was the most common reason for omitting movements from analysis. *1820 occurrences (target), 957 occurrences (distractor).*

Movement occurs beyond next target This omit reason was recorded for a distractor event when any detected motion occurred after the next target event. It means that the subject did not make an error movement, and therefore no latency could be measured. However, these distractor events *were* included when computing the proportion of distractor events which caused an error movement. *1756 occurrences (distractor).*

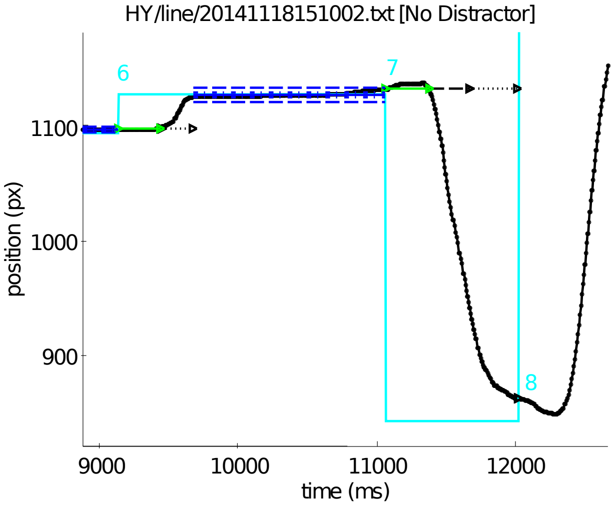

Figure 1: An example event for which the omission reason was "Drift too great during stable period (avg)". Here, the latency onset for events 6 and 7 were determined, but the stylus (black line) did not become stationary at the target position (cyan line) prior to event number 8 and so it was omitted from latency and error analysis. For the meaning of line colours, refer to Fig. 2 in the paper

Subject was distracted by closely previous distractor These last four reasons for omission concern the case where there was an asynchronous distractor and a distractor event and target event occur temporally close together. In this particular case, either the target or the distractor may have caused the movement; both lines were displaced in the same direction with respect to the stable stylus position. The distractor event occurred before the target event. Because the recorded motion was *away* from the target, the target was omitted and the event was recorded as a distraction in the earlier distractor event. *4 occurrences (target).*

Incorrect move was recorded in previous distractor event In this case, the target and distractor lines were located in opposite directions from the initial stylus position; again, the distractor line occurred first. The stylus initially followed the distractor and so an incorrect movement was recorded in the distractor event, and the target event was omitted with this reason code. *4 occurrences (target).*

This distractor event did not distract the stylus movement In this case, the target event led the distractor event and they had opposite directions. The stylus successfully followed the target event and so the distractor event, which occurred soon after was omitted with this reason. *66 occurrences (distractor).*

Recorded this stylus movement as a distraction towards the next distractor Again, the target event led the distractor event and they had opposite directions. Here, the stylus followed the distractor direction and so to avoid recording a second movement error, the target event was omitted. *4 occurrences (target).*

References

1. Gielen C, Van den Heuvel PJM, Van Gisbergen JAM. Coordination of fast eye and arm movements in a tracking task. Experimental Brain Research. 1984;56(1):154–161. Available from: http://link.springer.com/article/10.1007/BF00237452.
2. Prablanc C, Echallier JF, Komilis E, Jeannerod M. Optimal response of eye and hand motor systems in pointing at a visual target. Biological cybernetics. 1979;35(2):113–124. Available from: http://link.springer.com/article/10.1007/BF00337436.
