# Supplementary material for: Target-distractor synchrony affects performance in a novel motor task for studying action selection
Source: PLoS One. 2017 May 5;12(5):e0176945. doi: 10.1371/journal.pone.0176945 (PMC5419578; doi:10.1371/journal.pone.0176945)
Supplement: S2 Appendix — PDF version of statistical analysis ipython notebook. (PDF) [file pone.0176945.s002.pdf]

Anova

February 15, 2017

1 Statistical analysis of Line Task 2014 data

20151112-20170215

Author: Seb James seb.james@sheffield.ac.uk

This is a companion analysis notebook for the paper *Target-distractor Synchrony Affects Performance in a Novel Motor Task for Studying Action Selection*

This notebook uses data which was generated by the Octave/Matlab script lt_analyse_all.m.

It does some analysis itself, but it also passes much analysis off to R scripts, the output of which it then presents. It is rather unparsemonious to have used three coding environments to have analysed the data from the line task; in retrospect I would probably have limited this to two (Octave and R).

1.1 Data import

Import latency and error data which is computed from the raw data by the script lt_analyse_all.m. This script saves the resulting data into a Matlab v7 file.

See the Analysing Data.ipynb notebook for details about how the data is organised. This notebook contains just the analysis.

This script requires the python modules scipy and statsmodels. It also makes external system calls to evaluate R scripts and so R must also be installed together with the R modules effsize and nlme.

```
In [8]: # Import the data, which should be available in Matlab v7 format.
        # fnames is used throughout the rest of this notebook, so this section nee
        import scipy.io as sio
        mat_workspace = sio.loadmat('AllData/fnames.mat')
        fnames = mat_workspace['fnames']
        print 'fnames.mat has been imported.'
        # We need Image from IPython.display to view graphs
        from IPython.display import Image
```

```
fnames.mat has been imported.
```

1.1.1 On subject omission

Omitted subjects, with reasons:

EM1, KW, JD, LC, YC - Experimenter chose the wrong jump time on one or more conditions.

AB2_ - Experimenter did not carry out a Synchronous Distractor trial

is132 - Age not recorded for this participant - could not match data to participant in Spreadsheet.

After omitting these subjects, 55 remained.

1.1.2 On event omission

The script lt_analyse_latency.m will try to produce accurate latencies to first movement, along with direction errors from the time series data in the 2014*.txt files. It omits some events for a set of reasons which are described in the paper (grep for omit_reason in the .m code to find them and see the omit_reasons() function in the analysis code below).

Later, when the individual analysis is called, a standard approach to outlier removal is taken (see individual.excludeOutliers()).

1.2 Count the number of target events

This next section of code tallies up the number of target events in each task. This is *not* the number of latency measurements obtained from the data by the Octave pre-processing script.

```
In [9]: from __future__ import division
        import numpy as np

        # Find event counts
        import scipy.io as sio
        mat_workspace2 = sio.loadmat('AllData/eventcounts.mat')
        evcounts = mat_workspace2['fnames']

        num_targs = []
        nd_num_targs = []
        sd_num_targs = []
        ad_num_targs = []
        min_nd_num_targ = 1000
        min_sd_num_targ = 1000
        min_ad_num_targ = 1000
        min_nd_num_targ_idx = ''
        min_sd_num_targ_idx = ''
        min_ad_num_targ_idx = ''
        max_nd_num_targ = 0
        max_sd_num_targ = 0
        max_ad_num_targ = 0
        max_nd_num_targ_idx = ''
        max_sd_num_targ_idx = ''
        max_ad_num_targ_idx = ''

        allevents = dict()

        # Note: This code written before individual.num_target_events() and friends
        for task in zip(*evcounts):
```

```
        num_targ = task[1][0][0]
        cond = task[2][0]
        subj = task[0][0].split('/',2)[1]
        #print 'Subj',subj,'Num target events:',num_targ, 'Condition:',cond

        if cond == "No Distractor":
            nd_num_targs = np.append(nd_num_targs, num_targ)
            allevents[subj+"nd"] = num_targ
            if num_targ < min_nd_num_targ:
                min_nd_num_targ = num_targ
                min_nd_num_targ_idx = task[0][0]
            if num_targ > max_nd_num_targ:
                max_nd_num_targ = num_targ
                max_nd_num_targ_idx = task[0][0]
        elif cond == "Synchronous Distractor":
            sd_num_targs = np.append(sd_num_targs, num_targ)
            allevents[subj+"sd"] = num_targ
            if num_targ < min_sd_num_targ:
                min_sd_num_targ = num_targ
                min_sd_num_targ_idx = task[0][0]
            if num_targ > max_sd_num_targ:
                max_sd_num_targ = num_targ
                max_sd_num_targ_idx = task[0][0]
        elif cond == "Asynchronous Distractor":
            ad_num_targs = np.append(ad_num_targs, num_targ)
            allevents[subj+"ad"] = num_targ
            if num_targ < min_ad_num_targ:
                min_ad_num_targ = num_targ
                min_ad_num_targ_idx = task[0][0]
            if num_targ > max_ad_num_targ:
                max_ad_num_targ = num_targ
                max_ad_num_targ_idx = task[0][0]
        num_targs = np.append(num_targs, num_targ)

    print 'Mean number of target events:'
    print 'Overall:',num_targs.mean(),'(',num_targs.std(),')', 'ND:',nd_num_tar
    print 'Min nums:'
    print 'ND:',min_nd_num_targ,min_nd_num_targ_idx,'SD:',min_sd_num_targ,min_s
    print 'Max nums:'
    print 'ND:',max_nd_num_targ,max_nd_num_targ_idx,'SD:',max_sd_num_targ,max_s
```

```
Mean number of target events:
Overall: 64.703030303 ( 6.8721874621 ) ND: 65.5636363636 SD: 64.8545454545 AD: 63.6
Min nums:
ND: 56.0 Aizat/EB1/line/20141204160911.txt SD: 42.0 Katie/JS/line/20141117163927.tx
Max nums:
ND: 75.0 Rachel/AW1_/line/20141118163434.txt SD: 75.0 Rachel/EB2/line/2014111816573
```

1.3 Analysis code

The following code block provides a number of global functions and a class which are used in the rest of this analysis. It's the majority of the code used in the analysis here.

The class called *individual* stores the latency data for each individual and has a set of methods for analysing the data.

```
In [10]: from __future__ import division

         import numpy as np
         import random

         def getfnameid (filename):
             # idarr[1] is the ID, idarr[0] is the experimenter, idarr[3] is the da
             idarr = filename.split('/')
             return idarr[1]

         # From the condition string, return an index for the condition. 0 is
         # "No Distractor trial", 1 is "Synchronous Distractor trail", 2 is
         # "Asynchronous Distractor trial".
         def getcondition (condition_string):
             condition_index = -1
             if 'No Dist' in condition_string:
                 condition_index = 0
             elif 'Synchro' in condition_string:
                 condition_index = 1
             elif 'Asynchr' in condition_string:
                 condition_index = 2
             return condition_index

         # A Single Factor ANOVA calculation for three datasets
         def group_anova(nodist_latencies,sync_latencies,async_latencies):

             all_latencies = np.concatenate((nodist_latencies, sync_latencies, asyn

             # Compute grand mean
             grand_mean = all_latencies.mean()
             #print 'Grand mean:',grand_mean,'(',all_latencies.var(ddof=1),') not

             # Compute within-group variance
             tmp1 = all_latencies
             np.power(tmp1, 2)
             within_group_dof = all_latencies.size-3
             within_group_variance = tmp1.sum()/within_group_dof
             #print 'within_group_variance',within_group_variance
```

```
    nodist_mean = nodist_latencies.mean()
    sync_mean = sync_latencies.mean()
    async_mean = async_latencies.mean()

    # Compute amoung-group variance
    tmp1 = np.power (grand_mean - nodist_mean, 2)*nodist_latencies.size
    tmp2 = np.power (grand_mean - sync_mean, 2)*sync_latencies.size
    tmp3 = np.power (grand_mean - async_mean, 2)*async_latencies.size
    sosquares = tmp1 + tmp2 + tmp3
    between_group_dof = 2 # 3 conditions => 3 groups, so 3-1 DOF
    between_group_variance = sosquares / between_group_dof
    #print 'between_group_variance',between_group_variance

    # Now compute the F ratio
    F = between_group_variance/within_group_variance

    # Lastly, what's the probability for this?
    P = 1-special.fdtr(between_group_dof,within_group_dof,F)

    return (F, between_group_dof, within_group_dof, P)

# Convert an omit reason id to a reason string
def omit_reason (or_id):
    reason_str = ""
    if or_id == 0:
        reason_str = "0 not omitted"
    if or_id == 1:
        reason_str = "1 target posn change insignificant"
    elif or_id == 2:
        reason_str = "2 target posn held less than min. duration"
    elif or_id == 3:
        reason_str = "3 target posn change less than min. jump size"
    elif or_id == 4:
        reason_str = "4 Stable position later than event onset"
    elif or_id == 5:
        reason_str = "5 Another event caused this movement"
    elif or_id == 6:
        reason_str = "6 Not distracted"
    elif or_id == 7:
        reason_str = "7 Too fast (targ)" # faster than A.fastest_brain_de
    elif or_id == 8:
        reason_str = "8 Too fast (distractor)" # faster than A.fastest_bra
    elif or_id == 9:
        reason_str = "9 No movement detected"
    elif or_id == 10:
        reason_str = "10 Failed to find stable stylus posn"
    elif or_id == 11:
        reason_str = "11 Stable stylus period too short"
```

```
    elif or_id == 12:
        reason_str = "12 Drift too great during stable stylus period"
    elif or_id == 13:
        reason_str = "13 Drift too great during stable period (avg)"
    elif or_id == 14:
        reason_str = "14 Stylus moving at event onset"
    elif or_id == 15:
        reason_str = "15 Stylus didn't move away from target"
    elif or_id == 16:
        reason_str = "16 Movement occurs beyond next target"
    elif or_id == 17:
        reason_str = "17 Subject was distracted by closely previous distra
    elif or_id == 18:
        reason_str = "18 Incorrect move was recorded in previous distracto
    elif or_id == 19:
        reason_str = "19 This distractor event did not distract the stylus
    elif or_id == 20:
        reason_str = "20 Recorded this stylus movement as a distraction to

    return reason_str

# Take n sub-samples from distn
def subsample (distn, n):
    distn_cp = distn
    np.random.seed(19742016)
    counter = 0
    subsamp = []
    if n > len(distn_cp):
        print 'No possible to get',n,'samples from this distribution!'
        return subsamp
    while counter < n:
        i = 0
        for s in distn_cp:
            if np.random.uniform()>0.5:
                np.delete(distn_cp, i)
                i -= 1
                subsamp = np.append(subsamp, s)
                counter = counter + 1
            if counter >= n:
                break
            i += 1
    return subsamp

def compute_mad_outliers (points, thresh=3.5):
    """
    Returns a boolean array with True if points are outliers and False
    otherwise.
```

```
    Parameters:
    -----------
        points : An numobservations by numdimensions array of observations
        thresh : The modified z-score to use as a threshold. Observations
            a modified z-score (based on the median absolute deviation) gr
            than this value will be classified as outliers.

    Returns:
    --------
        mask : A numobservations-length boolean array.

    References:
    ----------
        Boris Iglewicz and David Hoaglin (1993), "Volume 16: How to Detect
        Handle Outliers", The ASQC Basic References in Quality Control:
        Statistical Techniques, Edward F. Mykytka, Ph.D., Editor.
    """
    if len(points.shape) == 1:
        points = points[:,None]
    median = np.median(points, axis=0)
    # Compute root mean square deviation from median:
    diff = np.sum((points - median)**2, axis=-1)
    diff = np.sqrt(diff)
    # The median of this is the median abs. deviation from the median.
    med_abs_deviation = np.median(diff)
    # A modified Z score is analogous to the Z score for number of SDs fro
    modified_z_score = 0.6745 * diff / med_abs_deviation

    return modified_z_score > thresh

def compute_mad (points):
    """
    Returns the median abs deviation value for the points

    Parameters:
    -----------
        points : An numobservations by numdimensions array of observations

    Returns:
    --------
        med_abs_deviation: The median absolute deviation

    References:
    ----------
        Boris Iglewicz and David Hoaglin (1993), "Volume 16: How to Detect
        Handle Outliers", The ASQC Basic References in Quality Control:
        Statistical Techniques, Edward F. Mykytka, Ph.D., Editor.
    """
```

```
    if len(points.shape) == 1:
        points = points[:,None]
    median = np.median(points, axis=0)
    # Compute root mean square deviation from median:
    diff = np.sum((points - median)**2, axis=-1)
    diff = np.sqrt(diff)
    # The median of this is the median abs. deviation from the median.
    med_abs_deviation = np.median(diff)
    return med_abs_deviation

# Libs used in class individual
from scipy import special
from scipy import stats
import statsmodels.api as sm
from matplotlib import pyplot as plt
import matplotlib.lines as mlines

# A class for an individual's data.
class individual:
    def __init__(self, subj_id):

        # Hindsight note: I should have made an "event" class.

        self.subj_id = subj_id;
        self.idnum = -1
        self.filename = ""

        # Default number of subsamples to take from a distribution
        self.numSubsamples = 20

        # All data, errorred and ok, for each condition.
        self.alldata_nd = np.ndarray(0)
        self.alldata_sd = np.ndarray(0)
        self.alldata_ad = np.ndarray(0)

        # These are the "non-movement-error target latencies"
        self.nodist_latencies = np.ndarray(0)
        self.sync_latencies = np.ndarray(0)
        self.async_latencies = np.ndarray(0)
        self.nodist_latencies_rank = np.ndarray(0)
        self.sync_latencies_rank = np.ndarray(0)
        self.async_latencies_rank = np.ndarray(0)

        # These are the "non-movement-error distractor latencies"
        self.nodist_dgood_latencies = np.ndarray(0)
        self.sync_dgood_latencies = np.ndarray(0)
        self.async_dgood_latencies = np.ndarray(0)
        self.nodist_dgood_latencies_rank = np.ndarray(0)
```

```
        self.sync_dgood_latencies_rank = np.ndarray(0)
        self.async_dgood_latencies_rank = np.ndarray(0)

        # These are the "target event error latencies"
        self.nodist_err_latencies = np.ndarray(0)
        self.sync_err_latencies = np.ndarray(0)
        self.async_err_latencies = np.ndarray(0)
        self.nodist_err_latencies_rank = np.ndarray(0)
        self.sync_err_latencies_rank = np.ndarray(0)
        self.async_err_latencies_rank = np.ndarray(0)

        # These are the "distractor event error latencies" (N/A for ND or
        self.nodist_derr_latencies = np.ndarray(0)
        self.sync_derr_latencies = np.ndarray(0)
        self.async_derr_latencies = np.ndarray(0)
        self.nodist_derr_latencies_rank = np.ndarray(0)
        self.sync_derr_latencies_rank = np.ndarray(0)
        self.async_derr_latencies_rank = np.ndarray(0)

        self.async_derr_timesince = np.ndarray(0)
        self.async_tnoerr_timesince = np.ndarray(0)

        self.nodist_dirns = np.ndarray(0)
        self.sync_dirns = np.ndarray(0)
        self.async_dirns = np.ndarray(0)

        self.n_errors_per_target_nd = 0
        self.n_errors_per_distractor_sync = 0
        self.n_errors_per_distractor_async = 0

        # Number of distractor events in the asynchronous case
        self.n_async_distractors = 0

        # Some constants
        self.DIST_EVENT = 0
        self.TARG_EVENT = 1

    def csvheader(self):
        hl = 'Experimenter,Subject,Distractor type,RT (M),RT (SD),N correc
        hl += 'RT incorrect (SD),N incorrect,Error rate,D1 (-ve=>error)'
        for i in range(2,65):
            hl += ',D{0}'.format(i)
        hl += '\n'
        return hl

    def ad_omit_reasons (self, event_type):
        event_indices = [i for i,elem in enumerate(self.alldata_ad[:,1]) i
        # Last col gives omit reason
```

```
        return np.bincount(self.alldata_ad[event_indices,6].astype(int),mi

    def sd_omit_reasons (self, event_type):
        event_indices = [i for i,elem in enumerate(self.alldata_sd[:,1]) i
        # Last col gives omit reason
        return np.bincount(self.alldata_sd[event_indices,6].astype(int),mi

    def nd_omit_reasons (self, event_type):
        event_indices = [i for i,elem in enumerate(self.alldata_nd[:,1]) i
        # Last col gives omit reason
        return np.bincount(self.alldata_nd[event_indices,6].astype(int),mi

    # Experimenter,Subject,Distractor type,RT (M),RT (SD),N correct,RT inc
    # RT incorrect (SD),N incorrect,Error rate,Delays (negative = incorrec
    def csvlineset(self):
        line = self.filename_nd + ',' + self.subj_id + ',0,'
        # ND mean/SD. NB: /1000 to output numbers as seconds, like Mauro
        line += '{0},{1},'.format(self.nodist_mean()/1000,self.nodist_late
        # ND RT incorrect mean/SD
        line += '{0},{1},{2},'.format(self.num_tgood(0),self.nodist_err_me
        # ND num incorrect, error rate
        line += '{0},{1}'.format(self.num_tmoveerrors(0),ind.num_tmoveerro
        # ND delays
        #for i in self.nodist_latencies[:,1]:
        #    line += ',{0}'.format(i/1000)
        for i in self.alldata_nd:
            if i[5]==1:
                line += ',OR{0}'.format(omit_reason(i[6]))
            else:
                if i[2]==0: # ND and no error
                    line += ',{0}'.format(i[4]/1000)
                elif i[2]==1: # ND and error
                    # make errorred ones -ve as Mauro does.
                    line += ',{0}'.format(-i[4]/1000)

        line += '\n'

        line += self.filename_sd + ',' + self.subj_id + ',1,'
        # SD mean/SD
        line += '{0},{1},'.format(self.sync_mean()/1000,self.sync_latencie
        # SD RT incorrect mean/SD
        line += '{0},{1},{2},'.format(self.num_tgood(1),self.sync_err_mean
        # SD num incorrect, error rate
        line += '{0},{1}'.format(self.num_tmoveerrors(1),ind.num_tmoveerro
        # SD delays
        for i in self.alldata_sd:
            if i[5]==1:
                line += ',OR{0}'.format(omit_reason(i[6]))
```

```
            else:
                if i[2]==0: # SD and no error
                    line += ',{0}'.format(i[4]/1000)
                elif i[2]==1: # SD and error
                    # make errorred ones -ve as Mauro does.
                    line += ',{0}'.format(-i[4]/1000)
        line += '\n'

        line += self.filename_ad + ',' + self.subj_id + ',2,'
        # AD mean/SD
        line += '{0},{1},'.format(self.async_latencies.mean()/1000,self.as
        # AD RT incorrect mean/SD
        line += '{0},{1},{2},'.format(self.num_tgood(2),self.async_err_mea
        # AD num incorrect, error rate
        line += '{0},{1}'.format(self.num_tmoveerrors(2),ind.num_tmoveerro
        # AD delays
        for i in self.alldata_ad:
            if i[5]==1:
                line += ',OR{0}'.format(omit_reason(i[6]))
            else:
                if i[1]==1 and i[2]==0: # TARG_EVENT and AD and no error
                    line += ',{0}'.format(i[4]/1000)
                elif i[1]==1 and i[2]==1: # TARG_EVENT and AD and error
                    # make errorred ones -ve as Mauro does.
                    line += ',{0}'.format(-i[4]/1000)
        line += '\n'

        return line

    # Compute ANOVA for this individual
    def anova(self):
        F, between_group_dof, within_group_dof, P = group_anova (self.nodi
        return (F, between_group_dof, within_group_dof, P)

    def reportmeans (self):
        print "Mean(SD): No distr: {0:.2f} ({1:.2f}) Sync: {2:.2f} ({3:.2f

    # Batch up all data in a form suitable for statsmodel's MultiCompariso
    # concatenating the ND, SD & AD data intoa single array, and making a
    def getMultiComparisonData (self):
        d = np.hstack((self.nodist_latencies[:,1],self.sync_latencies[:,1]

        nd_labels = np.ndarray(shape=(self.num_tgood(0),), dtype=object)
        nd_labels.fill('ND')

        sd_labels = np.ndarray(shape=(self.num_tgood(1),), dtype=object)
        sd_labels.fill('SD')
```

```
        ad_labels = np.ndarray(shape=(self.num_tgood(2),), dtype=object)
        ad_labels.fill('AD')

        l = np.hstack((nd_labels,sd_labels,ad_labels))

        # d is the data array, l is the label array.
        return (d, l)

    # Do a full set of graphs to show the normality of the data. Show QQ p
    # histograms of the distributions and results of Shapiro-Wilks tests i
    # Pass in the significance level for the S-W test.
    def shownormality (self, alpha):
        f, axarr = plt.subplots(3, 2)

        #ax1.set_title('QQ plots')
        fig1 = sm.qqplot(self.nodist_latencies[:,1], fit=True, line='45',a
        fig2 = sm.qqplot(self.sync_latencies[:,1], fit=True, line='45',ax=
        fig3 = sm.qqplot(self.async_latencies[:,1], fit=True, line='45', a
        axarr[0,0].set_title('QQ Plots')

        W, p = stats.shapiro (subsample(self.nodist_latencies[:,1], self.n
        isNormal = False
        if p > alpha:
            isNormal = True
        sw = 'ND. Mean/SD:{2:.2f}/{3:.2f} W={0:.2f}, p={1:.2f} (Normal:{4}
        axarr[0,1].hist(self.nodist_latencies[:,1], bins=20, label=sw)
        axarr[0,1].legend(prop={'size':9})
        axarr[0,1].set_title('Dist\'ns with Shapiro-Wilks stats')

        W, p = stats.shapiro (subsample(self.sync_latencies[:,1], self.num
        isNormal = False
        if p > alpha:
            isNormal = True
        sw = 'SD. Mean/SD:{2:.2f}/{3:.2f} W={0:.2f}, p={1:.2f} (Normal:{4}
        axarr[1,1].hist(self.sync_latencies[:,1], bins=20, label=sw)
        axarr[1,1].legend(prop={'size':9})

        W, p = stats.shapiro (subsample(self.async_latencies[:,1], self.nu
        isNormal = False
        if p > alpha:
            isNormal = True
        sw = 'AD. Mean/SD:{2:.2f}/{3:.2f} W={0:.2f}, p={1:.2f} (Normal:{4}
        axarr[2,1].hist(self.async_latencies[:,1], bins=20, label=sw)
        axarr[2,1].legend(prop={'size':9})

        # Fine-tune figure; make subplots close to each other and hide x t
        # all but bottom plot.
        f.subplots_adjust(hspace=0)
```

```
        plt.setp([a.get_xticklabels() for a in f.axes[:-1]], visible=False

        savename = 'images/' + self.subj_id + 'normplot.png'
        plt.savefig(savename)
        plt.show()

    # Apply Shapiro-Wilk test. Null hypothesis is that the data are normal
    # distributed. If p < alpha then null hypothesis must be rejected and
    # cannot be considered to be normally distributed.
    def shapiroWilk (self, condition, alpha):

        W = -1
        p = -1
        isNormal = False

        if condition == 0:
            W, p = stats.shapiro (subsample(self.nodist_latencies[:,1], se
        elif condition == 1:
            ss = subsample(self.sync_latencies[:,1], self.numSubsamples)
            W, p = stats.shapiro (ss)
        elif condition == 2:
            W, p = stats.shapiro (subsample(self.async_latencies[:,1], sel
        # else leave W,p,isNormal with default values

        if p > alpha:
            isNormal = True

        return W, p, isNormal

    # Do a Quantile-Quantile plot to compare against normal distribution
    def qqplot (self):
        f, (ax1, ax2, ax3) = plt.subplots(3, sharex=True, sharey=True)
        ax1.set_title('QQ plots',fontsize=18)
        fig1 = sm.qqplot(self.nodist_latencies[:,1], fit=True, line='45',a
        fig2 = sm.qqplot(self.sync_latencies[:,1], fit=True, line='45',ax=
        fig3 = sm.qqplot(self.async_latencies[:,1], fit=True, line='45', a
        # Fine-tune figure; make subplots close to each other and hide x t
        # all but bottom plot.
        f.subplots_adjust(hspace=0)
        plt.setp([a.get_xticklabels() for a in f.axes[:-1]], visible=False
        plt.show()
        return f

    def boxplot (self):
        data = [self.nodist_latencies[:,1], self.sync_latencies[:,1], self
        plt.figure()
        plt.boxplot(data, 0, 'gD')
        nodist_cond_x = np.ones(self.nodist_latencies[:,1].size)-0.1
```

```
        sync_cond_x = 2*np.ones(self.sync_latencies[:,1].size)-0.1
        async_cond_x = 3*np.ones(self.async_latencies[:,1].size)-0.1
        nodist_pts = plt.scatter(nodist_cond_x, self.nodist_latencies[:,1]
        sync_pts = plt.scatter(sync_cond_x, self.sync_latencies[:,1])
        async_pts = plt.scatter(async_cond_x, self.async_latencies[:,1])
        plt.xlabel('Condition 1:ND 2:S 3:AS',fontsize=18)
        plt.ylabel('Latency (ms)',fontsize=18)
        savename = 'images/' + self.subj_id + 'boxplot.png'
        plt.savefig(savename)
        plt.show()

    # A standard method for excluding outliers
    def excludeOutliers (self, show_excluded=0):
        # Exclude from latency data
        if show_excluded:
            print 'Excluding good movement outliers...'
        self.excludeOutliers_mad_based(3.5,show_excluded)
        # Exclude from error data:
        if show_excluded:
            print 'Excluding error outliers...'
        self.excludeErrOutliers(3.5,show_excluded)# also mad based

    # subroutine of excludeOutliers_mad_based()
    def excludeOutliers_mad_based_(self, latencies, alldata, thresh, show_
        nd_outliers = compute_mad_outliers (latencies,thresh)
        # nd_outliers is a large array of True/False.
        # print 'nd_outliers:',nd_outliers
        del_indices = [i for i,elem in enumerate(nd_outliers) if elem == T
        # del_indices are the indices of the True values in nd_outliers
        if not del_indices:
            return latencies,alldata

        # The event number is not the same as the index into latencies
        event_indices = latencies[[del_indices]][:,0]

        if show_excluded:
            values_to_delete = latencies[[del_indices]][:,1]
            print 'deleting the following events',event_indices,'which hav

        keepmask_bool = np.invert(np.in1d(latencies[:,0], event_indices, a
        latencies = latencies[keepmask_bool]
        # Remove same indices from self.alldata:
        keepmask_bool = np.invert(np.in1d(alldata[:,0], event_indices, ass
        alldata = alldata[keepmask_bool]
        return latencies,alldata

    # Exclude outliers from each latency set based on median absolute devi
    def excludeOutliers_mad_based(self, thresh=3.5, show_excluded=0):
```

```
        if show_excluded:
            print 'exclude nodist latencies for subject ',self.subj_id,'fi
        self.nodist_latencies,self.alldata_nd = self.excludeOutliers_mad_b
        if show_excluded:
            print 'exclude sync latencies for subject ',self.subj_id,'file
        self.sync_latencies,self.alldata_sd = self.excludeOutliers_mad_bas
        if show_excluded:
            print 'exclude async latencies for subject ',self.subj_id,'fil
        self.async_latencies,self.alldata_ad = self.excludeOutliers_mad_ba

    def excludeErrOutliers (self, num_sds, show_excluded):
        if show_excluded:
            print 'exclude nodist latencies for subject ',self.subj_id,'fi
        self.nodist_err_latencies,self.alldata_nd = self.excludeOutliers_m
        if show_excluded:
            print 'exclude sync latencies for subject ',self.subj_id,'file
        self.sync_err_latencies,self.alldata_sd = self.excludeOutliers_mac
        if show_excluded:
            print 'exclude async latencies for subject ',self.subj_id,'fil
        self.async_err_latencies,self.alldata_ad = self.excludeOutliers_ma

    def randomly_subsample_data (self, num_data):
        random.seed(19742016)
        while len(self.nodist_latencies[:,1]) > num_data:
            remove_this = random.randint (0,len(self.nodist_latencies[:,1]
            self.nodist_latencies = np.delete(self.nodist_latencies, remov
        while len(self.sync_latencies[:,1]) > num_data:
            remove_this = random.randint (0,len(self.sync_latencies[:,1])-
            self.sync_latencies = np.delete(self.sync_latencies, remove_th
        while len(self.async_latencies[:,1]) > num_data:
            remove_this = random.randint (0,len(self.async_latencies[:,1])
            self.async_latencies = np.delete(self.async_latencies, remove_

    def graph1(self):
        print 'Showing graph for ', ind.subj_id
        means = (self.nodist_mean(), self.sync_mean(), self.async_mean())
        stds = (self.nodist_std(), self.sync_std(), self.async_std())
        index = np.arange(3)
        opacity = 0.4
        error_config = {'ecolor': '0.3'}
        rects1 = plt.bar(index, means, 0.2,
                         alpha=opacity,
                         color='b',
                         yerr=stds,
                         error_kw=error_config,
                         label=ind.subj_id)
        # Now draw the points on a scatter graph
        nodist_cond_x = np.zeros(self.nodist_latencies[:,1].size)
```

```
        sync_cond_x = np.ones(self.sync_latencies[:,1].size)
        async_cond_x = 2*np.ones(self.async_latencies[:,1].size)
        nodist_pts = plt.scatter(nodist_cond_x, self.nodist_latencies)
        sync_pts = plt.scatter(sync_cond_x, self.sync_latencies)
        async_pts = plt.scatter(async_cond_x, self.async_latencies)
        plt.xlabel('Condition 0:ND 1:S 2:AS')
        plt.ylabel('Latency (ms)')
        plt.title(self.subj_id)
        plt.show()
        return

    # Compute the sum of the squared displacements from the mean for all t
    def sumofsquare_displacements_all_from_value(self, value):
        sos = self.sumofsquare_displacements_from_value(0,value) + self.su
        return sos

    # Compute the sum of the squared displacements from the mean for all t
    def sumofsquare_displacements_all(self):
        sos = self.sumofsquare_displacements(0) + self.sumofsquare_displac
        return sos

    # Compute the sum of the squared displacements from the mean for the g
    def sumofsquare_displacements(self, condition):
        if condition == 0:
            mn = self.nodist_mean()
            squares = np.power((self.nodist_latencies[:,1] - mn), 2)
        elif condition == 1:
            mn = self.sync_mean()
            squares = np.power((self.sync_latencies[:,1] - mn), 2)
        else: # condition 2
            mn = self.async_mean()
            squares = np.power((self.async_latencies[:,1] - mn), 2)
        sos = np.sum(squares)
        return sos

    # Compute the sum of the squared displacements from the mean for the g
    def sumofsquare_displacements_from_value(self, condition, value):
        if condition == 0:
            squares = np.power((self.nodist_latencies[:,1] - value), 2)
            # Verification of this method:
            #squares_alt = 0
            #for i in self.nodist_latencies:
            #    imv = i - value
            #    squares_alt += imv*imv
            #print 'nd sum of squares:',np.sum(squares),'squares_alt:',squ
        elif condition == 1:
            squares = np.power((self.sync_latencies[:,1] - value), 2)
        else: # condition 2
```

```
            squares = np.power((self.async_latencies[:,1] - value), 2)
        sos = np.sum(squares)
        return sos

    def alldata_for_condition(self, condition):
        if condition == 0:
            thedata = self.alldata_nd
        elif condition == 1:
            thedata = self.alldata_sd
        else: # condition 2
            thedata = self.alldata_ad
        return thedata

    def num_target_omissions(self, condition):
        thedata = self.alldata_for_condition(condition)
        # Select rows for which omit==1:
        omit_indices = [i for i,elem in enumerate(thedata[:,5]) if elem ==
        num_omit = 0
        if omit_indices:
            # From those rows, select those for which event type is target
            omit_target_indices = [i for i,elem in enumerate(thedata[omit_
            if omit_target_indices:
                num_omit = thedata[[omit_target_indices]][:,4].size

        return num_omit

    def num_target_non_omissions(self, condition):
        thedata = self.alldata_for_condition(condition)
        # Select rows for which omit==0:
        nomit_indices = [i for i,elem in enumerate(thedata[:,5]) if elem =
        num_nomit = 0
        if nomit_indices:
            # From those rows, select those for which event type is target
            nomit_target_indices = [i for i,elem in enumerate(thedata[nomi
            if nomit_target_indices:
                num_nomit = thedata[[nomit_target_indices]][:,4].size

        return num_nomit

    def num_distractor_events(self, condition):

        # In ND case, num_distractor_events == 0 by definition
        num = 0
        if condition == 0:
            return num

        # In SD case, num_distractor_events == num_target_events
        if condition == 1:
```

```
            return self.num_target_events(condition)

        thedata = self.alldata_for_condition(condition)
        distractor_indices = [i for i,elem in enumerate(thedata[:,1]) if e
        if distractor_indices:
            num = thedata[[distractor_indices]][:,4].size
        return num

    def num_target_events(self, condition):
        thedata = self.alldata_for_condition(condition)
        distractor_indices = [i for i,elem in enumerate(thedata[:,1]) if e
        num = 0
        if distractor_indices:
            num = thedata[[distractor_indices]][:,4].size
        return num

    def num_distractor_omissions(self, condition):
        thedata = self.alldata_for_condition(condition)

        # Select rows for which omit==1:
        omit_indices = [i for i,elem in enumerate(thedata[:,5]) if elem ==
        num_omit = 0
        if omit_indices:
            # From those rows, select those for which event type is distra
            omit_distractor_indices = [i for i,elem in enumerate(thedata[o
            if omit_distractor_indices:
                num_omit = thedata[[omit_distractor_indices]][:,4].size

        return num_omit

    def num_distractor_non_omissions(self, condition):
        thedata = self.alldata_for_condition(condition)

        # Select rows for which omit==0:
        nomit_indices = [i for i,elem in enumerate(thedata[:,5]) if elem =
        num_nomit = 0
        if nomit_indices:
            # From those rows, select those for which event type is distra
            nomit_distractor_indices = [i for i,elem in enumerate(thedata[
            if nomit_distractor_indices:
                num_nomit = thedata[[nomit_distractor_indices]][:,4].size

        return num_nomit

    # Movements following target events which are good and without error
    def num_tgood(self, condition):
        if condition == 0:
            if self.nodist_latencies.size<2:
```

```
                return 0
            return self.nodist_latencies[:,1].size
        elif condition == 1:
            if self.sync_latencies.size<2:
                return 0
            return self.sync_latencies[:,1].size
        else: # condition 2
            if self.async_latencies.size<2:
                return 0
            return self.async_latencies[:,1].size

    def num_tgood_all(self):
        n = self.num_tgood(0) + self.num_tgood(1) + self.num_tgood(2)
        return n

    # num_tgood_percent. Number of good movements as a percentage of numbe
    def num_tgood_percent(self, condition):
        if condition == 0:
            if self.nodist_latencies.size<2:
                return 0
            return 100*self.nodist_latencies[:,1].size/self.num_target_eve
        elif condition == 1:
            if self.sync_latencies.size<2:
                return 0
            return 100*self.sync_latencies[:,1].size/self.num_target_event
        else: # condition 2
            if self.async_latencies.size<2:
                return 0
            #prop_targets_non_omitted = self.num_target_non_omissions(cond
            #representative_num_distractors = prop_targets_non_omitted * s
            return 100*self.async_latencies[:,1].size/self.num_target_even

    # moveerrors are "errors for target movements"
    def num_tmoveerrors_all(self):
        n = self.num_tmoveerrors(0) + self.num_tmoveerrors(1) + self.num_t
        return n

    # Number of target movement errors
    def num_tmoveerrors(self, condition):

        #print 'num_tmoveerrors(',condition,') called'
        if condition == 0:
            #print 'nodist_err_latencies.size:',self.nodist_err_latencies.
            if self.nodist_err_latencies.size<2:
                #print 'Returning 0 for nodist_err_latencies:',self.nodist
                return 0
            elif self.nodist_err_latencies.size == 2:
                #print 'Returning 1 for nodist_err_latencies:',self.nodist
```

```
                return 1
            #print 'Returning [:,1].size for nodist_err_latencies'
            return self.nodist_err_latencies[:,1].size
        elif condition == 1:
            #print 'sync_err_latencies.size:',self.sync_err_latencies.size
            if self.sync_err_latencies.size<2:
                 return 0
            elif self.sync_err_latencies.size == 2:
                return 1
            return self.sync_err_latencies[:,1].size
        else: # condition 2
            #print 'async_err_latencies.size:',self.async_err_latencies.si
            if self.async_err_latencies.size<2:
                 return 0
            elif self.async_err_latencies.size == 2:
                return 1
            return self.async_err_latencies[:,1].size

    # Number of target movement errors as a percentage of number of target
    def num_tmoveerrors_percent(self, condition):
        if condition == 0:
            if self.nodist_err_latencies.size<2:
                 return 0
            return 100*self.nodist_err_latencies[:,1].size/self.num_target
        elif condition == 1:
            if self.sync_err_latencies.size<2:
                 return 0
            return 100*self.sync_err_latencies[:,1].size/self.num_target_r
        else: # condition 2
            if self.async_err_latencies.size<2:
                 return 0
            return 100*self.async_err_latencies[:,1].size/self.num_target_

    # dmoverrors are : Distractor move errors
    def num_dmoveerrors_all(self):
        n = self.num_dmoveerrors(0) + self.num_dmoveerrors(1) + self.num_c
        return n

    def num_dmoveerrors(self, condition):
        if condition == 0:
            if self.nodist_derr_latencies.size<2:
                 return 0
            return self.nodist_derr_latencies[:,1].size
        elif condition == 1:
            if self.sync_derr_latencies.size<2:
                 return 0
            return self.sync_derr_latencies[:,1].size
        else: # condition 2
```

```
        if self.async_derr_latencies.size<2:
            return 0
        return self.async_derr_latencies[:,1].size

# number of detected distractor errors as a percentage of the
# total number of distractor events
def num_dmoveerrors_percent(self, condition):
    if condition == 0:
        return 0 # 0 by definition
    elif condition == 1:
        if self.sync_derr_latencies.size<2:
            return 0
        return 100*self.sync_derr_latencies[:,1].size/self.num_distrac
    else: # condition 2
        if self.async_derr_latencies.size<2:
            return 0
        return 100*self.async_derr_latencies[:,1].size/self.num_distra

# Total number of movement errors (target and distractor) as a
# percentage of the number of target events not omitted
def num_moveerrors_per_target_percent(self, condition):
    moveerrors_percent = 0
    if condition == 0:
        # same as num_tmoveerrors_percent:
        moveerrors_percent = self.num_tmoveerrors_percent(condition)
    elif condition == 1:
        numerr = self.num_tmoveerrors(condition)+self.num_dmoveerrors
        moveerrors_percent = 100 * numerr / self.num_target_non_omissi
    else: # condition == 2
        numerr = self.num_tmoveerrors(condition)+self.num_dmoveerrors
        moveerrors_percent = 100 * numerr / self.num_target_non_omissi
    return moveerrors_percent

# as above, but not as a percentage
def num_moveerrors_per_target(self, condition):
    moveerrors_ = 0
    if condition == 0 or condition == 1:
        # same as num_tmoveerrors_ for the ND and SD cases
        moveerrors_ = self.num_tmoveerrors_percent(condition)/100
    else: # condition == 2
        numerr = self.num_tmoveerrors(condition)+self.num_dmoveerrors
        moveerrors_ = numerr / self.num_target_non_omissions(conditior
    return moveerrors_

# Total number of movement errors as a percentage of "the
# proportion of all distractor events matching the proportion of
# target events not omitted from latency measurement"
def num_moveerrors_per_distractor_percent(self, condition):
```

```
        moveerrors_percent = 0
        prop_target_events = self.num_target_non_omissions(condition)/self
        if condition == 0:
            moveerrors_percent = -1 # nan really
        elif condition == 1:
            # For the SD condition, target moveerrors == distractor moveer
            moveerrors_percent = self.num_moveerrors_per_target_percent(co
        else: # condition == 2
            numerr = self.num_tmoveerrors(condition)+self.num_dmoveerrors
            moveerrors_percent = 100 * numerr / self.num_distractor_events
        return moveerrors_percent

    # dgood : Distractor events without a move error
    def num_dgood_all(self):
        n = self.num_dgood(0) + self.num_dgood(1) + self.num_dgood(2)
        return n

    def num_dgood(self, condition):
        if condition == 0:
            if self.nodist_dgood_latencies.size<2:
                return 0
            return self.nodist_dgood_latencies[:,1].size
        elif condition == 1:
            if self.sync_dgood_latencies.size<2:
                return 0
            return self.sync_dgood_latencies[:,1].size
        else: # condition 2
            if self.async_dgood_latencies.size<2:
                return 0
            return self.async_dgood_latencies[:,1].size

    def num_dgood_percent(self, condition):
        if condition == 0:
            if self.nodist_dgood_latencies.size<2:
                return 0
            return 100*self.nodist_dgood_latencies[:,1].size/(self.num_tgo
        elif condition == 1:
            if self.sync_dgood_latencies.size<2:
                return 0
            return 100*self.sync_dgood_latencies[:,1].size/(self.num_tgood
        else: # condition 2
            if self.async_dgood_latencies.size<2:
                return 0
            return 100*self.async_dgood_latencies[:,1].size/(self.num_tgoo

    def nodist_mean(self):
        if self.nodist_latencies.size<2:
            return 0
```

```
        return self.nodist_latencies[:,1].mean()

    def sync_mean(self):
        if self.sync_latencies.size<2:
            return 0
        return self.sync_latencies[:,1].mean()

    def async_mean(self):
        if self.async_latencies.size<2:
            return 0
        return self.async_latencies[:,1].mean()

    def overall_mean(self):
        all_latencies = np.concatenate((self.nodist_latencies, self.sync_l
        return all_latencies[:,1].mean()

    def overall_std(self):
        all_latencies = np.concatenate((self.nodist_latencies, self.sync_l
        return all_latencies[:,1].std()

    def nodist_std(self):
        if self.nodist_latencies.size<2:
            return 0
        return self.nodist_latencies[:,1].std()

    def sync_std(self):
        if self.sync_latencies.size<2:
            return 0
        return self.sync_latencies[:,1].std()

    def async_std(self):
        if self.async_latencies.size<2:
            return 0
        return self.async_latencies[:,1].std()

    # mean/std accessors for the error latencies:
    def nodist_err_mean(self):
        if self.nodist_err_latencies.size<2:
            return 0
        return self.nodist_err_latencies.mean()

    def sync_err_mean(self):
        if self.sync_err_latencies.size<2:
            return 0
        return self.sync_err_latencies.mean()

    def async_err_mean(self):
        if self.async_err_latencies.size<2:
```

```
            return 0
        return self.async_err_latencies.mean()

    def overall_err_mean(self):
        all_err_latencies = np.concatenate((self.nodist_err_latencies, sel
        return all_err_latencies[:,1].mean()

    def overall_err_std(self):
        all_err_latencies = np.concatenate((self.nodist_err_latencies, sel
        return all_err_latencies[:,1].std()

    def nodist_err_std(self):
        if self.nodist_err_latencies.size<2:
            return 0
        return self.nodist_err_latencies.std()

    def sync_err_std(self):
        if self.sync_err_latencies.size<2:
            return 0
        return self.sync_err_latencies.std()

    def async_err_std(self):
        if self.async_err_latencies.size<2:
            return 0
        return self.async_err_latencies.std()

    # mean/std accessors for the distractor error latencies:
    def nodist_derr_mean(self):
        if self.nodist_derr_latencies.size<2:
            return 0
        return self.nodist_derr_latencies.mean()

    def sync_derr_mean(self):
        if self.sync_derr_latencies.size<2:
            return 0
        return self.sync_derr_latencies.mean()

    def async_derr_mean(self):
        if self.async_derr_latencies.size<2:
            return 0
        return self.async_derr_latencies.mean()

    def overall_derr_mean(self):
        all_derr_latencies = np.concatenate((self.nodist_derr_latencies, s
        return all_derr_latencies[:,1].mean()

    def overall_derr_std(self):
        all_derr_latencies = np.concatenate((self.nodist_derr_latencies, s
```

```
        return all_derr_latencies[:,1].std()

    def nodist_derr_std(self):
        if self.nodist_derr_latencies.size<2:
            return 0
        return self.nodist_derr_latencies.std()

    def sync_derr_std(self):
        if self.sync_derr_latencies.size<2:
            return 0
        return self.sync_derr_latencies.std()

    def async_derr_std(self):
        if self.async_derr_latencies.size<2:
            return 0
        return self.async_derr_latencies.std()

    # mean/std accessors for the distractor non-movement-error latencies:
    def nodist_dgood_mean(self):
        if self.nodist_dgood_latencies.size<2:
            return 0
        return self.nodist_dgood_latencies.mean()

    def sync_dgood_mean(self):
        if self.sync_dgood_latencies.size<2:
            return 0
        return self.sync_dgood_latencies.mean()

    def async_dgood_mean(self):
        if self.async_dgood_latencies.size<2:
            return 0
        return self.async_dgood_latencies.mean()

    def overall_dgood_mean(self):
        all_dgood_latencies = np.concatenate((self.nodist_dgood_latencies,
        return all_dgood_latencies[:,1].mean()

    def overall_dgood_std(self):
        all_dgood_latencies = np.concatenate((self.nodist_dgood_latencies,
        return all_dgood_latencies[:,1].std()

    def nodist_dgood_std(self):
        if self.nodist_dgood_latencies.size<2:
            return 0
        return self.nodist_dgood_latencies.std()

    def sync_dgood_std(self):
        if self.sync_dgood_latencies.size<2:
```

```
            return 0
        return self.sync_dgood_latencies.std()

    def async_dgood_std(self):
        if self.async_dgood_latencies.size<2:
            return 0
        return self.async_dgood_latencies.std()

    def report_movements(self,cond):
        if cond==0:
            str = '    ND'
        elif cond==1:
            str = '    Sync'
        else:
            print 'cond=',cond
            str = '    Async'

        print str,'non-error target movements',ind.num_tgood(cond),'-',10
        print str,'non-error distractor events',ind.num_dgood(cond),'-',1
        print str,'target movement errors:',ind.num_tmoveerrors(cond),'-',
        print str,'distractor movement errors:',ind.num_dmoveerrors(cond),
        print str,'total events:',ind.num_tgood(cond)+ind.num_tmoveerrors
        print ''

    def __str__(self):
        return "Data container for subject {0}".format(self.subj_id)

    # Output latency means for the three conditions as three lines for a 
    def outputDataForR(self):
        # R doesn't like 'NA' in a field - it reads it as "not
        # available" so substitute "NA_" for "NA" here.
        if self.subj_id == 'NA':
            self.subj_id = 'NA_';
        line = '{2},{1},0,ND,{0}\n'.format(self.subj_id,self.nodist_latenc
        line += '{2},{1},1,SD,{0}\n'.format(self.subj_id,self.sync_latenci
        line += '{2},{1},2,AD,{0}\n'.format(self.subj_id,self.async_latenc
        return line

    # Write out per-individual data for analysis in R into a file.
    def writeDataForR(self):
        dfname = 'IndDat' + self.subj_id + '.csv'

        theheader = "latency,condition_str\n"
        minsize = np.min([self.nodist_latencies[:,1].size,self.sync_latenc
        _nd = subsample(self.nodist_latencies[:,1], minsize)
        _sd = subsample(self.sync_latencies[:,1], minsize)
        _ad = subsample(self.async_latencies[:,1], minsize)
```

```
        f = open(dfname, 'w')
        f.write(theheader)
        # allaltlines is the latency trials all together
        allaltlines = ''
        for i in range(1,minsize):
            line = "{0},ND\n{1},SD\n{2},AD\n".format(_nd[i],_sd[i],_ad[i])
            altline = "{3},{0},ND\n{3},{1},SD\n{3},{2},AD\n".format(_nd[i]
            allaltlines += altline
            f.write(line)
        f.close()
        return allaltlines

    def writeNoDistDataForR(self):
        all_lines = ''
        if self.subj_id == 'NA':
            self.subj_id = 'NA_';
        theheader = "condition_str,subj_id,num,type,error,correctmove,late
        dfname = 'NoDistDat' + self.subj_id + '.csv'
        f = open(dfname, 'w')
        f.write(theheader)
        minsize = self.alldata_nd[:,1].size
        for i in range(1,minsize):
            line = "ND,{0},{1},{2},{3},{4},{5},{6},{7}\n".format(
                self.subj_id,
                self.alldata_nd[i,0],
                self.alldata_nd[i,1],
                self.alldata_nd[i,2],
                self.alldata_nd[i,3],
                self.alldata_nd[i,4],
                self.alldata_nd[i,5],#omit
                self.alldata_nd[i,8]
            )
            f.write(line)
            all_lines += line
        f.close()
        return all_lines

    def writeSyncDataForR(self):
        all_lines = ''
        if self.subj_id == 'NA':
            self.subj_id = 'NA_';
        theheader = "condition_str,subj_id,num,type,error,correctmove,late
        dfname = 'SyncDat' + self.subj_id + '.csv'
        f = open(dfname, 'w')
        f.write(theheader)
        minsize = self.alldata_sd[:,1].size
        for i in range(1,minsize):
            line = "SD,{0},{1},{2},{3},{4},{5},{6},{7}\n".format(
```

```
                self.subj_id,
                self.alldata_sd[i,0],#evnum
                self.alldata_sd[i,1],#evtype
                self.alldata_sd[i,2],#everror
                self.alldata_sd[i,3],#correct_move
                self.alldata_sd[i,4],#latency
                self.alldata_sd[i,5],#omit
                self.alldata_sd[i,8]#direction
            )
            f.write(line)
            all_lines += line
        f.close()
        return all_lines


    # Write out asynchronous data including both distractors and targets a
    def writeAsyncDataForR(self):
        # We have alldata_ad which contains columns: evnum,evtype,everror,
        # The simplest approach is probably to simply write this out, alon
        # manipulations in R.
        all_lines = ''
        if self.subj_id == 'NA':
            self.subj_id = 'NA_';
        theheader = "condition_str,subj_id,num,type,error,correctmove,late
        dfname = 'AsyncDat' + self.subj_id + '.csv'
        f = open(dfname, 'w')
        f.write(theheader)
        minsize = self.alldata_ad[:,1].size
        for i in range(1,minsize):
            line = "AD,{0},{1},{2},{3},{4},{5},{6},{7},{8},{9},{10},{11},{
                self.subj_id,
                self.alldata_ad[i,0],#evnum
                self.alldata_ad[i,1],#evtype
                self.alldata_ad[i,2],#everror
                self.alldata_ad[i,3],#correct_move
                self.alldata_ad[i,4],#latency
                self.alldata_ad[i,5],#omit
                self.alldata_ad[i,6],#oreas
                self.alldata_ad[i,7],#time_since_last
                self.alldata_ad[i,8],#direction
                self.alldata_ad[i,9],  #destination
                self.alldata_ad[i,10], #last_distractor_offset
                self.alldata_ad[i,11]  #time_since_last_distractor
            )
            f.write(line)
            all_lines += line
        f.close()
        return all_lines
```

```
    # Output for two factor anova analysis in SPSS
    def outputDataForSPSS(self):
        for lat in self.nodist_latencies[:,1]:
            print '{0},ND,{1}'.format(self.subj_id,lat)
        for lat in self.sync_latencies[:,1]:
            print '{0},SD,{1}'.format(self.subj_id,lat)
        for lat in self.async_latencies[:,1]:
            print '{0},AD,{1}'.format(self.subj_id,lat)

    # Output for univariate or multivariate repeated measures analysis
    def outputDataForSPSS_MV(self):
        print '{0},ND,{1},{2}'.format(self.subj_id,self.nodist_latencies[:
        print '{0},SD,{1},{2}'.format(self.subj_id,self.sync_latencies[:,1
        print '{0},AD,{1},{2}'.format(self.subj_id,self.async_latencies[:,

    # Output for repeated measures analysis of latency
    def outputDataForSPSS_RepMeasLat(self):
        print '{0},{1},{2},{3}'.format(self.subj_id,self.nodist_latencies.

    # Output for repeated measures analysis of error rate
    def outputDataForSPSS_RepMeasError(self):
        print '{0},{1},{2},{3}'.format(self.subj_id,self.n_errors_per_targ

### END CLASS individual ###

# This function sets up a list of individuals and populates the latencies.
def readIndividuals():
    individuals = dict()
    idnum_counter = 1
    # Extract the data from the raw format and collate it into individual
    # data containers, one per subject.
    for fname in zip(*fnames):
        # I'll use the subject ID as a key into output data structures
        subj_id = getfnameid(fname[0][0])

        # condition index is for the no distractor/sync distractor/async c
        condition_index = getcondition(fname[1][0,0][36][0])

        # Need ONE individual object for each subj_id.
        if subj_id not in individuals:
            individuals[subj_id] = individual(subj_id) # or if imported in
            individuals[subj_id].idnum = idnum_counter
            idnum_counter += 1

        # latencies for all events
        #
        # All the fname[n] contain:
```

```
# ev.number, ev.type, ev.error, ev.correct_move, ev.latency. Then
#
# fname[2] additionally has: ev.omit and omit_reason (as a
# numeric code) cols. So can obtain a value for "proportion of
# targets omitted".
#
#if individuals[subj_id].alldata.size < 2:
#    individuals[subj_id].alldata = np.append(fname[2], condition_
#else:
#    newdata = np.append(fname[2], condition_index*np.ones(fname[2
#    individuals[subj_id].alldata = np.concatenate((individuals[su

# latencies for non-movement-error target events
tnoerr_latencies = fname[4] # R.latency_noerror_target - Use table

# latencies for non-movement-error distractor events
dnoerr_latencies = fname[5]

# err_latencies = fname[6] # ALL error events, distractor and targ

# latencies for target movement error events
terr_latencies = fname[7] # R.latency_error_target

# latencies for distractor movement error events
derr_latencies = fname[8] # R.latency_error_distractor

#omitted = fname[1] # omitted events?

if condition_index == 0:
    individuals[subj_id].alldata_nd = fname[2] # contains dirn in
    #print 'alldata_nd has shape', fname[2].shape
    individuals[subj_id].filename_nd = fname[0][0]

    #print err_latencies
    individuals[subj_id].nodist_latencies = tnoerr_latencies[:,[0,
    individuals[subj_id].nodist_latencies_rank = stats.rankdata(in
    if terr_latencies.size > 0:
        individuals[subj_id].nodist_err_latencies = terr_latencies
        individuals[subj_id].nodist_err_latencies_rank = stats.ran
    # We'll have an error rate for nodist - it should be very low.
    nerrs = 0
    ntargets = 0
    for d in individuals[subj_id].alldata_nd:
        ntargets += 1 # "ndistractors" is really "ntargets" for th
        if d[2] > 0.0:
            nerrs += 1
    individuals[subj_id].n_errors_per_target_nd = (float(nerrs) /
```

```
elif condition_index == 1:
    individuals[subj_id].alldata_sd = fname[2]
    individuals[subj_id].filename_sd = fname[0][0]
    #print latencies
    individuals[subj_id].sync_latencies = tnoerr_latencies[:,[0,4]
    individuals[subj_id].sync_latencies_rank = stats.rankdata(indi
    if terr_latencies.size > 0:
        individuals[subj_id].sync_err_latencies = terr_latencies[:
        individuals[subj_id].sync_err_latencies_rank = stats.rankd
    # For sync, also read errors
    nerrs = 0
    ndistractors = 0
    #if subj_id == 'JS':
    #    print 'Sync'
    for d in individuals[subj_id].alldata_sd:
        #if subj_id == 'JS' and d[2] > 0.0:
        #    print d
        ndistractors += 1
        if d[2] > 0.0:
            nerrs += 1
    individuals[subj_id].n_errors_per_distractor_sync = (float(ner

elif condition_index == 2:
    individuals[subj_id].alldata_ad = fname[2]
    individuals[subj_id].filename_ad = fname[0][0]
    individuals[subj_id].async_latencies = tnoerr_latencies[:,[0,4
    individuals[subj_id].async_latencies_rank = stats.rankdata(ind
    #test[test[:, 1] == 4]
    if terr_latencies.size > 0:
        individuals[subj_id].async_err_latencies = terr_latencies[
        individuals[subj_id].async_err_latencies_rank = stats.rank
    if derr_latencies.size > 0:
        individuals[subj_id].async_derr_latencies = derr_latencies
        individuals[subj_id].async_derr_latencies_rank = stats.ran

    individuals[subj_id].async_tnoerr_timesince = tnoerr_latencies
    individuals[subj_id].async_derr_timesince = derr_latencies[:,[
    # plus derr_latencies[:,[]]

    # For async, also read errors
    nerrs = 0
    ndistractors = 0
    #if subj_id == 'JS':
    #    print 'Async'
    for d in individuals[subj_id].alldata_ad:
        #if subj_id == 'JS' and d[2] > 0.0:
        #    print d
        if d[2] > 0.0: # Count all errors for async
```

```
                        nerrs += 1
                    if d[1] < 1.0:
                        ndistractors += 1
                #if subj_id == 'JS':
                #    print 'Num movement errors:',nerrs,'Num distractor events
                individuals[subj_id].n_errors_per_distractor_async = (float(ne
                individuals[subj_id].n_async_distractors = ndistractors

        return individuals

    def equaliseReplicates(individuals):
        # Find smallest number of cell replications and reduce all cells to th
        smallest_n = 1000000
        for i in individuals:
            individuals[i].excludeOutliers()
            if individuals[i].num_tgood(0) < smallest_n:
                smallest_n = individuals[i].num_tgood(0)
            if individuals[i].num_tgood(1) < smallest_n:
                smallest_n = individuals[i].num_tgood(1)
            if individuals[i].num_tgood(2) < smallest_n:
                smallest_n = individuals[i].num_tgood(2)

        # Randomly select smallest_n data from each condition in each individu
        for i in individuals:
            individuals[i].randomly_subsample_data(smallest_n)

        return individuals
```

```
/usr/lib/python2.7/dist-packages/matplotlib/font_manager.py:273: UserWarning: Matpl
  warnings.warn('Matplotlib is building the font cache using fc-list. This may take
```

1.4 Numbers of errors and omitted events

This is the definitive example of how to find out the number of target & distractor errors and the number of omitted events.

```
In [11]: for i,ind in readIndividuals().iteritems():
             print 'Subject ID: ', ind.subj_id

             print 'ND'
             print 'num target events:', ind.num_target_events(0)
             print 'num distractor events:', ind.num_distractor_events(0)
             print 'num_target_omissions:',ind.num_target_omissions(0)
             print 'num_tgood:',ind.num_tgood(0)
             print 'num_tmoveerrors',ind.num_tmoveerrors(0)
             print 'num_target_non_omissions (should equal num_tgood + num_tmoveerr
             print 'tmoveerrors percent', ind.num_tmoveerrors_percent(0)
```

```
print 'dmoveerrors percent', ind.num_dmoveerrors_percent(0)
print 'moveerrors per target percent', ind.num_moveerrors_per_target_p
print 'moveerrors per distractor percent', ind.num_moveerrors_per_dist
print 'num_target_non_omissions',ind.num_target_non_omissions(0)
print 'num_target_omissions',ind.num_target_omissions(0)
print 'num_distractor_non_omissions',ind.num_distractor_non_omissions
print 'num_distractor_omissions',ind.num_distractor_omissions(0)


print 'SD'
print 'num target events:', ind.num_target_events(1)
print 'num distractor events:', ind.num_distractor_events(1)
print 'num_target_omissions:',ind.num_target_omissions(1)
print 'num_tgood:',ind.num_tgood(1)
print 'num_tmoveerrors',ind.num_tmoveerrors(1)
print 'num_target_non_omissions (should equal num_tgood + num_tmoveerr
##
print 'tmoveerrors percent', ind.num_tmoveerrors_percent(1)
print 'dmoveerrors percent', ind.num_dmoveerrors_percent(1)
print 'moveerrors per target percent', ind.num_moveerrors_per_target_p
print 'moveerrors per distractor percent', ind.num_moveerrors_per_dist
print 'num_target_non_omissions',ind.num_target_non_omissions(1)
print 'num_target_omissions',ind.num_target_omissions(1)
print 'num_distractor_omissions:',ind.num_distractor_omissions(1)
print 'num_distractor_non_omissions:',ind.num_distractor_non_omissions

print 'AD'
print 'num target events:', ind.num_target_events(2)
print 'num distractor events:', ind.num_distractor_events(2)
print 'num_target_omissions:',ind.num_target_omissions(2)
print 'num_tgood:',ind.num_tgood(2)
print 'num_tmoveerrors',ind.num_tmoveerrors(2)
print 'num_target_non_omissions (num_tgood+tmoveerrors):',ind.num_targ
print 'num_dgood:',ind.num_dgood(2)
print 'num_dmoveerrors',ind.num_dmoveerrors(2)
##
print 'tmoveerrors percent', ind.num_tmoveerrors_percent(2)
print 'dmoveerrors percent', ind.num_dmoveerrors_percent(2)
print 'moveerrors per target percent', ind.num_moveerrors_per_target_p
print 'moveerrors per distractor percent', ind.num_moveerrors_per_dist
print 'num_target_non_omissions',ind.num_target_non_omissions(2)
print 'num_target_omissions',ind.num_target_omissions(2)
print 'num_distractor_omissions:',ind.num_distractor_omissions(2)
print 'num_distractor_non_omissions:',ind.num_distractor_non_omissions

break # So that we only output the information for one individual
```

```
Subject ID:  SB2
ND
num target events: 68
num distractor events: 0
num_target_omissions: 31
num_tgood: 35
num_tmoveerrors 2
num_target_non_omissions (should equal num_tgood + num_tmoveerrors): 37
tmoveerrors percent 5.40540540541
dmoveerrors percent 0
moveerrors per target percent 5.40540540541
moveerrors per distractor percent -1
num_target_non_omissions 37
num_target_omissions 31
num_distractor_non_omissions 0
num_distractor_omissions 0
SD
num target events: 66
num distractor events: 66
num_target_omissions: 23
num_tgood: 32
num_tmoveerrors 11
num_target_non_omissions (should equal num_tgood + num_tmoveerrors): 43
tmoveerrors percent 25.5813953488
dmoveerrors percent 0
moveerrors per target percent 25.5813953488
moveerrors per distractor percent 25.5813953488
num_target_non_omissions 43
num_target_omissions 23
num_distractor_omissions: 0
num_distractor_non_omissions: 0
AD
num target events: 70
num distractor events: 118
num_target_omissions: 33
num_tgood: 33
num_tmoveerrors 4
num_target_non_omissions (num_tgood+tmoveerrors): 37
num_dgood: 0
num_dmoveerrors 4
tmoveerrors percent 10.8108108108
dmoveerrors percent 3.38983050847
moveerrors per target percent 21.6216216216
moveerrors per distractor percent 3.58353510896
num_target_non_omissions 37
num_target_omissions 33
num_distractor_omissions: 89
num_distractor_non_omissions: 29
```

1.5 Breakdown of omit reasons

I want to count up the number of different omit reasons for various event types. To do this, need to add any of the new omit reasons in lt_analyse_latency.m.

In the octave script "omit" means "omit from latency measurements". That means that an omitted distractor movement may be a correct, error-free event; it's just that because the user did not move in response to the distractor, no latency measurement could be measured.

So, when counting omit reasons to see how many events were omitted and how many considered for *errors*, if the reason is "16 - Movement occurs beyond next target", that's no error and it goes in the "kept" pile.

```
In [12]: # Count omit reasons...
         from __future__ import division
         import numpy as np
         DIST_EVENT=0
         TARG_EVENT=1

         def omit_reason_summary (omit_reason_count_arr):
             iterator = 0 # into omit_reason_count_arr
             latmeas_count = 0    # "latency measurement count"
             nolatmeas_count = 0 # "omitted from latency measurement count"
             omit_count = 0       # "omitted from error counting"
             nomit_count = 0      # "included in error counting"
             for thecount in omit_reason_count_arr:
                 if thecount > 0:
                     print 'omit reason',omit_reason(iterator),'count:',thecount

                 # How many events considered for latency measurements?
                 if iterator == 0:
                     latmeas_count += thecount
                 else:
                     nolatmeas_count += thecount

                 # How many considered for error counting?
                 if iterator == 0 or iterator == 16:
                     # Note - 0 means no omission at all. 16 is special - it is
                     # where the distractor event was omitted because it didn't
                     # distract the subject, so it should be counted as no
                     # omission.
                     nomit_count += thecount
                 else:
                     omit_count += thecount

                 iterator += 1
             print nomit_count,'kept for error-counting,',omit_count,'omitted from
             print latmeas_count,'kept for latency measurements,',nolatmeas_count,'
```

```
    print 'There were ', nomit_count+omit_count, 'events in total.'
    print ''
    return nomit_count+omit_count

# To count up total events:
targ_event_count = 0
dist_event_count = 0

omit_reason_count_arr = np.zeros(21).astype(int)
for i,ind in readIndividuals().iteritems():
    ador = ind.ad_omit_reasons(TARG_EVENT)
    omit_reason_count_arr += ador
    #print 'Omit reason summary for Async condition, target events, INDIVI
    #omit_reason_summary (ador)
print 'Omit reason summary for Async condition, target events'
print '--------------------------------------------------------'
targ_event_count += omit_reason_summary (omit_reason_count_arr)

omit_reason_count_arr = np.zeros(21).astype(int)
for i,ind in readIndividuals().iteritems():
    ador = ind.ad_omit_reasons(DIST_EVENT)
    omit_reason_count_arr += ador
    #print 'Omit reason summary for Async condition, distractor events, IN
    #omit_reason_summary (ador)
print 'Omit reason summary for Async condition, distractor events'
print '--------------------------------------------------------'
dist_event_count += omit_reason_summary (omit_reason_count_arr)

omit_reason_count_arr = np.zeros(21).astype(int)
for i,ind in readIndividuals().iteritems():
    ador = ind.sd_omit_reasons(TARG_EVENT)
    omit_reason_count_arr += ador
print 'Omit reason summary for Sync condition, target events'
print '--------------------------------------------------------'
targ_event_count += omit_reason_summary (omit_reason_count_arr)

omit_reason_count_arr = np.zeros(21).astype(int)
for i,ind in readIndividuals().iteritems():
    ador = ind.nd_omit_reasons(TARG_EVENT)
    omit_reason_count_arr += ador
    #print 'Omit reason summary for ND condition, target events, INDIVIDUA
    #omit_reason_summary (ador)
    #print ador
    #print omit_reason_count_arr
print 'Omit reason summary for ND condition, target events'
print '--------------------------------------------------------'
targ_event_count += omit_reason_summary (omit_reason_count_arr)
```

```
        print ''
        print 'Total target events:',targ_event_count,'Total distractor events:',c
        print 'Grand total events:',targ_event_count+dist_event_count
```

```
Omit reason summary for Async condition, target events
-------------------------------------------------------
omit reason 0 not omitted count: 2313
omit reason 3 target posn change less than min. jump size count: 91
omit reason 7 Too fast (targ) count: 62
omit reason 9 No movement detected count: 8
omit reason 11 Stable stylus period too short count: 351
omit reason 12 Drift too great during stable stylus period count: 6
omit reason 13 Drift too great during stable period (avg) count: 661
omit reason 17 Subject was distracted by closely previous distractor count: 4
omit reason 18 Incorrect move was recorded in previous distractor event count: 7
2313 kept for error-counting, 1190 omitted from error-counting
2313 kept for latency measurements, 1190 couldnt provide latency measurements
There were  3503 events in total.

Omit reason summary for Async condition, distractor events
-------------------------------------------------------
omit reason 0 not omitted count: 1692
omit reason 3 target posn change less than min. jump size count: 98
omit reason 4 Stable position later than event onset count: 123
omit reason 8 Too fast (distractor) count: 20
omit reason 11 Stable stylus period too short count: 1743
omit reason 12 Drift too great during stable stylus period count: 18
omit reason 13 Drift too great during stable period (avg) count: 957
omit reason 15 Stylus didn't move away from target count: 1326
omit reason 16 Movement occurs beyond next target count: 896
omit reason 19 This distractor event did not distract the stylus movement count: 64
omit reason 20 Recorded this stylus movement as a distraction towards the next dist
2588 kept for error-counting, 4353 omitted from error-counting
1692 kept for latency measurements, 5249 couldnt provide latency measurements
There were  6941 events in total.

Omit reason summary for Sync condition, target events
-------------------------------------------------------
omit reason 0 not omitted count: 2623
omit reason 3 target posn change less than min. jump size count: 119
omit reason 7 Too fast (targ) count: 32
omit reason 9 No movement detected count: 8
omit reason 11 Stable stylus period too short count: 214
omit reason 12 Drift too great during stable stylus period count: 7
omit reason 13 Drift too great during stable period (avg) count: 564
2623 kept for error-counting, 944 omitted from error-counting
2623 kept for latency measurements, 944 couldnt provide latency measurements
There were  3567 events in total.
```

```
Omit reason summary for ND condition, target events
-------------------------------------------------------
omit reason 0 not omitted count: 2684
omit reason 3 target posn change less than min. jump size count: 102
omit reason 7 Too fast (targ) count: 46
omit reason 9 No movement detected count: 5
omit reason 11 Stable stylus period too short count: 166
omit reason 12 Drift too great during stable stylus period count: 8
omit reason 13 Drift too great during stable period (avg) count: 595
2684 kept for error-counting, 922 omitted from error-counting
2684 kept for latency measurements, 922 couldnt provide latency measurements
There were  3606 events in total.


Total target events: 10676 Total distractor events: 6941
Grand total events: 17617
```

1.6 Show which events are outliers

This code runs through each individual showing which events are deleted from each dataset by passing 1 to the individuals.excludeOutliers() method.

```
In [13]: print 'Showing latency outlier event numbers and values'
         print '------------------------------------------------'
         individuals = readIndividuals()
         show_excluded = 0
         if show_excluded:
             for i,ind in individuals.iteritems():
                 ind.excludeOutliers(show_excluded)
         else:
             print '[Set show_excluded = 1 to see the (rather verbose) output of th
```

```
Showing latency outlier event numbers and values
------------------------------------------------
[Set show_excluded = 1 to see the (rather verbose) output of this block.]
```

1.7 Mean number of latency-to-first-movement values obtained by Octave pre-processing

Mean numbers of trials overall and for the 3 different conditions. Note that these are the mean numbers of trials which the Octave pre-processing script was able to turn into latency-to-first-movement values, rather than the mean number of target events, which is determined separately in a previous block called **Count number of target events**.

```
In [14]: from __future__ import division
         import numpy as np
```

```python
num_trials = []
nd_num_trials = []
sd_num_trials = []
ad_num_trials = []
min_nd_num_trials = 1000
min_sd_num_trials = 1000
min_ad_num_trials = 1000
min_nd_num_trials_idx = ''
min_sd_num_trials_idx = ''
min_ad_num_trials_idx = ''
max_nd_num_trials = 0
max_sd_num_trials = 0
max_ad_num_trials = 0
max_nd_num_trials_idx = ''
max_sd_num_trials_idx = ''
max_ad_num_trials_idx = ''
ind_ids = []
nd_percent = []
sd_percent = []
ad_percent = []

for i,ind in readIndividuals().iteritems():
    ind_ids.append(ind.subj_id)
    #print 'nodist_latencies.size:',ind.nodist_latencies.size
    #print 'nodist_latencies[0,:].size:',ind.nodist_latencies[0,:].size
    #print 'nodist_latencies[:,0].size:',ind.nodist_latencies[:,0].size #
    nd_num_trials = np.append(nd_num_trials, ind.nodist_latencies[:,0].siz
    sd_num_trials = np.append(sd_num_trials, ind.sync_latencies[:,0].size)
    ad_num_trials = np.append(ad_num_trials, ind.async_latencies[:,0].size
    num_trials = np.append(num_trials, ind.nodist_latencies[:,0].size)
    num_trials = np.append(num_trials, ind.sync_latencies[:,0].size)
    num_trials = np.append(num_trials, ind.async_latencies[:,0].size)
    nd_percent = np.append(nd_percent, ind.nodist_latencies[:,0].size/alle
    sd_percent = np.append(sd_percent, ind.sync_latencies[:,0].size/alleve
    ad_percent = np.append(ad_percent, ind.async_latencies[:,0].size/allev

    #print 'allevents[ind.subj_id+"nd"] is ', allevents[ind.subj_id+"nd"],
    #print 'allevents[ind.subj_id+"sd"] is ', allevents[ind.subj_id+"sd"],
    # AD is different. allevents[ind.subj_id+"ad"] contains the number of
    #print 'allevents[ind.subj_id+"ad"] is ', allevents[ind.subj_id+"ad"],

overall_mean_num_trials = num_trials.mean()
nd_mean_num_trials = nd_num_trials.mean()
sd_mean_num_trials = sd_num_trials.mean()
ad_mean_num_trials = ad_num_trials.mean()

print 'Mean numbers of trials:'
```

```
        print 'overall:', overall_mean_num_trials,'(',num_trials.std(),')','ND:',r
        print '\n'
        print 'Absolute statistics'
        print 'Mins: ND:',nd_num_trials.min(),'(at idx ',nd_num_trials.argmin(),',
        print '      SD:',sd_num_trials.min(),'(at idx ',sd_num_trials.argmin(),',
        print '      AD:',ad_num_trials.min(),'(at idx ',ad_num_trials.argmin(),',

        print 'Maxs: ND:',nd_num_trials.max(),'(at idx ',nd_num_trials.argmax(),',
        print '      SD:',sd_num_trials.max(),'(at idx ',sd_num_trials.argmax(),',
        print '      AD:',ad_num_trials.max(),'(at idx ',ad_num_trials.argmax(),',

        print '\nPercentage statistics:'
        print 'ND Percent min:',nd_percent.min(),"%","max:",nd_percent.max(),"%",'
        print 'SD Percent min:',sd_percent.min(),"%","max:",sd_percent.max(),"%",'
        print 'AD Percent (of targ events) min:',ad_percent.min(),"%","max:",ad_pe
```

```
Mean numbers of trials:
overall: 43.5878787879 ( 7.92081100744 ) ND: 48.0545454545 SD: 41.7818181818 AD: 4[illegible]


Absolute statistics
Mins: ND: 33.0 (at idx  22 , subj TM ) targevents: 57.0
      SD: 28.0 (at idx  44 , subj CM ) targevents: 60.0
      AD: 22.0 (at idx  46 , subj SS ) targevents: 72.0
Maxs: ND: 64.0 (at idx  43 , subj SY ) targevents: 73.0
      SD: 55.0 (at idx  12 , subj RQ ) targevents: 68.0
      AD: 56.0 (at idx  3 , subj CH ) targevents: 60.0

Percentage statistics:
ND Percent min: 50.0 % max: 90.0 % mean/sd 73.4327724852 9.13027390823
SD Percent min: 46.1538461538 % max: 88.0952380952 % mean/sd 64.7803976178 10.5121[illegible]
AD Percent (of targ events) min: 30.5555555556 % max: 97.5609756098 % mean/sd 65.0[illegible]
```

1.7.1 Summary of the above

The block above gives (for each condition) the minimum and maximum percentages of target events for which a latency-to-first-movement value was found by the Octave preprocessing scripts. Also given is the mean percentage success rate and associated SD and the minimum absolute number of latencies found.

1.8 Repeated measures ANOVA

If we collapse the replication of latency measurements into a mean latency for each condition/individual combination, then it's possible to perform a repeated measures ANOVA analysis. This was the original design of the experiment and is the primary analysis reported in the paper, however there an ANOVA on ranked data is reported as the Shapiro Wilks tests on normality of the mean latencies fails.

The QQ plot from this code block is reported in the paper.

```
In [15]: from __future__ import division
         # This repmeasures_anova works on the mean latency for each individual.
         # This is effectively a "two factor anova without replication"
         import numpy as np

         def repmeasures_anova(individuals):

             # A one-way repeated measures anova is computed like a "two-factor anc
             # for a random block design. That means sums of squares are computed i
             # This example follows McKillup Section 15.5 (p216).

             # Containers to fill
             nodist_latencies = [];
             sync_latencies = [];
             async_latencies = [];

             num_individuals = len(individuals)
             num_conditions = 3

             # Zeroth, exclude outliers (assumed already to have been done before t

             # In this loop extract the latencies into external containers:
             for i,ind in individuals.iteritems():
                 # NB: Assume here that any outlier excluding has ALREADY been carr
                 nodist_latencies = np.concatenate((nodist_latencies, [ind.nodist_m
                 sync_latencies = np.concatenate((sync_latencies, [ind.sync_mean()]
                 async_latencies = np.concatenate((async_latencies, [ind.async_mean

             # Compute grand mean
             all_latencies = np.concatenate((nodist_latencies, sync_latencies, asyn
             grand_mean = all_latencies.mean()
             print 'Grand mean:',grand_mean

             # Displacement due to ALL sources of variation in the experiment. Fact
             # total_variance. This is displacement from the grand mean.
             total_sos = 0
             total_dof = -1 # dof is number of replicates -1
             for i in individuals:
                 ind = individuals[i]
                 total_sos += np.power(grand_mean - ind.nodist_mean(), 2)
                 total_sos += np.power(grand_mean - ind.sync_mean(), 2)
                 total_sos += np.power(grand_mean - ind.async_mean(), 2)

             total_dof = (num_conditions*num_individuals)-1

             # So here's the total variance:
             total_variance = total_sos/total_dof
             print 'total sos:',total_sos,'total dof:',total_dof,'total variance:',
```

```
# Could also get total sos with:
# total_variance = all_latencies.var(ddof=1) # but it doesn't work as

# Now consider the data in relation to each of the two factors (indivi

# Compute condition_plus_error_sos (Factor A - equiv to Temperature in
nd_treatment_mean = 0
sd_treatment_mean = 0
ad_treatment_mean = 0

for i,ind in individuals.iteritems():
    nd_treatment_mean += ind.nodist_mean()
    sd_treatment_mean += ind.sync_mean()
    ad_treatment_mean += ind.async_mean()

nd_treatment_mean = nd_treatment_mean / num_individuals
sd_treatment_mean = sd_treatment_mean / num_individuals
ad_treatment_mean = ad_treatment_mean / num_individuals

# Now we have treatment means for each condition.

condition_sos = num_individuals*(np.power(nd_treatment_mean - grand_me
condition_dof = num_conditions-1 # 3 conditions - 1 = 2

condition_variance = condition_sos / condition_dof

# Compute individual_plus_error_sos (p178).
individual_sos = 0
for i,ind in individuals.iteritems():
    ind_mean = (ind.nodist_mean() + ind.sync_mean() + ind.async_mean()
    individual_sos += num_conditions*np.power(ind_mean-grand_mean,2)
individual_dof = (num_individuals-1)

individual_variance = individual_sos / individual_dof

# remainder variance computed by subtraction
##remainder_variance = total_variance - condition_variance - individua
remainder_sos = total_sos - condition_sos - individual_sos
print 'total_sos:', total_sos
print 'condition_sos:', condition_sos
print 'individual_sos:', individual_sos
print 'remainder_sos:', remainder_sos
remainder_dof = total_dof - condition_dof - individual_dof
# This is the best estimate for error in a two factor ANOVA without re
remainder_variance = remainder_sos/remainder_dof
print 'remainder_variance:',remainder_variance,'remainder_sos:',remain

print 'total_sos:',total_sos,'condition_sos:',condition_sos,'indiv_sos
```

```
    # Now compute the F ratios
    F_cond = condition_variance / remainder_variance # fixed. condition +
    F_ind = individual_variance / remainder_variance # random. individual

    # Lastly, what's the probability for this?
    P_cond = 1-special.fdtr(condition_dof,remainder_dof,F_cond)
    P_ind = 1-special.fdtr(individual_dof,remainder_dof,F_ind)

    print '\nF_cond:{0} P_cond:{1}'.format(F_cond,P_cond)
    print 'F_ind:{0} P_ind:{1}'.format(F_ind,P_ind)
    #print 'F_interaction:{0} P_interaction:{1}'.format(F_interaction,P_in

    print '\nCondition:\nF({0},{1})={2}, p={3}'.format(condition_dof,remai
    print '\nBetween individuals:\nF({0},{1})={2}, p={3}'.format(individua

    print '\n'
    print 'Source of var, Sumof Squares, df, Mean square , F ratio
    print '----------------------------------------------------------------
    print '{0}, {1}, {2}, {3}, {4}, {5}'.format('Individual   ',individual
    print '{0}, {1}, {2}, {3}, {4}, {5}'.format('Condition    ',condition_
    print '{0}, {1}, {2}, {3}, {4}, {5}'.format('Remainder    ',remainder_
    print '{0}, {1}, {2}, {3}, {4}, {5}'.format('Total        ',total_sos,

def normality_test(individuals):
    print '\nNormality of data:'

    nd_latency_means = []
    sd_latency_means = []
    ad_latency_means = []
    for i,ind in individuals.iteritems():
        nd_latency_means = np.append(nd_latency_means, ind.nodist_mean())
        sd_latency_means = np.append(sd_latency_means, ind.sync_mean())
        ad_latency_means = np.append(ad_latency_means, ind.async_mean())

    print ' Shapiro-Wilks:'
    W, p = stats.shapiro (subsample(nd_latency_means, 25))
    print ' No Distractor, 25 sub-samples: W',W,'p-value',p,'(Reject Norma
    W, p = stats.shapiro (subsample(sd_latency_means, 25))
    print ' Sync Distractor, 25 sub-samples: W',W,'p-value',p
    W, p = stats.shapiro (subsample(ad_latency_means, 25))
    print ' Async Distractor, 25 sub-samples: W',W,'p-value',p

    print '\nSee also QQ Plots (set show_graph to 1)'
    # Quantile-Quantile Plot to show normality
    show_graph = 1
    if show_graph==1:
```

```
    %matplotlib inline
    f, (ax1, ax2, ax3) = plt.subplots(3)
    fig1 = sm.qqplot(nd_latency_means, fit=True, line='45',ax=ax1)
    ax1.set_title('ND')
    fig2 = sm.qqplot(sd_latency_means, fit=True, line='45',ax=ax2)
    ax2.set_title('SD')
    fig3 = sm.qqplot(ad_latency_means, fit=True, line='45', ax=ax3)
    ax3.set_title('AD')
    savename = 'images/latency_means_normplot.png'
    plt.savefig(savename)
    plt.show()

# Call the repeated measures ANOVA
individuals = readIndividuals()

# Could equalise replicants, but this would be entirely optional for repea
#individuals = equaliseReplicates(individuals)
# If we don't call equalise, then must exclude outliers anyway:
for i,ind in individuals.iteritems():
    ind.excludeOutliers()

normality_test(individuals)

repmeasures_anova(individuals)
```

```
/usr/local/lib/python2.7/dist-packages/numpy/core/_methods.py:59: RuntimeWarning: M
  warnings.warn("Mean of empty slice.", RuntimeWarning)



Normality of data:
 Shapiro-Wilks:
 No Distractor, 25 sub-samples: W 0.914870500565 p-value 0.0391994863749 (Reject No
 Sync Distractor, 25 sub-samples: W 0.845669388771 p-value 0.00145912088919
 Async Distractor, 25 sub-samples: W 0.962160229683 p-value 0.459158211946

See also QQ Plots (set show_graph to 1)
```

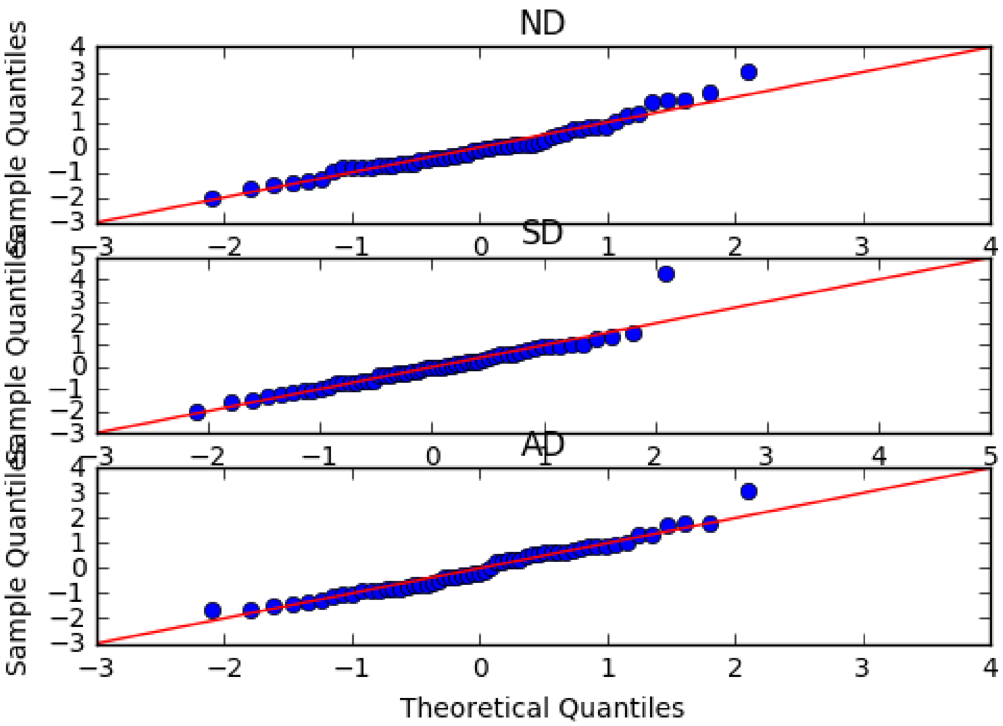


```
Grand mean: 327.018604194
total sos: 215033.599942 total dof: 164 total variance: 1311.18048745
total_sos: 215033.599942
condition_sos: 80551.4780108
individual_sos: 99090.3959277
remainder_sos: 35391.726004
remainder_variance: 327.701166704 remainder_sos: 35391.726004
total_sos: 215033.599942 condition_sos: 80551.4780108 indiv_sos: 99090.3959277 erro

F_cond:122.903862109 P_cond:1.11022302463e-16
F_ind:5.59963624925 P_ind:1.62092561595e-14

Condition:
F(2,108)=122.903862109, p=1.11022302463e-16

Between individuals:
F(54,108)=5.59963624925, p=1.62092561595e-14


Source of var, Sumof Squares, df, Mean square , F ratio         P
-------------------------------------------------------------------
Individual   , 99090.3959277, 54, 1835.00733199, 5.59963624925, 1.62092561595e-14
Condition    , 80551.4780108, 2, 40275.7390054, 122.903862109, 1.11022302463e-16
```

```
Remainder    , 35391.726004, 108, 327.701166704, -, -
Total        , 215033.599942, 164, -, -, -
```

1.8.1 Repeated measures ANOVA summary

The null hypotheses are that "condition does not change latency" and "individual does not affect latency".

A repeated measures anova, where I use the mean latency for each condition as a single data point for each individual shows a significant effect both for condition and for individual - the null hypotheses would be rejected in each case if the data fulfilled normality.

1.9 Output data for analysis in R

How many different stats systems can we use for one piece of data analysis? See various .r scripts which carry out much of the analysis reported in the paper.

This code block generates AnovaR.csv which contains the latencies and error_rates.csv which contains the error rates.

```
In [16]: # Output data in format suitable for R
         individuals = readIndividuals()

         # Optional:
         #individuals = equaliseReplicates(individuals)
         # or
         # If we don't call equalise, exclude outliers anyway:
         for i,ind in individuals.iteritems():
             ind.excludeOutliers()

         async_lines = 'condition_str,subj_id,num,type,error,correctmove,latency,om
         sync_lines = 'condition_str,subj_id,num,type,error,correctmove,latency,omi
         nodist_lines = 'condition_str,subj_id,num,type,error,correctmove,latency,o
         csvdata = 'idnum,latency,condition,condition_str,subj_id\n'
         csvtrialdata = 'subj_id,latency,condition_str\n'
         for i,ind in individuals.iteritems():
             csvdata += ind.outputDataForR()
             # Save this particular individuals latency values
             csvtrialdata += ind.writeDataForR()
             async_lines += ind.writeAsyncDataForR()
             sync_lines += ind.writeSyncDataForR()
             nodist_lines += ind.writeNoDistDataForR()

         print 'Writing AnovaR.csv...'
         f = open('AnovaR.csv', 'w')
         f.write(csvdata)
         f.close()

         print 'Writing AllTrials.csv...'
```

```
    f = open('AllTrials.csv', 'w')
    f.write(csvtrialdata)
    f.close()

    print 'Writing AsyncTrials.csv...'
    f = open('AsyncTrials.csv', 'w')
    f.write(async_lines)
    f.close()

    print 'Writing SyncTrials.csv...'
    f = open('SyncTrials.csv', 'w')
    f.write(sync_lines)
    f.close()

    print 'Writing NoDistTrials.csv...'
    f = open('NoDistTrials.csv', 'w')
    f.write(nodist_lines)
    f.close()

    print 'Writing error_rates.csv...'
    f = open('error_rates.csv', 'w')
    f.write ('idnum,error_rate,condition,condition_str,subj_id\n')
    for i,ind in readIndividuals().iteritems():
        subj_id = ind.subj_id
        if subj_id == 'NA':
            subj_id = 'NA_'
        f.write ('{0},{1},1,ND,{2}\n'.format(ind.idnum,ind.num_moveerrors_per_
        f.write ('{0},{1},2,SD,{2}\n'.format(ind.idnum,ind.num_moveerrors_per_
        f.write ('{0},{1},3,AD,{2}\n'.format(ind.idnum,ind.num_moveerrors_per_
    f.close()
```

```
Writing AnovaR.csv...
Writing AllTrials.csv...
Writing AsyncTrials.csv...
Writing SyncTrials.csv...
Writing NoDistTrials.csv...
Writing error_rates.csv...
```

1.10 ANOVA on latency ranks

This code block calls the R script **RankedAnova.r** and outputs the result. It means you have to have R installed (version 3.0.2 will match my system) with the packages nlme and effsize.

```
In [17]: from subprocess import CalledProcessError, check_output
         try:
             out = check_output(["R", "-q --file=RankedAnova.r"])
         except CalledProcessError as err:
```

```
                print 'Error for:',err.cmd,'with error code:',err.returncode
                print "\nOutput:\n\n",err.output
            else:
                for line in out.splitlines():
                    print line
```

```
> # I found guidance on Anovas in R here:
> # https://gribblelab.wordpress.com/2009/03/09/repeated-measures-anova-using-r/
> # and here:
> # https://seriousstats.wordpress.com/tag/rank-transformation/
>
> # Set factor contrasts option, important for aov() function.
> options(contrasts=c("contr.treatment","contr.treatment"))
>
> latdat <- read.csv('AnovaR.csv')
>
> # Using Linear Mixed Effects models
> require(nlme)
>
> # Compute ranks to carry out ANOVA on the ranks
> rlatency <- rank(latdat$latency)
> #
> # The formulae here are:
> # fixed: rlatency "is predicted by" condition_str
> # random: "is predicted by" "the mean" "given" "condition_str nested within subj_
> anova_ranked_cond <- lme(fixed = rlatency ~ condition_str, random = ~1|subj_id/co
> # ---------------------------------------------------------------------------
> print (summary(anova_ranked_cond))
Linear mixed-effects model fit by REML
 Data: latdat
       AIC      BIC    logLik
  1598.539 1617.065 -793.2697

Random effects:
 Formula: ~1 | subj_id
        (Intercept)
StdDev:    28.38689

 Formula: ~1 | condition_str %in% subj_id
        (Intercept) Residual
StdDev:    21.43293 9.895613

Fixed effects: rlatency ~ condition_str
                    Value Std.Error  DF    t-value p-value
(Intercept)     109.89091  4.978334 108  22.073834   0.000
condition_strND -69.54545  4.501691 108 -15.448740   0.000
condition_strSD -11.12727  4.501691 108  -2.471798   0.015
 Correlation:
```

```
                (Intr) cnd_ND
condition_strND -0.452
condition_strSD -0.452  0.500

Standardized Within-Group Residuals:
        Min          Q1         Med          Q3         Max
-0.86143597 -0.25401473  0.02105463  0.24965003  0.90271124

Number of Observations: 165
Number of Groups:
                   subj_id condition_str %in% subj_id
                        55                        165
> # -----------------------------------------------------------------------------
> print(anova(anova_ranked_cond))
              numDF denDF  F-value p-value
(Intercept)       1   108 382.1123  <.0001
condition_str     2   108 137.7248  <.0001
> # -----------------------------------------------------------------------------
> #
> # Showing residuals of ANOVA on ranks:
>
> setEPS()
> postscript(file='../paper/figures/anova_ranked_cond_resid.eps')
> plot(fitted(anova_ranked_cond), residuals(anova_ranked_cond))
> dev.off()
null device
          1
>
> png(filename='r_images/anova_ranked_cond_resid.png')
> plot(fitted(anova_ranked_cond), residuals(anova_ranked_cond))
> dev.off()
null device
          1
>
> # Carry out the LME on subj_id - the inverse of the above.
> anova_ranked_indiv <- lme(fixed = rlatency ~ subj_id, random = ~1|condition_str/s
> # -----------------------------------------------------------------------------
> print(anova(anova_ranked_indiv))
            numDF denDF   F-value p-value
(Intercept)     1   108 14.809621   2e-04
subj_id        54   108  5.337834  <.0001
> # -----------------------------------------------------------------------------
> setEPS()
> postscript(file='../paper/figures/anova_ranked_indiv_resid.eps')
> plot (fitted(anova_ranked_indiv), residuals(anova_ranked_indiv))
> dev.off()
null device
          1
```

```
>
> png (filename='r_images/anova_ranked_indiv_resid.png')
> plot (fitted(anova_ranked_indiv), residuals(anova_ranked_indiv))
> dev.off()
null device
          1
>
> #
> # Apply Wilcoxon Signed-Rank test for pairwise comparisons
> #
> # individual latencies
> nd <- latdat[latdat$condition_str == "ND",]$latency
> sd <- latdat[latdat$condition_str == "SD",]$latency
> ad <- latdat[latdat$condition_str == "AD",]$latency
> #
> # paired=TRUE indicates signed rank test
> # Note in wilcox.test, V is what R calls W.
> print(wilcox.test(nd, sd, paired=TRUE))

        Wilcoxon signed rank test with continuity correction

data:  nd and sd
V = 3, p-value = 1.344e-10
alternative hypothesis: true location shift is not equal to 0

> print(wilcox.test(nd, ad, paired=TRUE))

        Wilcoxon signed rank test with continuity correction

data:  nd and ad
V = 1, p-value = 1.203e-10
alternative hypothesis: true location shift is not equal to 0

> print(wilcox.test(ad, sd, paired=TRUE, exact=T))

        Wilcoxon signed rank test

data:  ad and sd
V = 1042, p-value = 0.02208
alternative hypothesis: true location shift is not equal to 0

>
> #
> # Cliff's delta
> require(effsize)
> #
> # Between No Distractor and Synchronous Distractor:
> cliff.delta(nd,sd)
```

```
Cliff's Delta

delta estimate: -0.7500826 (large)
95 percent confidence interval:
       inf        sup
-0.8601172 -0.5733459
> #
> # Between No Distractor and Asynchronous Distractor:
> cliff.delta(nd,ad)

Cliff's Delta

delta estimate: -0.8009917 (large)
95 percent confidence interval:
       inf        sup
-0.9070022 -0.5995156
> #
> # Between Asynchronous Distractor and Synchronous Distractor:
> cliff.delta(ad,sd)

Cliff's Delta

delta estimate: 0.1768595 (small)
95 percent confidence interval:
        inf         sup
-0.03903052  0.37697444
>
```

```
In [18]: print 'Residuals (Condition):'
         Image(filename='r_images/anova_ranked_cond_resid.png')
```

```
Residuals (Condition):
```

```
Out[18]:
```

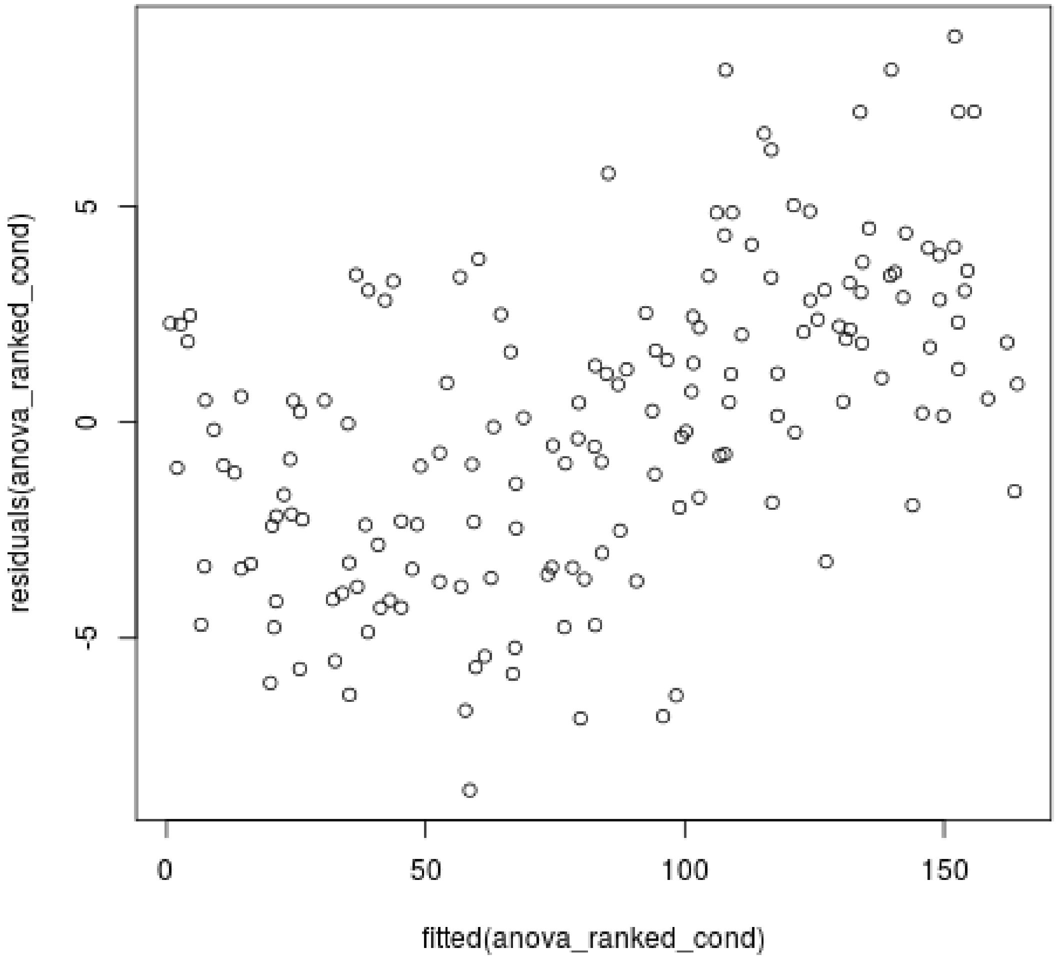


```
In [19]: print 'Residuals (Individual)'
         Image(filename='r_images/anova_ranked_indiv_resid.png')

Residuals (Individual)


Out[19]:
```

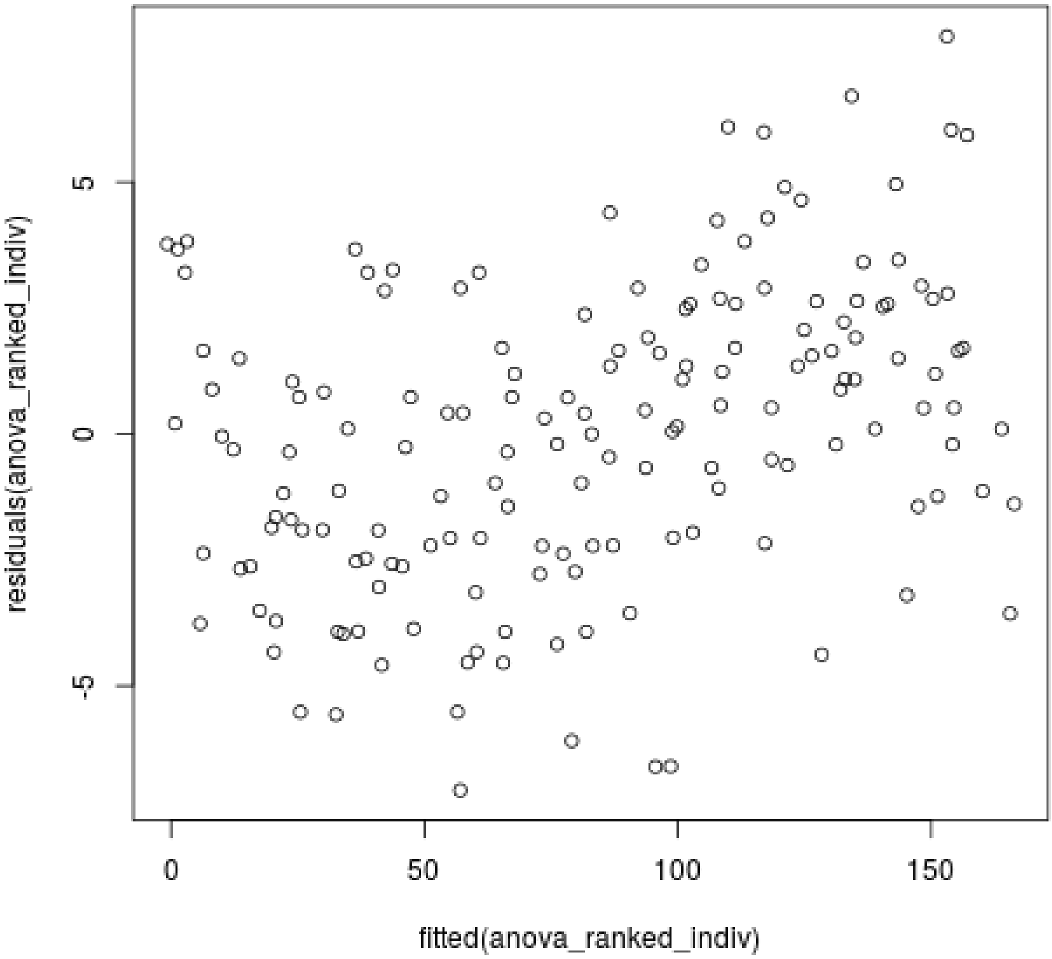

1.11 Linear Mixed Effects model

This analysis is used in the paper.

```
In [20]: from subprocess import CalledProcessError, check_output
         try:
             out = check_output(["R", "-q --file=LinearMixedEffects.r"])
         except CalledProcessError as err:
             print 'Error for:',err.cmd,'with error code:',err.returncode
             print "\nOutput:\n\n",err.output
         else:
             for line in out.splitlines():
                 print line
```

```
> ## This script computes linear mixed effects model for the latency data.
>
> ## I found guidance on Anovas/Linear models in R here:
> ## https://gribblelab.wordpress.com/2009/03/09/repeated-measures-anova-using-r/
> ## and here:
> ## https://seriousstats.wordpress.com/tag/rank-transformation/
>
> ##NB: source like this: source('Nonranked.r', print.eval=TRUE)
>
> ## Set factor contrasts option, important for aov() function.
> options(contrasts=c("contr.treatment","contr.treatment"))
>
> latdat <- read.csv('AnovaR.csv')
>
> ## Using Linear Mixed Effects models
> require(nlme)
>
> ## Compute ranks to carry out ANOVA on the ranks
> ##rlatency <- rank(latdat$latency)
>
> ## The formulae here are:
> ## fixed effects: latency "is predicted by" condition_str as a factor.
> ## random effects: "is predicted by" "subj_id"
> nonranked <- lme(fixed = latency ~ condition_str, random = ~1|subj_id, data=latda
> ## ---------------------------------------------------------------------------------
> summary(nonranked)
Linear mixed-effects model fit by REML
 Data: latdat
       AIC      BIC    logLik
  1513.105 1528.542 -751.5523

Random effects:
 Formula: ~1 | subj_id
        (Intercept) Residual
StdDev:    22.41507 18.10252

Fixed effects: latency ~ condition_str
                   Value Std.Error  DF   t-value p-value
(Intercept)     347.1905  3.885022 108  89.36643  0.0000
condition_strND -50.9244  3.452015 108 -14.75209  0.0000
condition_strSD  -9.5913  3.452015 108  -2.77847  0.0064
 Correlation:
                (Intr) cnd_ND
condition_strND -0.444
condition_strSD -0.444  0.500

Standardized Within-Group Residuals:
        Min           Q1          Med           Q3          Max
```

```
-1.84077745 -0.53695422 -0.01665622  0.51861454  3.00485476

Number of Observations: 165
Number of Groups: 55
> ## ---------------------------------------------------------------------------
> anova(nonranked)
              numDF denDF  F-value p-value
(Intercept)       1   108 9615.924  <.0001
condition_str     2   108  122.904  <.0001
>
> ## Method="ML" is equivalent in the lme() function to REML=FALSE for the lmer fur
> nonranked.null <- lme(latency ~ 1, random= ~1|subj_id, data=latdat, method="ML")
> nonranked.mdl <- lme(latency ~ condition_str, random= ~1|subj_id, data=latdat, me
> anova(nonranked.null, nonranked.mdl)
               Model df      AIC      BIC    logLik   Test  L.Ratio p-value
nonranked.null     1  3 1652.196 1661.514 -823.0980
nonranked.mdl      2  5 1525.668 1541.197 -757.8338 1 vs 2 130.5285  <.0001
>
> setEPS()
> postscript(file='../paper/figures/lme_latency_resid1.eps')
> plot(nonranked, resid(., type = "p") ~ fitted(.) | condition_str, abline = 0, xla
> dev.off()
null device
          1
>
> setEPS()
> postscript(file='../paper/figures/lme_latency_resid2.eps')
> plot(nonranked, subj_id ~ resid(.))
> dev.off()
null device
          1
>
> setEPS()
> postscript(file='../paper/figures/lme_latency_fits.eps')
> plot(nonranked, latency ~ fitted(.) | subj_id, abline = c(0,1))
> dev.off()
null device
          1
>
```

1.12 Individual analysis of latencies

Now run **Bootstrap_indiv.r** to compute per-individual results.

```
In [21]: from subprocess import CalledProcessError, check_output
         try:
             out = check_output(["R", "-q --file=Bootstrap_indiv.r"])
```

```
        except CalledProcessError as err:
            print 'Error for:',err.cmd,'with error code:',err.returncode
            print "\nOutput:\n\n",err.output
        else:
            for line in out.splitlines():
                #if line[0] != ">" and line[0] != "+":
                    print line
```

```
> # Here, I'm going to bootstrap the latencies of each individual
>
> set.seed(19742016)
>
> # This loads individual latencies. each file contains two columns,
> # latency and condition_str. The latencies are all from non-error
> # events.
> fns <- list.files(pattern="IndDat*")
>
> fasterSlower <- c()
>
> sdadNoDiff<-0
> adFaster<-1
> sdFaster<-2
>
> ndall <- c()
> sdall <- c()
> adall <- c()
> for (indiv in fns) {
+
+     latdat <- read.csv(indiv)
+
+     # Extract latencies for the three conditions
+     nd <- latdat[latdat$condition_str == "ND",]$latency
+     sd <- latdat[latdat$condition_str == "SD",]$latency
+     ad <- latdat[latdat$condition_str == "AD",]$latency
+
+     # Make vectors of all the latencies together, for all individuals
+     ndall <-c(ndall, nd)
+     sdall <-c(sdall, sd)
+     adall <-c(adall, ad)
+
+     lat.n <- 1000
+
+     # Plot will show that 87% of the possible means that I could have
+     # measured for SD and AD were different with
+     lat.highconf <- 0.9
+     lat.lowconf <- 1-lat.highconf
+     lat.confint <- 1 - 2*lat.lowconf
+
```

```
+      # Colour scheme
+      lat.ndcol <- "black"
+      lat.sdcol <- "steelblue"
+      lat.adcol <- "red"
+
+      lat.minx <- min(c(nd,sd,ad))
+      lat.maxx <- max(c(nd,sd,ad))
+      lat.miny <- 0
+      lat.maxy <- 0.15
+
+      # ND is blue
+      lat.nd.mean <- numeric(lat.n) # numeric vector lat.n long
+      # This is a bootstrap loop:
+      for(i in 1:lat.n) {
+         this.samp <- nd[ sample(length(nd), length(nd), replace=TRUE) ]
+         lat.nd.mean[i] <- mean(this.samp)
+      }
+      df <- density(lat.nd.mean, n=1024)
+
+      setEPS()
+      # This fails to make the eps files (dev.copy fails). However, we don't really
+      postscript(file=sprintf('../paper/figures/bootstrap_indiv_%s.eps', indiv))
+      dev.copy (png, filename=sprintf('r_images/bootstrap_indiv_%s.png', indiv))
+      plot(df, lwd=3, col=lat.ndcol, ylim=range(lat.miny,lat.maxy), xlim=range(lat.
+      abline(v=mean(nd), lty=2, lwd=2, col=lat.ndcol)
+      q5nd <- quantile(lat.nd.mean,lat.lowconf)
+      abline(v=q5nd[1], lty=6, lwd=2, col=lat.ndcol)
+      q95nd <- quantile(lat.nd.mean,lat.highconf)
+      abline(v=q95nd[1], lty=6, lwd=2, col=lat.ndcol)
+
+
+      # SD is dark red
+      lat.sd.mean <- numeric(lat.n) # numeric vector lat.n long
+      for(i in 1:lat.n) {
+         this.samp <- sd[ sample(length(sd), length(sd), replace=TRUE) ]
+         lat.sd.mean[i] <- mean(this.samp)
+      }
+      df <- density(lat.sd.mean, n=1024)
+      lines(df, lwd=3, col=lat.sdcol, xlim=range(280:365))
+      abline(v=mean(sd), lty=2, lwd=2, col=lat.sdcol)
+      q5sd <- quantile(lat.sd.mean,lat.lowconf)
+      abline(v=q5sd[1], lty=6, lwd=2, col=lat.sdcol)
+      q95sd <- quantile(lat.sd.mean,lat.highconf)
+      abline(v=q95sd[1], lty=6, lwd=2, col=lat.sdcol)
+
+
+      # AD is black
+      lat.ad.mean <- numeric(lat.n) # numeric vector lat.n long
```

```
+     for(i in 1:lat.n) {
+        this.samp <- ad[ sample(length(ad), length(ad), replace=TRUE) ]
+        lat.ad.mean[i] <- mean(this.samp)
+     }
+     df <- density(lat.ad.mean, n=1024)
+     lines(df, lwd=3, col=lat.adcol, xlim=range(280:365))
+     abline(v=mean(ad), lty=2, lwd=2, col=lat.adcol)
+     q5ad <- quantile(lat.ad.mean,lat.lowconf)
+     abline(v=q5ad[1], lty=6, lwd=2, col=lat.adcol)
+     q95ad <- quantile(lat.ad.mean,lat.highconf)
+     abline(v=q95ad[1], lty=6, lwd=2, col=lat.adcol)
+
+
+     # Stick in a legend
+     legend (320,0.15, bg="white",
+             c("PDF, ND resampled latencies","ND mean latency",
+               paste("ND latency conf. interval",lat.confint),"ibid, SD","ibid, AD
+             lty=c(1,2,6,1,1),
+             lwd=c(3,2,2,3,3),
+             col=c(lat.ndcol,lat.ndcol,lat.ndcol,lat.sdcol,lat.adcol))
+
+     # Close plotting devices
+     dev.off(dev.prev())
+     dev.off()
+
+     if (lat.sd.mean < lat.ad.mean) {
+         if (q5ad < q95sd) {
+             # overlap SD==AD
+             fasterSlower[indiv] <- sdadNoDiff
+         } else {
+             # no overlap, SD faster
+             fasterSlower[indiv] <- sdFaster
+         }
+     } else {
+         if (q5sd < q95ad) {
+             # overlap SD==AD
+             fasterSlower[indiv] <- sdadNoDiff
+         } else {
+             # no overlap AD faster
+             fasterSlower[indiv] <- adFaster
+         }
+     }
+ }
>
>
> print (sprintf ("%d individuals were faster in the AD condition", length(fasterSl
[1] "6 individuals were faster in the AD condition"
> print (sprintf ("%d individuals were faster in the SD condition", length(fasterSl
```

```
[1] "13 individuals were faster in the SD condition"
> print (sprintf ("%d individuals were no faster in either AD or SD", length(faster
[1] "36 individuals were no faster in either AD or SD"
>
> # Output the individuals from fasterSlower in a form suitable for copy
> # & paste into python, which is formatted like this:
> #
> # fasterSyncSubjects = ['CD1','CD2','CP','EC1','EC2','EF','IR','RF','RQ','SB2']
> fssNames <- c()
> fss <- "fasterSyncSubjects = ["
> for (s in names(fasterSlower[fasterSlower==sdFaster])) {
+     # Process name, remove "IndDat" and ".csv":
+     s <- sub ("IndDat", "", s)
+     s <- sub (".csv", "", s)
+     fssNames <- c(fssNames, s)
+     # Switch the R-form of "NA" back to the original as used in python:
+     s <- sub ("NA_", "NA", s)
+     fss <- sprintf ("%s '%s',", fss, s)
+ }
> fss <- sprintf ("%s]",fss)
> # Get rid of last ','
> fss <- sub (",]", " ]", fss)
> print (sprintf("fasterSyncSubjects (N=%d) python list code:", length(fssNames)))
[1] "fasterSyncSubjects (N=13) python list code:"
> print (fss)
[1] "fasterSyncSubjects = [ 'AB1_', 'CD1', 'CD2', 'CH', 'CP', 'EC1', 'EC2', 'EF', '
>
>
> fasNames <- c()
> fas <- "fasterAsyncSubjects = ["
> for (s in names(fasterSlower[fasterSlower==adFaster])) {
+     s <- sub ("IndDat", "", s)
+     s <- sub (".csv", "", s)
+     fasNames <- c(fasNames, s)
+     s <- sub ("NA_", "NA", s)
+     fas <- sprintf ("%s '%s',", fas, s)
+ }
> fas <- sprintf ("%s]",fas)
> fas <- sub (",]", " ]", fas)
> print (sprintf("fasterAsyncSubjects (N=%d) python list code:", length(fasNames)))
[1] "fasterAsyncSubjects (N=6) python list code:"
> print (fas)
[1] "fasterAsyncSubjects = [ 'AW3_', 'EJ', 'LH1', 'NA', 'PO', 'SF' ]"
>
>
> ndNames <- c()
> nds <- "noDiffSubjects = ["
> for (s in names(fasterSlower[fasterSlower==sdadNoDiff])) {
```

```
+     s <- sub ("IndDat", "", s)
+     s <- sub (".csv", "", s)
+     ndNames <- c(ndNames, s)
+     s <- sub ("NA_", "NA", s)
+     nds <- sprintf ("%s '%s',", nds, s)
+ }
> nds <- sprintf ("%s]",nds)
> nds <- sub (",]", " ]", nds)
> print (sprintf("noDiffSubjects (N=%d) python list code:", length(ndNames)))
[1] "noDiffSubjects (N=36) python list code:"
> print (nds)
[1] "noDiffSubjects = [ 'AL_', 'AM', 'AS', 'AW1_', 'AW2_', 'BG', 'CM', 'EB1', 'EB2'
>
>
> #
> # Now do analysis on ndall etc.
> #
>
> # Save some variables that are used in Bootstrap_all and Err.r
> save (ndall, sdall, adall, fssNames, fasNames, ndNames, file="all_latencies.rdat'
>
> print ('You can now call Bootstrap_all.r for "the groupstrap"')
[1] "You can now call Bootstrap_all.r for \"the groupstrap\""
> print ('You can also call Err.r for the per-group error analysis')
[1] "You can also call Err.r for the per-group error analysis"
>
```

```
In [22]: print 'An example individual set of latency bootstrap means'
         Image(filename='r_images/bootstrap_indiv_IndDatSF.csv.png')
```

```
An example individual set of latency bootstrap means
```

Out[22]:

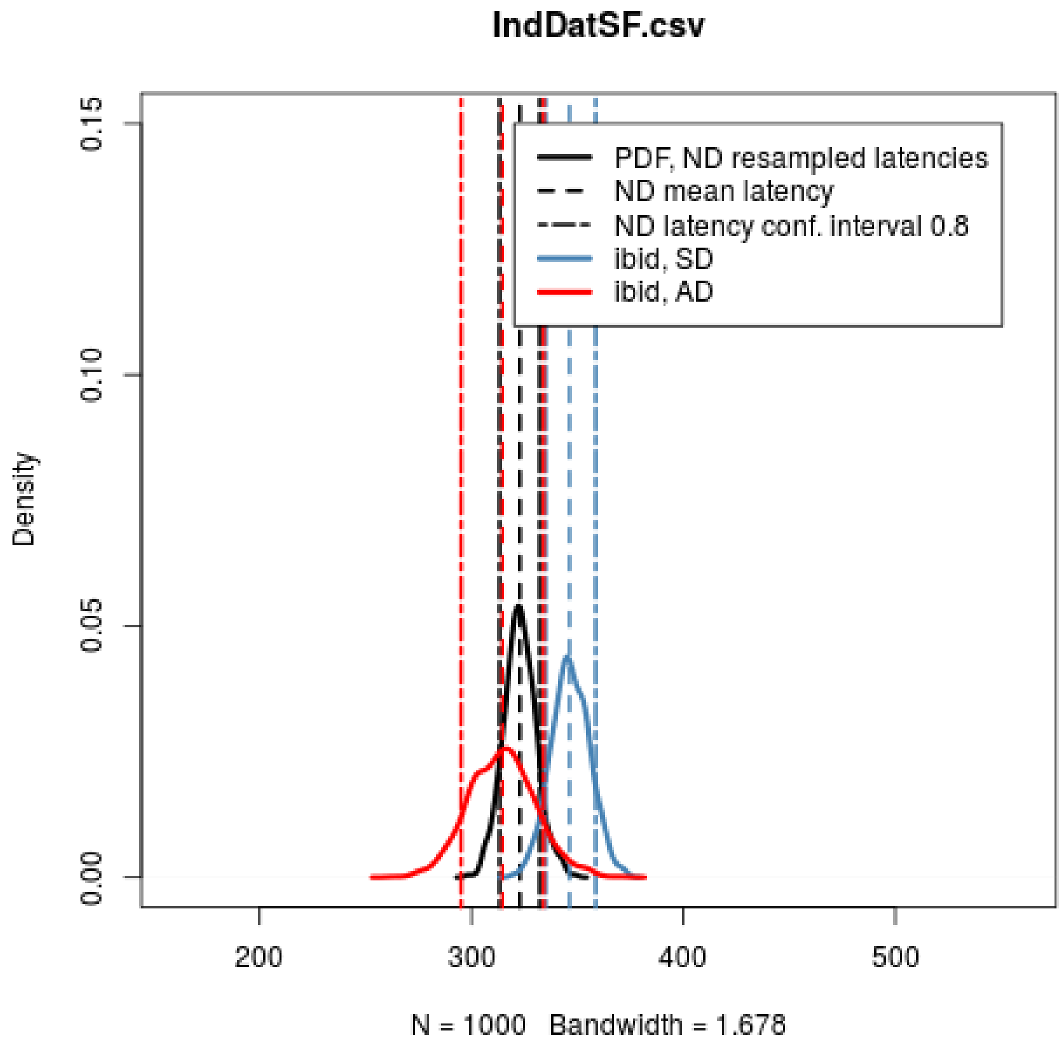

1.13 Bootstrap analysis of latencies

After calling **Bootstrap_indiv.r**, **Bootstrap_all.r** can be called.

```
In [23]: from subprocess import CalledProcessError, check_output
         try:
             out = check_output(["R", "-q --file=Bootstrap_all.r"])
         except CalledProcessError as err:
             print 'Error for:',err.cmd,'with error code:',err.returncode
             print "\nOutput:\n\n",err.output
         else:
             print 'Success'
             for line in out.splitlines():
                 print line
```

```
Success
> #
> # Now do analysis on ndall etc.
> #
> # This assumes ndall, sdall and adall have been generated from Bootstrap_indiv.r
> #
> # This is the bootstrapping version of the group analysis.
> #
>
> load(file="all_latencies.rdat")
>
> filesuffix <- "all"
> # To call this, you need to have ndall, sdall and adall in the workspace:
> source('Bootstrap_all_main.r')
[1] "number of bootstrap resamples: 2013"
[1] "number of ND samples: 2013"
[1] "number of SD samples: 2013"
[1] "number of AD samples: 2013"
[1] "ND std err estimate: 1.13558190938641"
[1] "SD std err estimate: 1.25663521373969"
[1] "AD std err estimate: 1.78537498107593"
[1] "Std. error of the median estimate for ND: 1.18152362654073"
[1] "Std. error of the mean estimate for SD: 1.24741509357161"
[1] "Std. error of the mean estimate for AD: 1.72853506122582"
[1] "latency means, ND, SD, AD"
[1] 295.1411 336.0313 344.9548
[1] "MADs of latencies"
[1] 44.4780 50.4084 65.2344
[1] "SDs of latencies"
[1] 51.06440 56.52306 78.95953
[1] "SE of mean"
[1] 1.135582 1.256635 1.785375
[1] "95% confidence"
     95%      95%      95%
1.860606 2.094585 2.915549
[1] "raw distributions"
>
```

```
In [24]: print 'Latency measurement distributions, all subjects'
         Image(filename='r_images/data_density_all.png')
```

```
Latency measurement distributions, all subjects
```

Out[24]:

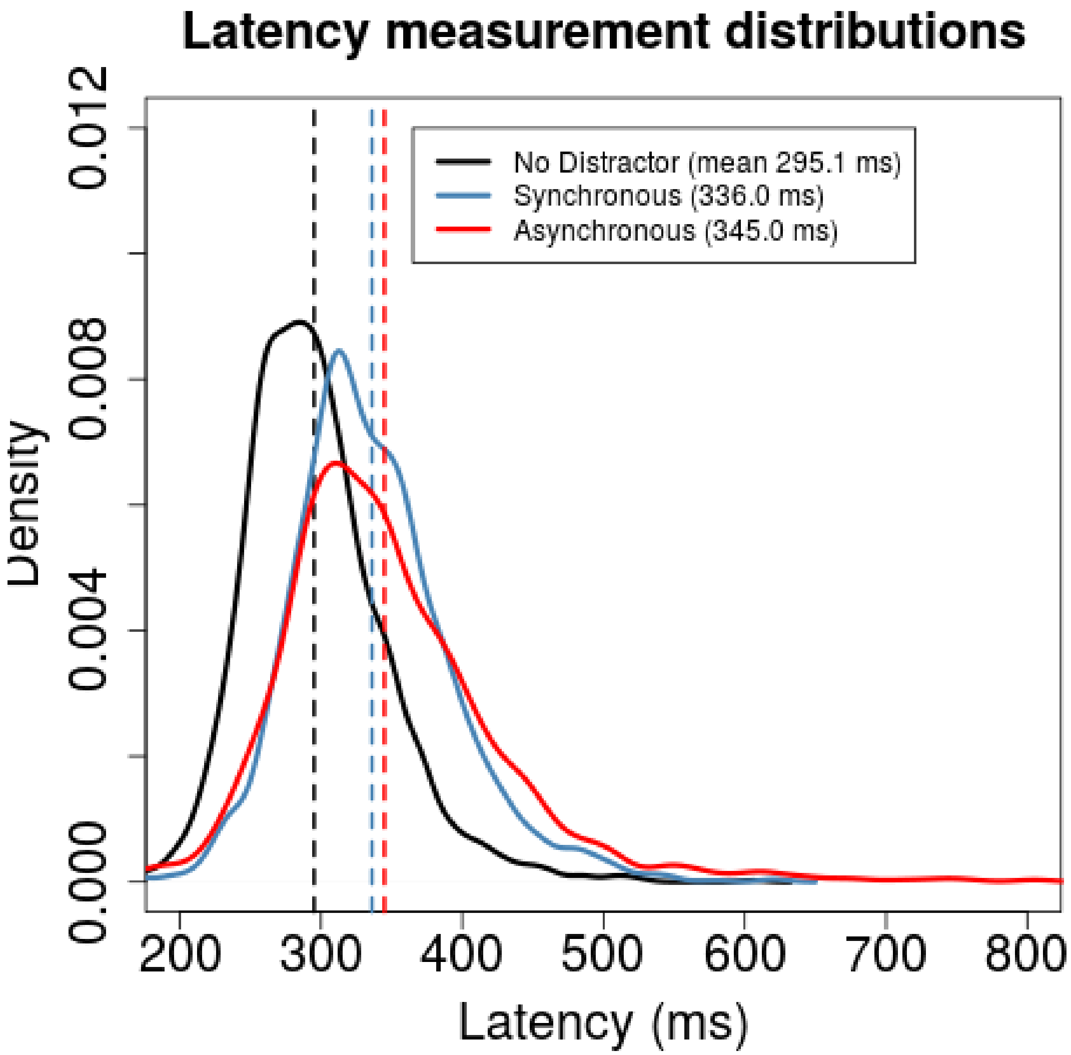


```
In [25]: print 'Bootstrapped latency means, all subjects'
         Image(filename='r_images/bootstrapped_means_all.png')
```

Bootstrapped latency means, all subjects

Out[25]:

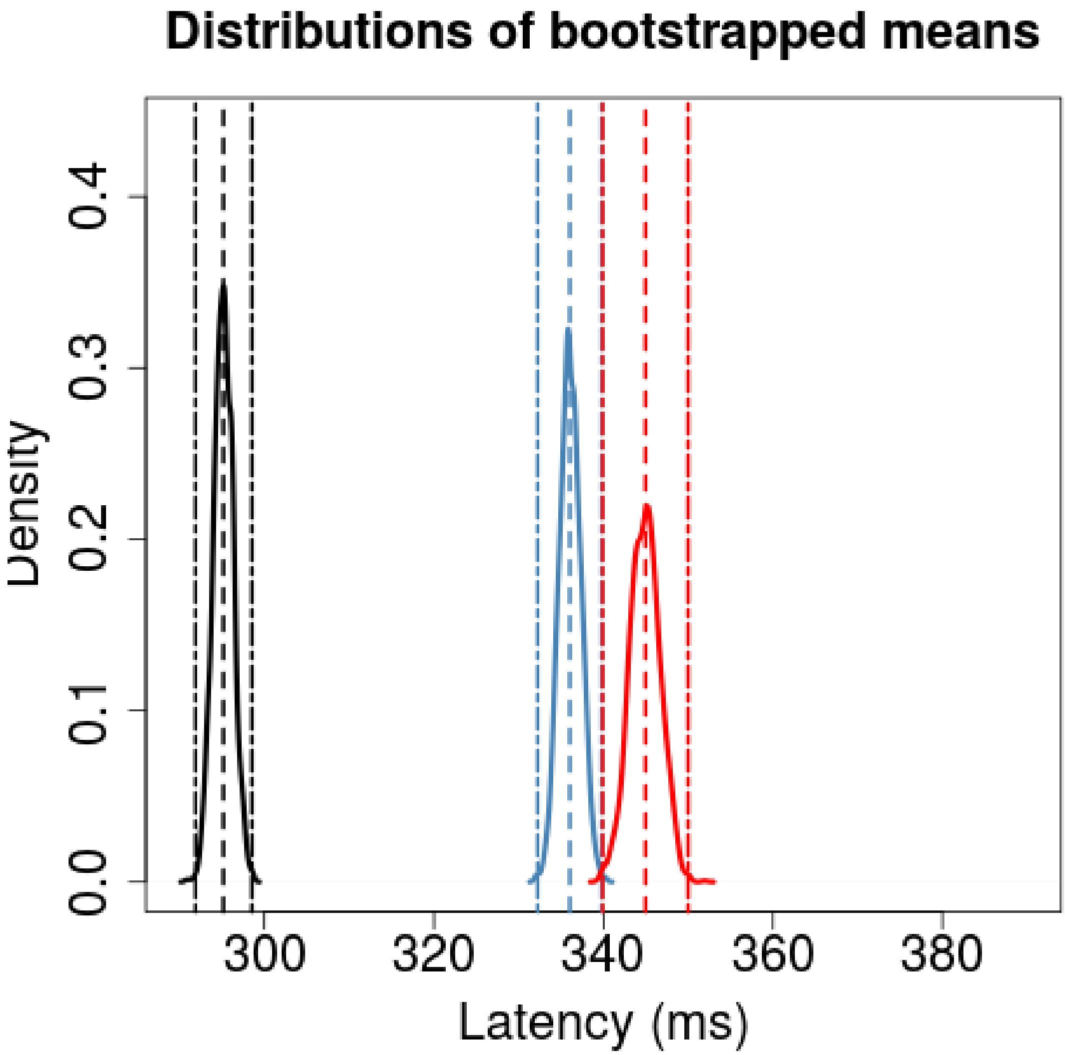


```
In [26]: print 'Means again, this time with 95% confidence intervals as error bars'
         Image(filename='r_images/meanmad_all.png')
```

```
Means again, this time with 95% confidence intervals as error bars
```

Out[26]:

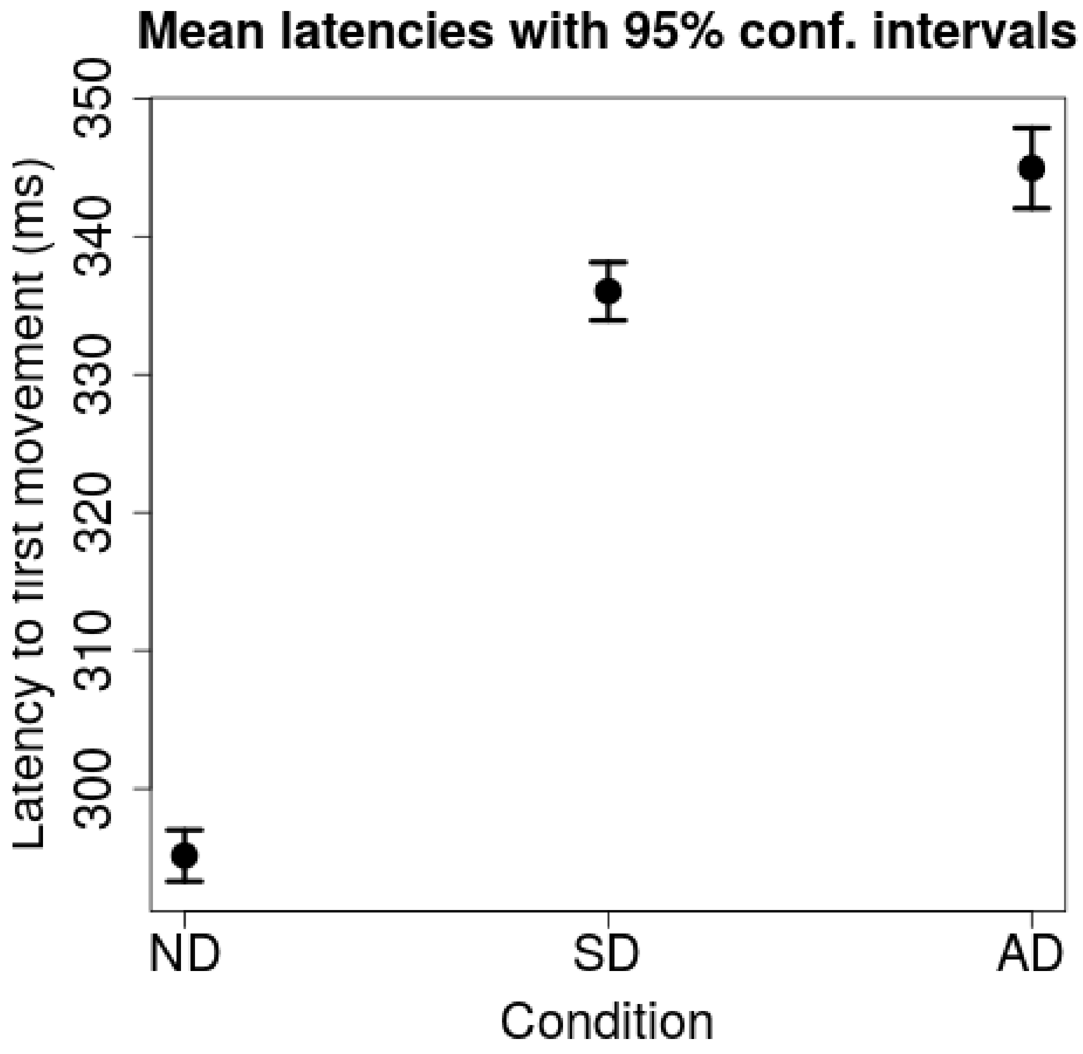

1.13.1 Two-sample bootstrap analysis

The following analysis shows difference between means of pairs of conditions and also applies an hypothesis test to estimate the probability of the data having been drawn from the same population.

Note that the hypothesis testing code requires lots of RAM. To obtain the same results reported in the paper, source the file Bootstram_two_bigmem.r on a machine with 128 GB of RAM.

```
In [27]: from subprocess import CalledProcessError, check_output
         try:
             out = check_output(["R", "-q --file=Bootstrap_two.r"])
         except CalledProcessError as err:
             print 'Error for:',err.cmd,'with error code:',err.returncode
```

```
            print "\nOutput:\n\n",err.output
        else:
            print 'Success'
            for line in out.splitlines():
                print line
```

```
Success
> #
> # Now do analysis on ndall etc.
> #
> # This assumes ndall, sdall and adall have been generated from
> # Bootstrap_indiv.r
> #
> # This is an updated version of the bootstrapping group analysis,
> # following reviewer comments that the analysis was not correct.
> #
>
> set.seed(1974201701)
>
> load(file="all_latencies.rdat")
>
> filesuffix <- "twosamp"
> source('Bootstrap_all_twosamples.r')
>
> #
> # Bootstrap the difference between the means of pairs of distributions:
> #
> print("-----------------------------------------")
[1] "-----------------------------------------"
> print("Bootstrap analysis of difference of means")
[1] "Bootstrap analysis of difference of means"
> ndsd <- b.diffste(sdall, ndall, 1024)
> print (sprintf("SD ND difference is %f, standard error estimate: %f", ndsd$meandi
[1] "SD ND difference is 40.890214, standard error estimate: 1.669482"
> #ndsd$df = density(ndsd$diffs)
> #plot (ndsd$df)
>
> ndad <- b.diffste(adall, ndall, 1024)
> print (sprintf("AD ND difference is %f, standard error estimate: %f", ndad$meandi
[1] "AD ND difference is 49.813711, standard error estimate: 2.067093"
>
> sdad <- b.diffste(adall, sdall, 1024)
> print (sprintf("AD SD difference is %f, standard error estimate: %f", sdad$meandi
[1] "AD SD difference is 8.923497, standard error estimate: 2.238012"
> #sdad$df = density(sdad$diffs, n=1024)
> #plot (sdad$df)
>
>
```

```
> #
> # Apply a hypothesis test to obtain the probability that the pairs of
> # distributions are drawn from the same population.
> #
>
> #print ("Algo 16.1")
>
> # ASL < 1/4e6 (2.5e-7) with B=4e6
> #ndsd_t <- b.ttest(sdall, ndall, 1000)
> #b.showsiglev (ndsd_t, "SD vs ND")
>
> # ASL < 1/4e6 (2.5e-7)
> #ndad_t <- b.ttest(adall, ndall, 1000)
> #b.showsiglev (ndad_t, "AD vs ND")
>
> # With B=1000000, get asl=0.000016
> #sdad_t <- b.ttest(adall, sdall, 1000)
> #b.showsiglev (sdad_t, "AD vs SD")
>
>
> print("---------------------------------------")
[1] "---------------------------------------"
> print ("Studentized bootstrapped hypothesis test (Algo 16.2)")
[1] "Studentized bootstrapped hypothesis test (Algo 16.2)"
> # ndsd does better than 1e-6 (set B to 1000000)
> ndsd_tst <- b.studentized_ttest(sdall, ndall, 1000)
> b.showsiglev (ndsd_tst, "SD vs ND")
[1] "Achieved significance level for SD vs ND < 0.001000"
>
> # ndad does better than 3e-7 (set B to 3000000)
> ndad_tst <- b.studentized_ttest(adall, ndall, 1000)
> b.showsiglev (ndad_tst, "AD vs ND")
[1] "Achieved significance level for AD vs ND < 0.001000"
>
> # sdad produced asl of 0.000012 for B=1000000
> sdad_tst <- b.studentized_ttest(adall, sdall, 1000)
> b.showsiglev (sdad_tst, "AD vs SD")
[1] "Achieved significance level for AD vs SD < 0.001000"
>
> print("---------------------------------------")
[1] "---------------------------------------"
>
```

1.14 Bootstrap analysis on latencies split into error and no error groups

Here, we analyse whether those trials which are "movement error" trials have latencies which are different from those trials which are "correct movement" trials.

```
In [28]: from subprocess import CalledProcessError, check_output
         try:
             out = check_output(["R", "-q --file=Bootstrap_two_errornoerror.r"])
         except CalledProcessError as err:
             print 'Error for:',err.cmd,'with error code:',err.returncode
             print "\nOutput:\n\n",err.output
         else:
             print 'Success'
             for line in out.splitlines():
                 print line
```

```
Success
> ad <- read.csv('AsyncTrials.csv')
> sd <- read.csv('SyncTrials.csv')
> nd <- read.csv('NoDistTrials.csv')
>
> ## Create lists of errored and non-errored latencies. Note that we omit latencies
> tr.noerror.ad <- ad[which (ad$type == 1 & ad$latency > 0 & ad$latency < 1000 & ad
> tr.noerror.sd <- sd[which (sd$type == 1 & sd$latency > 0 & sd$latency < 1000 & sd
> tr.noerror.nd <- nd[which (nd$type == 1 & nd$latency > 0 & nd$latency < 1000 & nd
> tr.error.ad <- ad[which (ad$type == 1 & ad$latency > 0 & ad$latency < 1000 & ad$c
> tr.error.sd <- sd[which (sd$type == 1 & sd$latency > 0 & sd$latency < 1000 & sd$c
> tr.error.nd <- nd[which (nd$type == 1 & nd$latency > 0 & nd$latency < 1000 & nd$c
>
> set.seed(1974201702)
>
> print('Means:')
[1] "Means:"
> print (sprintf('AD, noerror: %f', mean(tr.noerror.ad$latency)))
[1] "AD, noerror: 344.943996"
> print (sprintf('AD, error: %f', mean(tr.error.ad$latency)))
[1] "AD, error: 229.032258"
> print (sprintf('SD, noerror: %f', mean(tr.noerror.sd$latency)))
[1] "SD, noerror: 335.923458"
> print (sprintf('SD, error: %f', mean(tr.error.sd$latency)))
[1] "SD, error: 274.435484"
> print (sprintf('ND, noerror: %f', mean(tr.noerror.nd$latency)))
[1] "ND, noerror: 294.902810"
> print (sprintf('ND, error: %f', mean(tr.error.nd$latency)))
[1] "ND, error: 218.868421"
> print("-----------------------------------------")
[1] "-----------------------------------------"
>
> # Load two sample bootstrapping functions:
> source('Bootstrap_all_twosamples.r')
>
> tr.error.ndsd <- b.diffste(tr.error.sd$latency, tr.error.nd$latency, 1024)
> print (sprintf("For error trials, SD ND difference is %f, standard error estimate
```

```
+                 tr.error.ndsd$meandiff, tr.error.ndsd$stderr))
[1] "For error trials, SD ND difference is 55.567063, standard error estimate: 21.4
>
> tr.error.ndad <- b.diffste(tr.error.ad$latency, tr.error.nd$latency, 1024)
> print (sprintf("For error trials, AD ND difference is %f, standard error estimate
+                 tr.error.ndad$meandiff, tr.error.ndad$stderr))
[1] "For error trials, AD ND difference is 10.163837, standard error estimate: 26.6
>
> tr.error.sdad <- b.diffste(tr.error.ad$latency, tr.error.sd$latency, 1024)
> print (sprintf("For error trials, AD SD difference is %f, standard error estimate
+                 tr.error.sdad$meandiff, tr.error.sdad$stderr))
[1] "For error trials, AD SD difference is -45.403226, standard error estimate: 19.
>
>
>
> tr.noerror.ndsd <- b.diffste(tr.noerror.sd$latency, tr.noerror.nd$latency, 1024)
> print (sprintf("For noerror trials, SD ND difference is %f, standard error estima
+                 tr.noerror.ndsd$meandiff, tr.noerror.ndsd$stderr))
[1] "For noerror trials, SD ND difference is 41.020648, standard error estimate: 1.
>
> tr.noerror.ndad <- b.diffste(tr.noerror.ad$latency, tr.noerror.nd$latency, 1024)
> print (sprintf("For noerror trials, AD ND difference is %f, standard error estima
+                 tr.noerror.ndad$meandiff, tr.noerror.ndad$stderr))
[1] "For noerror trials, AD ND difference is 50.041186, standard error estimate: 1.
>
> tr.noerror.sdad <- b.diffste(tr.noerror.ad$latency, tr.noerror.sd$latency, 1024)
> print (sprintf("For noerror trials, AD SD difference is %f, standard error estima
+                 tr.noerror.sdad$meandiff, tr.noerror.sdad$stderr))
[1] "For noerror trials, AD SD difference is 9.020539, standard error estimate: 2.1
>
>
> print("-----------------------------------------")
[1] "-----------------------------------------"
> print ("Studentized bootstrapped hypothesis test (Algo 16.2) for No Error data")
[1] "Studentized bootstrapped hypothesis test (Algo 16.2) for No Error data"
> ndsd_tst <- b.studentized_ttest(tr.noerror.sd$latency, tr.noerror.nd$latency, 100
> b.showsiglev (ndsd_tst, "SD vs ND")
[1] "Achieved significance level for SD vs ND < 0.000100"
>
> ndad_tst <- b.studentized_ttest(tr.noerror.ad$latency, tr.noerror.nd$latency, 100
> b.showsiglev (ndad_tst, "AD vs ND")
[1] "Achieved significance level for AD vs ND < 0.000100"
>
> sdad_tst <- b.studentized_ttest(tr.noerror.ad$latency, tr.noerror.sd$latency, 100
> b.showsiglev (sdad_tst, "AD vs SD")
[1] "Achieved significance level for AD vs SD < 0.000100"
>
> print("-----------------------------------------")
```

```
[1] "---------------------------------------"
> print ("Studentized bootstrapped hypothesis test (Algo 16.2) for Error data")
[1] "Studentized bootstrapped hypothesis test (Algo 16.2) for Error data"
> ndsd_tst <- b.studentized_ttest(tr.error.sd$latency, tr.error.nd$latency, 1000)
> b.showsiglev (ndsd_tst, "SD vs ND")
[1] "Achieved significance level for SD vs ND = 0.020000"
>
> ndad_tst <- b.studentized_ttest(tr.error.ad$latency, tr.error.nd$latency, 1000)
> b.showsiglev (ndad_tst, "AD vs ND")
[1] "Achieved significance level for AD vs ND = 0.335000"
>
> sdad_tst <- b.studentized_ttest(tr.error.ad$latency, tr.error.sd$latency, 1000)
> b.showsiglev (sdad_tst, "AD vs SD")
[1] "Achieved significance level for AD vs SD = 0.977000"
>
> print("---------------------------------------")
[1] "---------------------------------------"
>
> print("Does error/noerror achieve significant effect in each condition?")
[1] "Does error/noerror achieve significant effect in each condition?"
> nderrnoerr_tst <- b.studentized_ttest(tr.noerror.nd$latency, tr.error.nd$latency,
> b.showsiglev (nderrnoerr_tst, "ND no error vs ND error")
[1] "Achieved significance level for ND no error vs ND error = 0.005000"
>
> aderrnoerr_tst <- b.studentized_ttest(tr.noerror.ad$latency, tr.error.ad$latency,
> b.showsiglev (aderrnoerr_tst, "AD no error vs AD error")
[1] "Achieved significance level for AD no error vs AD error = 0.000100"
>
> sderrnoerr_tst <- b.studentized_ttest(tr.noerror.sd$latency, tr.error.sd$latency,
> b.showsiglev (sderrnoerr_tst, "SD no error vs SD error")
[1] "Achieved significance level for SD no error vs SD error < 0.000020"
>
> errnoerr.ndnd <- b.diffste(tr.error.nd$latency, tr.noerror.nd$latency, 1024)
> errnoerr.sdsd <- b.diffste(tr.error.sd$latency, tr.noerror.sd$latency, 1024)
> errnoerr.adad <- b.diffste(tr.error.ad$latency, tr.noerror.ad$latency, 1024)
>
```

1.15 Analysis of Movement Errors

It's possible to count the number of errors which the subject makes when following the target. In some trials, the subject will mistakenly begin to move towards the distractor line, before correcting (sometimes after a very short period of time) to move towards the target. I wanted to see whether the synchronous distractor or the asynchronous distractor produce more errors, but it is hard to compare. In the synchronous distractor condition, there are always the same number of distractor events as target events. In the asynchronous distractor condition, there may be more distractor events (or fewer) than target events. I concluded that it's not possible to compare SD and AD conditions.

1.15.1 Overall and Group-based movement error analysis

This R code block computes mean error rates and their MAD and StDev statistics, and it also then computes Wilcoxon signed rank test and Cliff's Delta for the ND and SD error rates. There is a bootstrap analysis of the error rates and then, in the same file, a bootstrap analysis of the fasterSync/fasterAsync sub-groups.

```
In [29]: from subprocess import CalledProcessError, check_output
         try:
             out = check_output(["R", "-q --file=Err.r"])
         except CalledProcessError as err:
             print 'Error for:',err.cmd,'with error code:',err.returncode
             print "\nOutput:\n\n",err.output
         else:
             print 'Success'
             #for line in out.splitlines():
             #    print line
```

```
Success
```

```
In [30]: print 'Bootstrapped error means'
         Image(filename='r_images/bootstrap_errors.png')
```

```
Bootstrapped error means
```

```
Out[30]:
```

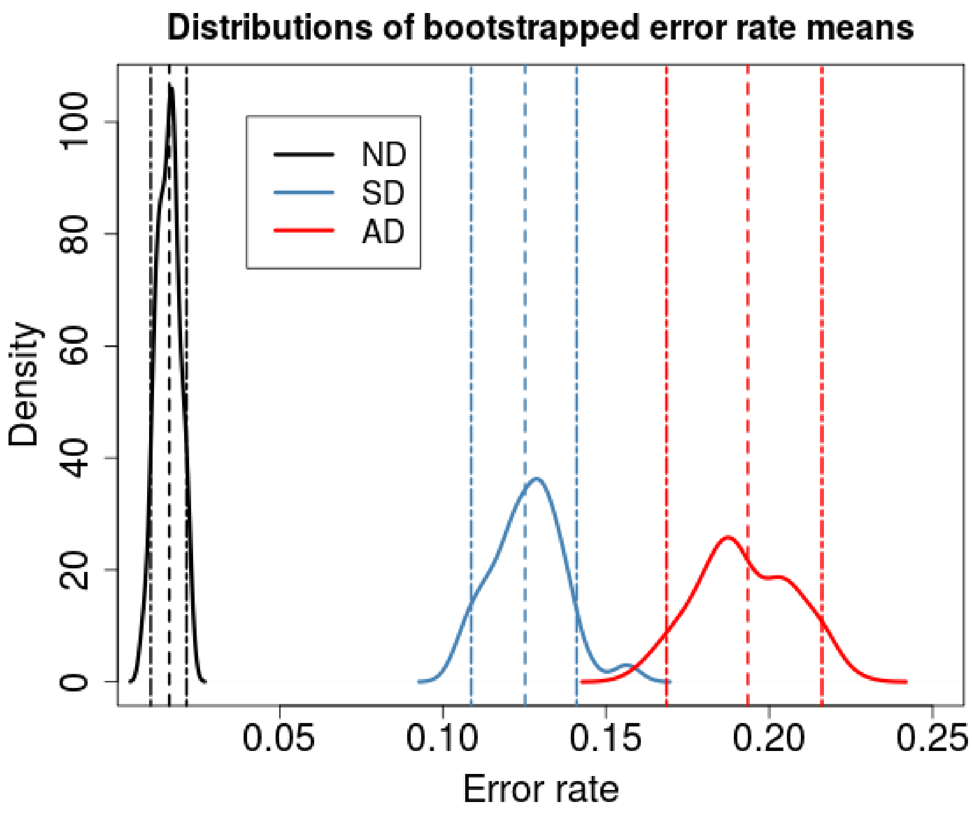


```
In [31]: from subprocess import CalledProcessError, check_output
         try:
             out = check_output(["R", "-q --file=Bootstrap_async.r"])
         except CalledProcessError as err:
             print 'Error for:',err.cmd,'with error code:',err.returncode
             print "\nOutput:\n\n",err.output
         else:
             for line in out.splitlines():
                 print line
```

```
> #
> # Analysis of Async-specific statistics. Especially:
> #
> # 1) Does last event recency affect latency of distrator movements? (Ans: No)
> # 2) Does distractor recency affect latency of target movements? (Ans: No)
> # 3) Does distractor in same direction as target reduce latency?
> # 4) What's the reaction time for distractors cf. targets?
> #
>
> maxlatency <- 1000
>
```

```
> # a function which will bootstrap the standard error of the mean
> bs.mean <- function(data, num) {
+     resamples <- lapply(1:num, function(i) sample(data, replace=T))
+     r.mean <- sapply(resamples, mean)
+     std.err <- sqrt(var(r.mean))
+     list(std.err=std.err, resamples=resamples, means=r.mean)
+ }
> bs.median <- function(data, num) {
+     resamples <- lapply(1:num, function(i) sample(data, replace=T))
+     r.median <- sapply(resamples, median)
+     std.err <- sqrt(var(r.median))
+     list(std.err=std.err, resamples=resamples, medians=r.median)
+ }
>
> d <- read.csv('AsyncTrials.csv')
>
> set.seed(197420162)
>
> # This takes a 2-D data set of latency vs time since last event. It
> # collects "bins" of data by dividing time since last event into 30
> # bins, then computes bootstrap mean & median values for each bin.
> binned <- function (data, fname) {
+     listPoints <- list()
+     listBStrap <-  list()
+     dfBStrap <- data.frame()
+     nBreaks <- 30
+     iter <- 1
+     nResamples <- 200
+     h <- hist(data$timesincelast, breaks=nBreaks, plot=F)
+     for (b in h$breaks) {
+         if (b==0) {
+             bLast = b
+             next
+         }
+
+         points <- data[which(data$timesincelast >= bLast & data$timesincelast < b
+         listPoints[[iter]] <- points
+
+         # Now bootstrap each member of listPoints, compute mean & std err of mean
+         bsmed <- bs.median(points$latency, nResamples)
+         bsmean <- bs.mean(points$latency, nResamples)
+         if (nrow(points)) {
+             dfBStrap <- rbind (dfBStrap, c((b-bLast/2), median(points$latency),
+                                            bsmed$std.err, mean(points$latency),
+                                            bsmean$std.err))
+         }
+
+         iter <- iter + 1
```

```
+         bLast = b
+     }
+     names(dfBStrap) <- c("timesincelast","median","med.stderr","mean","mean.stder
+
+     png (filename=fname)
+     plot (dfBStrap$timesincelast,dfBStrap$median, xlim=c(0,1500),# ylim=c(0,400),
+            pch=19, cex=1, cex.lab=1.5, cex.axis=1.5, cex.main=1.5, cex.sub=1.5,
+            xlab="Time since last event (ms)",ylab="Median latency (ms)", col="blac
+     lines(dfBStrap$timesincelast,dfBStrap$median, lty=1, lwd=2)
+     arrows(dfBStrap$timesincelast,dfBStrap$median-dfBStrap$med.stderr,dfBStrap$ti
+     dev.off()
+ }
>
> #
> # 1) Does last event recency affect latency of distrator movements?
> #
>
> # This selects out distractor latencies and allows me to plot the
> # latencies vs. time since last event.
> # Note: 15 is "stylus didn't move away from target" 16 is "movement occurs beyond
> dlt <- d[which (d$type == 0 & d$correctmove == 0 & d$latency<maxlatency & !(d$omi
> png (filename='./r_images/async_dist_timesince_vs_latency.png')
> plot (dlt$timesincelast,dlt$latency,xlab="Time since last event (ms)",ylab="Later
> dev.off()
null device
          1
> binned (dlt, './r_images/async_dist_binned_latencies.png')
null device
          1
>
> #
> # 2) Does distractor recency affect latency of target movements?
> #
> tlt <- d[which (d$type == 1 & d$correctmove == 1 & d$latency<maxlatency & d$omit
> png (filename='./r_images/async_targ_timesince_vs_latency.png')
> plot (tlt$timesincelast,tlt$latency)
> dev.off()
null device
          1
> binned (tlt, './r_images/async_targ_binned_latencies.png')
null device
          1
>
> # Moving bin approach for timesincelast (Question 2)
> movingbinwidth <- 80 # ms
> movingbin <- function (data) {
+     # First sort data wrt direction
+     data <- data[order(data$timesincelast),]
```

```
+     listPoints <- list()
+     listBStrap <-  list()
+     dfBStrap <- data.frame()
+     iter <- 1
+     nResamples <- 200
+     maxtime <- 1000 # ms
+     bLast <- -1
+     for (b in unique(data$timesincelast)) {
+         if (b < movingbinwidth/2) {
+             next
+         }
+         if (maxtime - b < movingbinwidth/2) {
+             next
+         }
+         points <- data[which(data$timesincelast >= b-movingbinwidth/2 & data$time
+         listPoints[[iter]] <- points
+         bsmed <- bs.median(points$latency, length(points$latency))
+         bsmean <- bs.mean(points$latency, length(points$latency))
+         if (nrow(points)) {
+             dfBStrap <- rbind (dfBStrap, c(b, median(points$latency),
+                                            bsmed$std.err, mean(points$latency),
+                                            bsmean$std.err))
+         }
+         iter <- iter + 1
+         bLast = b
+     }
+     names(dfBStrap) <- c("distance","median","med.stderr","mean","mean.stderr")
+     return (dfBStrap)
+ }
>
> amb <- movingbin (tlt)
> png (filename='./r_images/async_targ_timesince_vs_latency_movingbin.png')
> plot (amb$distance,amb$mean, xlim=c(0,1000), ylim=c(270,405),
+       pch=19, cex=0.2, cex.lab=1.5, cex.axis=1.5, cex.main=1.5, cex.sub=1.5,
+       xlab="Time since last event (ms)",ylab="Mean latency (ms)", col="white")
> lines(amb$distance,amb$mean, lty=1, lwd=3, col="red")
> lines(amb$distance,amb$mean-1.96*amb$mean.stderr, lty=1, lwd=1, col="red")
> lines(amb$distance,amb$mean+1.96*amb$mean.stderr, lty=1, lwd=1, col="red")
> dev.off()
null device
          1
>
> # A linear model shows that timesincelast does not predict latency:
> lmt <- lm(latency ~ timesincelast, data = tlt)
> print(summary(lmt))

Call:
lm(formula = latency ~ timesincelast, data = tlt)
```

```
Residuals:
    Min      1Q  Median      3Q     Max 
-246.30  -50.14   -9.82   37.51  459.08 

Coefficients:
               Estimate Std. Error t value Pr(>|t|)    
(Intercept)   3.433e+02  2.787e+00 123.141   <2e-16 ***
timesincelast 2.950e-03  3.821e-03   0.772     0.44    
---
Signif. codes:  0 '***' 0.001 '**' 0.01 '*' 0.05 '.' 0.1 ' ' 1 

Residual standard error: 81.31 on 2230 degrees of freedom
Multiple R-squared:  0.0002673,        Adjusted R-squared:  -0.000181 
F-statistic: 0.5962 on 1 and 2230 DF,  p-value: 0.4401 

> 
> # 
> # 3) Does distractor in same direction as target reduce latency?
> # 
> 
> # A graphing function for target data and distractor data, both
> # assumed to be simple vectors of data
> bs_graph <- function(dat1, dat2, dat1label, dat2label, xrange, yrange, alpha, mea
+ 
+     densityN <- 256
+     nResamples <- 1024
+     dat1Col <- "steelblue"
+     dat2Col <- "red"
+ 
+     # Raw distributions
+     dat1dens = density(dat1, n=512)
+     dat1densScale = max(dat1dens$y)
+     dat2dens = density(dat2, n=512)
+     dat2densScale = max(dat2dens$y)
+     xmax = max(dat1dens$x,dat2dens$x)
+     xmin = min(dat1dens$x,dat2dens$x)
+ 
+     if (xrange[2]==0) {
+         xrange <- c(xmin,xmax)
+     }
+ 
+     if (mean == TRUE) {
+         bsdat1 <- bs.mean(dat1, nResamples)
+         q5 <- quantile(bsdat1$means,alpha)
+         q95 <- quantile(bsdat1$means,1-alpha)
+         df <- density(bsdat1$means, n=densityN)
+         cent <- mean(dat1)
```

```
+           maintitle <- sprintf("Distns of bootstrapped means. alpha=%.3f", alpha)
+           bsdat2 <- bs.mean(dat2, nResamples)
+           df_ <- density(bsdat2$means, n=densityN)
+           centtype <- 'mean'
+       } else {
+           bsdat1 <- bs.median(dat1, nResamples)
+           q5 <- quantile(bsdat1$medians,alpha)
+           q95 <- quantile(bsdat1$medians,1-alpha)
+           df <- density(bsdat1$medians, n=densityN)
+           cent <- median(dat1)
+           maintitle <- sprintf("Distns of bootstrapped medians. alpha=%.3f", alpha)
+           bsdat2 <- bs.median(dat2, nResamples)
+           df_ <- density(bsdat2$medians, n=densityN)
+           centtype <- 'median'
+       }
+
+       print (sprintf('%s: %s = %f +- %f', dat1label, centtype, cent, q95-cent))
+       print (sprintf('%s: std %f, mad: %f', dat1label, sd(dat1), mad(dat1)))
+
+       if (yrange[2]==0) {
+           yrange <- c(0,max(df$y,df_$y))
+       }
+
+       # Plot dat1 first
+       plot(df, lwd=2, col=dat1Col, xlim=xrange, ylim=yrange,
+            cex.lab=1.5, cex.axis=1.5, cex.main=1.5, cex.sub=1.5,
+            main=maintitle, xlab="Latency (ms)")
+       abline(v=cent, lty=2, lwd=2, col=dat1Col)
+       abline(v=q5[1], lty=6, lwd=2, col=dat1Col)
+       abline(v=q95[1], lty=6, lwd=2, col=dat1Col)
+
+       dat1densScale =  max(df$y) / dat1densScale
+       print (dat1densScale)
+       lines(dat1dens$x, dat1dens$y * dat1densScale, lwd=2, lty=5, col=dat1Col)
+
+
+
+       if (mean == TRUE) {
+           q5 <- quantile(bsdat2$means,alpha)
+           q95 <- quantile(bsdat2$means,1-alpha)
+           df <- density(bsdat2$means, n=densityN)
+           cent <- mean(dat2)
+       } else {
+           q5 <- quantile(bsdat2$medians,alpha)
+           q95 <- quantile(bsdat2$medians,1-alpha)
+           df <- density(bsdat2$medians, n=densityN)
+           cent <- median(dat2)
+       }
```

```
+
+     print (sprintf('%s: %s = %f +- %f', dat2label, centtype, cent, q95-cent))
+     print (sprintf('%s: std %f, mad: %f', dat2label, sd(dat2), mad(dat2)))
+
+     # Now plot dat2
+     lines(df, lwd=2, col=dat2Col,
+           cex.lab=1.5, cex.axis=1.5, cex.main=1.5, cex.sub=1.5,
+           main=maintitle, xlab="Latency (ms)")
+     abline(v=cent, lty=2, lwd=2, col=dat2Col)
+     abline(v=q5[1], lty=6, lwd=2, col=dat2Col)
+     abline(v=q95[1], lty=6, lwd=2, col=dat2Col)
+
+     dat2densScale =  max(df$y) / dat2densScale
+     lines(dat2dens$x, dat2dens$y * dat2densScale, lwd=2, lty=5, col=dat2Col)
+
+     legend ("topleft", c(dat1label, dat2label),
+             lty=c(1,1),
+             lwd=c(2,2),
+             col=c(dat1Col,dat2Col)
+             )
+     legend ("topright", c('bootstrap distribution','raw distn (scaled)',centtype,
+             lty=c(1,5,2,6),
+             lwd=c(2,2,2,2),
+             col=c(dat1Col,dat1Col,dat1Col,dat1Col)
+             )
+
+ }
>
> # Get filenames:
> fns <- list.files(pattern="AsyncDat*")
> # Create container
> d2 <- data.frame()
> for (indiv in fns) {
+     dat <- read.csv(indiv)
+     # Add the 'opposite' column:
+     dat$opposite <- 0
+     # From this individual's data, find those targets for which prev. distractor
+     for (i in 1:nrow(dat)) {
+         if (dat[i,]$type == 1.0) {
+             # 1 is TARG_EVENT
+             earlier <- dat[which (dat$type == 0 & dat$num < i),]
+             if (nrow(earlier)) {
+                 # Got earlier events, now find most recent earlier
+                 # event and extract its destination. Compare this with
+                 # the target destination.
+                 dest_d <- earlier[earlier$num==max(earlier$num),]$destination
+                 dest_t <- dat[i,]$destination
+                 dir_t <- dat[i,]$direction
```

```
+                 start <- dest_t - dir_t
+                 if ((dest_d < start & dest_t > start)
+                     | (dest_d > start & dest_t < start)) {
+                     # targ and dist in opposite directions
+                     dat[i,]$opposite=1
+                 } else {
+                     # targ and dist NOT in opposite directions
+                 }
+
+             }
+         }
+     }
+     # Lastly? combine data frame with the others to make up a return
+     # data frame from which I can determine if opposite makes a
+     # difference.
+     d2 <- rbind(d2,dat)
+ }
> # Extract opposite and same-side data:
> tlt_opp <- d2[which (d2$type == 1 & d2$opposite == 1 & d2$correctmove == 1 & d2$l
> tlt_same <- d2[which (d2$type == 1 & d2$opposite == 0 & d2$correctmove == 1 & d2$
> # Now make a nice graph:
> png(filename='./r_images/async_targ_vs_oppositeness_of_distractor.png')
> xrange <- c(325,360)
> yrange <- c(0,0)
> bs_graph(tlt_opp, tlt_same, "distractor opposite", "distractor same", xrange, yra
[1] "distractor opposite: median = 330.000000 +- 5.500000"
[1] "distractor opposite: std 83.191973, mad: 60.786600"
[1] 26.33083
[1] "distractor same: median = 336.000000 +- 4.000000"
[1] "distractor same: std 79.961187, mad: 65.234400"
> dev.off()
null device
          1
> # This appears to show that it is likely (0.79 probability) that the
> # "distractor opposite" does have a small effect, increasing the
> # latency by about 5 ms. However, there's a 0.1958 probability that
> # the "distractor opposite" makes *no difference* to the latency and a
> # 0.0121 probability that the "distractor opposite" actually decreases
> # the latency. A 0.2 probability of no effect/the means being opposite
> # doesn't pass the usual 0.05 alpha test, so conclude that this is
> # still non-significant.
>
>
> #
> # 4) What's the reaction time for distractors cf. targets?
> #
> png(filename='./r_images/async_targ_vs_dist.png')
> xrange <- c(180,500)
```

```
> yrange <- c(0,0)
> print('Async mean')
[1] "Async mean"
> bs_graph (tlt$latency, dlt$latency, "target latency","distractor latency", xrange
[1] "target latency: mean = 344.943996 +- 5.098332"
[1] "target latency: std 81.303108, mad: 65.234400"
[1] 37.57107
[1] "distractor latency: mean = 270.267241 +- 16.447305"
[1] "distractor latency: std 95.227925, mad: 57.821400"
> dev.off()
null device
          1
> png(filename='./r_images/async_targ_vs_dist_median.png')
> xrange <- c(180,500)
> yrange <- c(0,0)
> print('Async median')
[1] "Async median"
> bs_graph (tlt$latency, dlt$latency, "target latency","distractor latency", xrange
[1] "target latency: median = 335.000000 +- 5.000000"
[1] "target latency: std 81.303108, mad: 65.234400"
[1] 225.7886
[1] "distractor latency: median = 269.000000 +- 12.000000"
[1] "distractor latency: std 95.227925, mad: 57.821400"
> dev.off()
null device
          1
>
>
> #
> # Last - sync trials.
> #
> d <- read.csv('SyncTrials.csv')
> set.seed(197420163)
> # distracted latencies. correctmove is 0 when definitely incorrect, -1 when undet
> dlt <- d[which (d$correctmove == 0 & d$latency<maxlatency & d$latency>0),]$latenc
> tlt <- d[which (d$correctmove == 1 & d$latency<maxlatency & d$latency>0),]$latenc
> png(filename='./r_images/sync_targ_vs_dist.png')
> xrange <- c(150,550)
> yrange <- c(0,0)
> print('Sync mean')
[1] "Sync mean"
> bs_graph (tlt, dlt, "target latency","distractor latency", xrange, yrange, 0.001,
[1] "target latency: mean = 334.104204 +- 4.165407"
[1] "target latency: std 60.492715, mad: 51.891000"
[1] 39.15872
[1] "distractor latency: mean = 263.697248 +- 11.959401"
[1] "distractor latency: std 78.343373, mad: 51.891000"
> dev.off()
```

```
null device
          1
>
> png(filename='./r_images/sync_targ_vs_dist_median.png')
> xrange <- c(150,550)
> yrange <- c(0,.33)
> print('Sync median')
[1] "Sync median"
> bs_graph (tlt, dlt, "target latency","distractor latency", xrange, yrange, 0.001,
[1] "target latency: median = 330.000000 +- 5.000000"
[1] "target latency: std 60.492715, mad: 51.891000"
[1] 92.50053
[1] "distractor latency: median = 265.000000 +- 14.977000"
[1] "distractor latency: std 78.343373, mad: 51.891000"
> dev.off()
null device
          1
>
>
> #
> # REALLY Last - nodist trials.
> #
> d <- read.csv('NoDistTrials.csv')
> set.seed(197420166)
> # distracted latencies. correctmove is 0 when definitely incorrect, -1 when undet
> dlt <- d[which (d$correctmove == 0 & d$latency<maxlatency & d$latency>100),]$late
> tlt <- d[which (d$correctmove == 1 & d$latency<maxlatency & d$latency>100),]$late
> png(filename='./r_images/nodist_targ_vs_dist.png')
> xrange <- c(80,550)
> yrange <- c(0,0)
> print('Nodist mean')
[1] "Nodist mean"
> bs_graph (tlt, dlt, "target latency","distractor latency", xrange, yrange, 0.01,
[1] "target latency: mean = 294.902810 +- 2.421714"
[1] "target latency: std 51.334525, mad: 44.478000"
[1] 41.66836
[1] "distractor latency: mean = 229.057143 +- 50.083714"
[1] "distractor latency: std 127.433707, mad: 106.747200"
> dev.off()
null device
          1
>
> png(filename='./r_images/nodist_targ_vs_dist_median.png')
> xrange <- c(80,550)
> yrange <- c(0,0)
> print('Nodist median')
[1] "Nodist median"
> bs_graph (tlt, dlt, "target latency","distractor latency", xrange, yrange, 0.01,
```

```
[1] "target latency: median = 290.000000 +- 1.000000"
[1] "target latency: std 51.334525, mad: 44.478000"
[1] 221.3067
[1] "distractor latency: median = 204.000000 +- 44.250000"
[1] "distractor latency: std 127.433707, mad: 106.747200"
> dev.off()
null device
          1
>
```

```
In [32]: print '1) Does last event recency affect latency of DISTRACTOR movements?
         print 'Raw distractor latency vs. time-since-last-event:'
         Image(filename='r_images/async_dist_timesince_vs_latency.png')
```

```
1) Does last event recency affect latency of DISTRACTOR movements? ANS: No.
Raw distractor latency vs. time-since-last-event:
```

```
Out[32]:
```

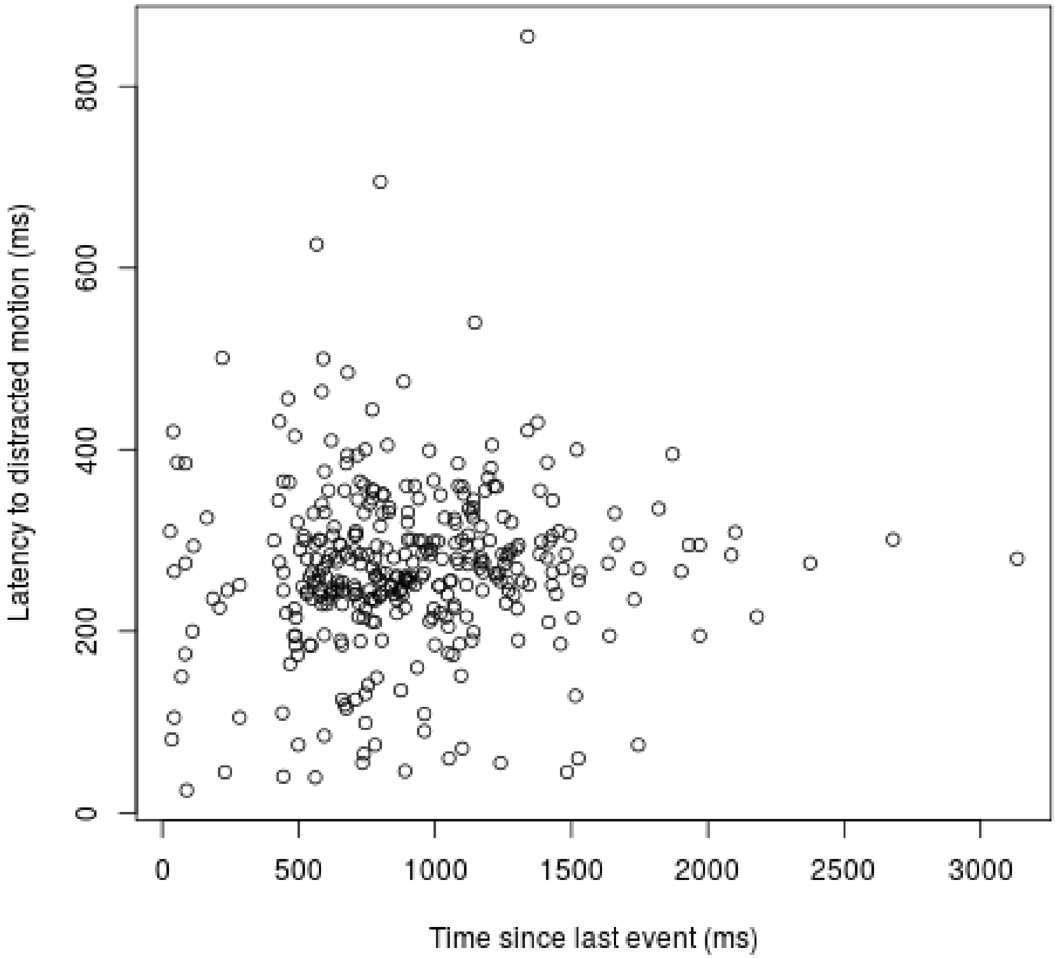


```
In [33]: print 'Binned median latencies'
         Image(filename='./r_images/async_dist_binned_latencies.png')

Binned median latencies


Out[33]:
```

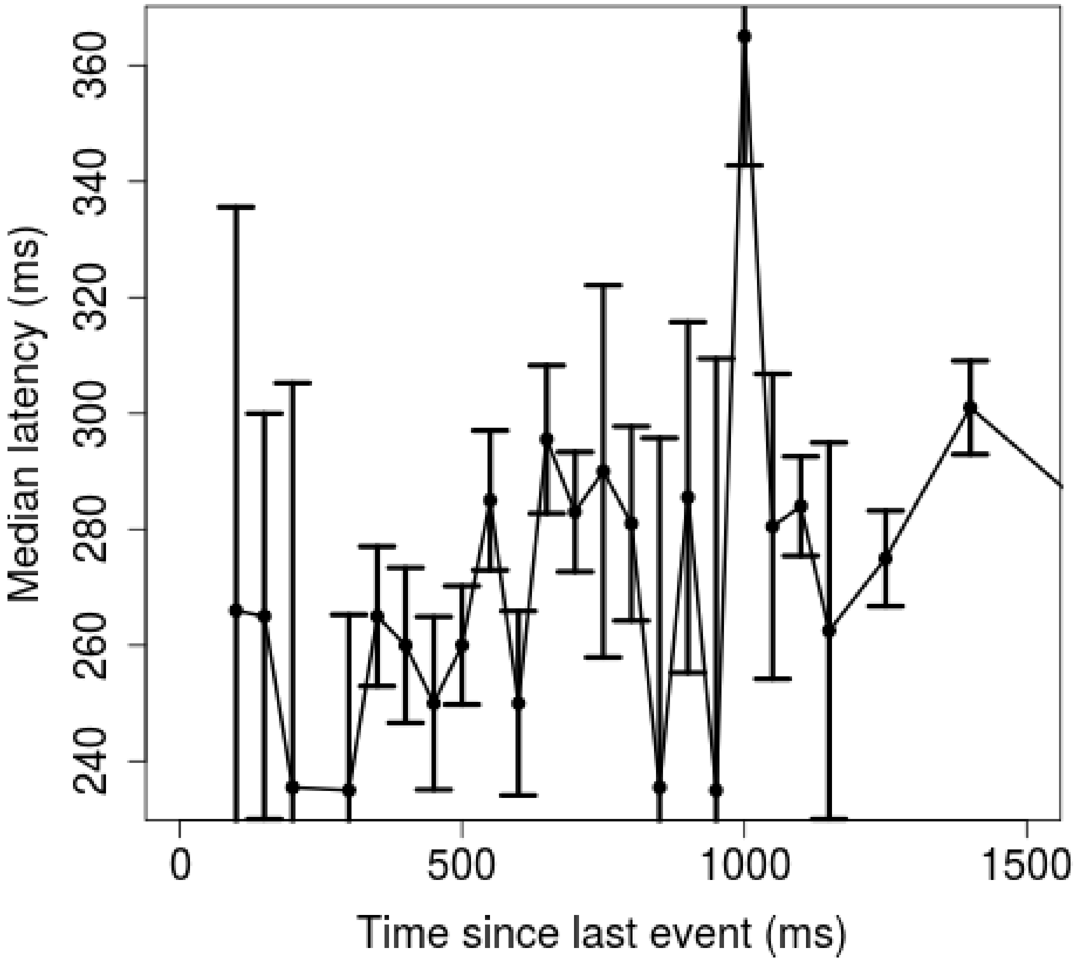


```
In [34]: print '2) Does last event recency affect latency of TARGET movements? ANS:
         print 'Raw target latency vs. time-since-last-event:'
         Image(filename='r_images/async_targ_timesince_vs_latency.png')
```

```
2) Does last event recency affect latency of TARGET movements? ANS: No.
Raw target latency vs. time-since-last-event:
```

Out[34]:

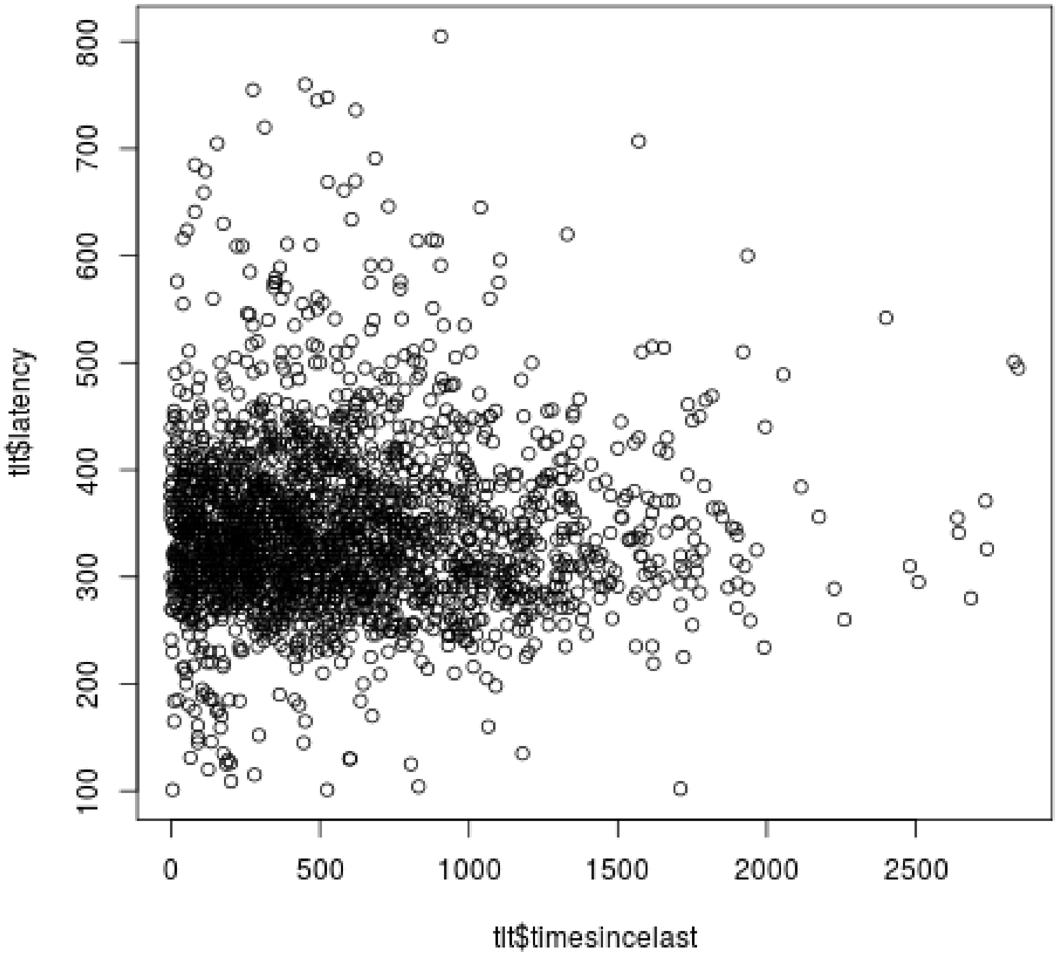


```
In [35]: print 'Binned median latencies'
         Image(filename='./r_images/async_targ_binned_latencies.png')
```

```
Binned median latencies
```

Out[35]:

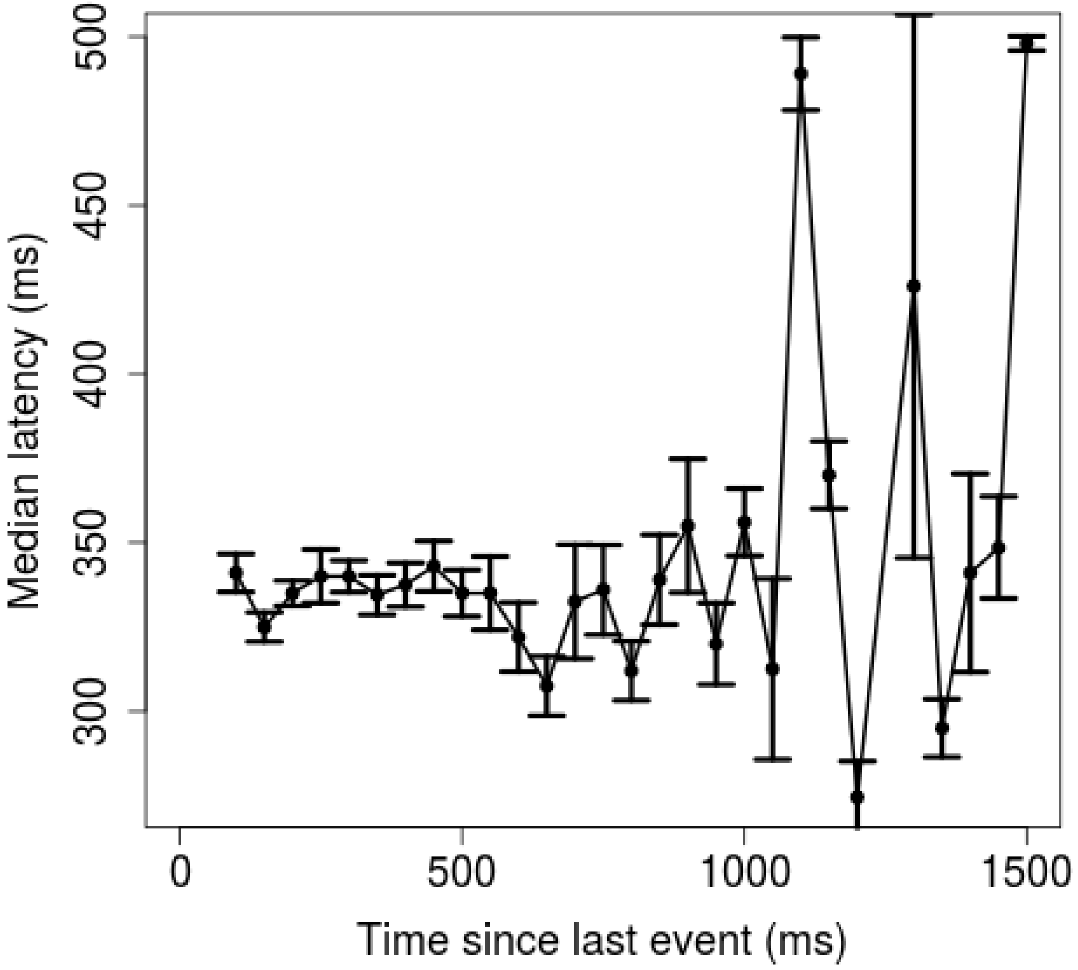


```
In [36]: print '3) Does distractor in same direction as target reduce latency? ANS:
         print 'Here "distractor opposite" means:\n"the existing distractor locatio
         print "Can say that: There's 0.7921 probability that lat_dist_same > lat_d
         print "probability that lat_dist_same is not determinedly > or < lat_dist_
         print "there's 0.0121 probability that lat_dist_same < lat_dist_opp."
         Image(filename='./r_images/async_targ_vs_oppositeness_of_distractor.png')
```

```
3) Does distractor in same direction as target reduce latency? ANS: No (not signifi
Here "distractor opposite" means:
"the existing distractor location was in the opposite direction compared with the n
Can say that: There's 0.7921 probability that lat_dist_same > lat_dist_opp; 0.1958
probability that lat_dist_same is not determinedly > or < lat_dist_opp. Finally,
there's 0.0121 probability that lat_dist_same < lat_dist_opp.
```

Out[36]:

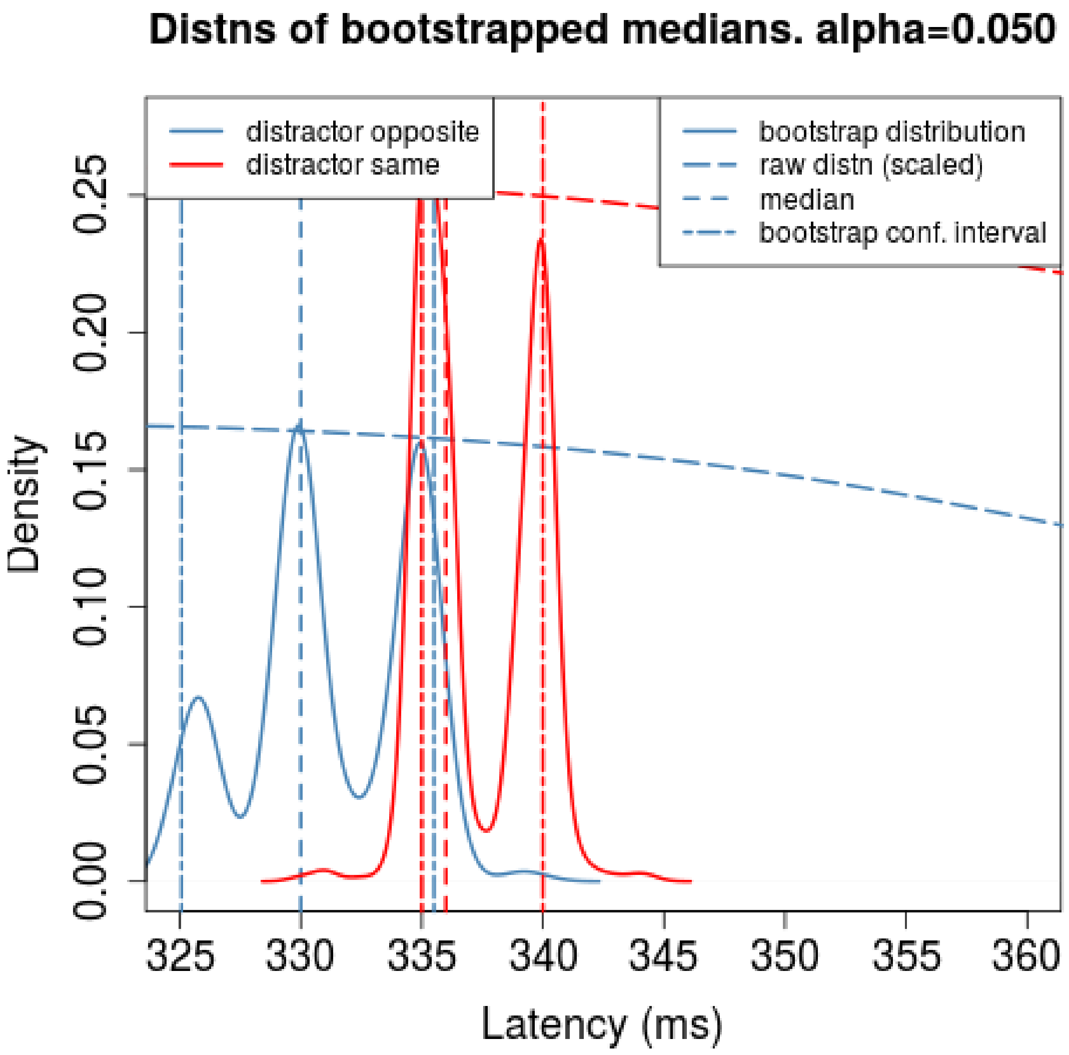


```
In [37]: print '4) What\'s the reaction time for distractors cf. targets *within th
         Image(filename='./r_images/async_targ_vs_dist.png')
```

```
4) What's the reaction time for distractors cf. targets *within the AD condition*?
ANS: Significantly different.
```

Out[37]:

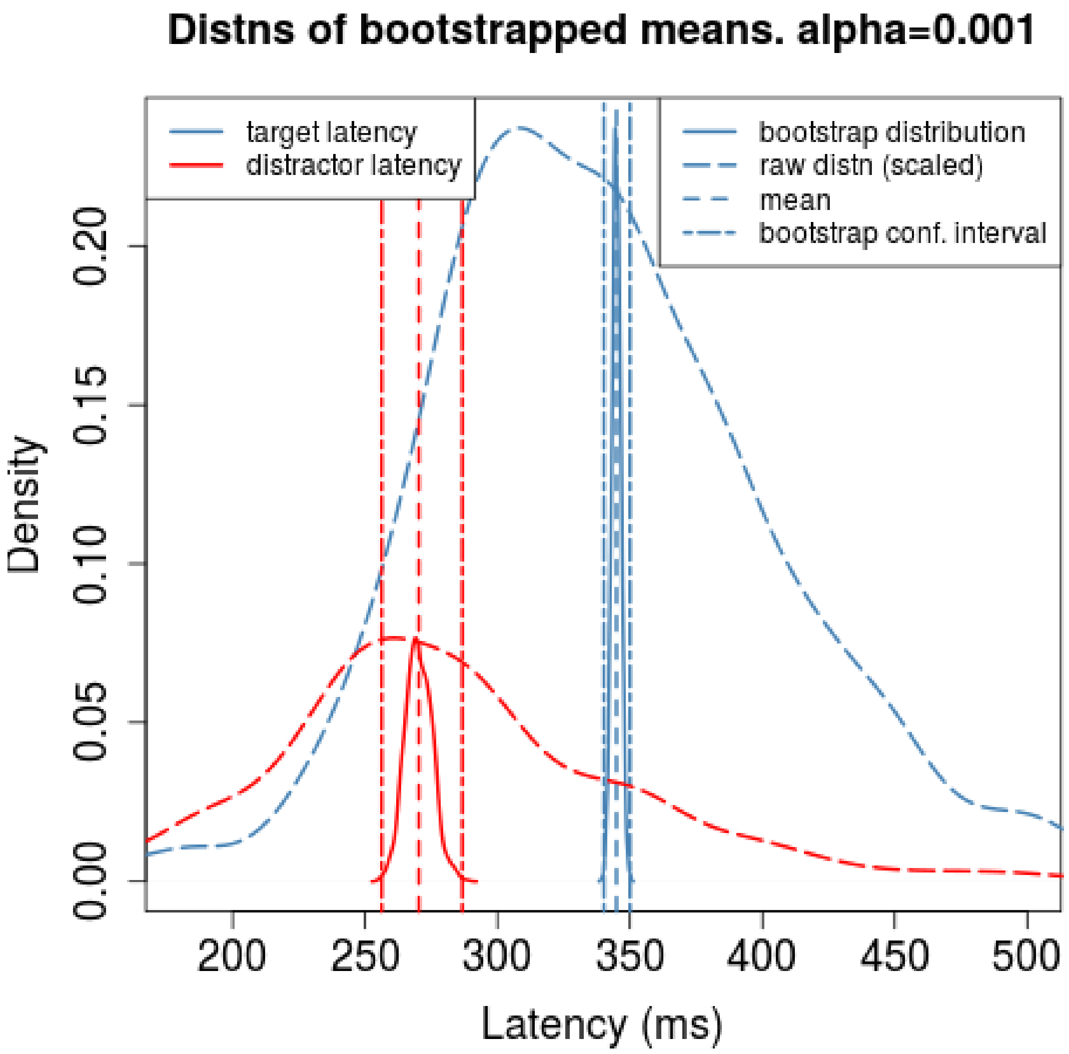


```
In [38]: print '5) What\'s the reaction time for distractors cf. targets *within th
         Image(filename='./r_images/sync_targ_vs_dist.png')
```

```
5) What's the reaction time for distractors cf. targets *within the SD condition*?
```

Out[38]:

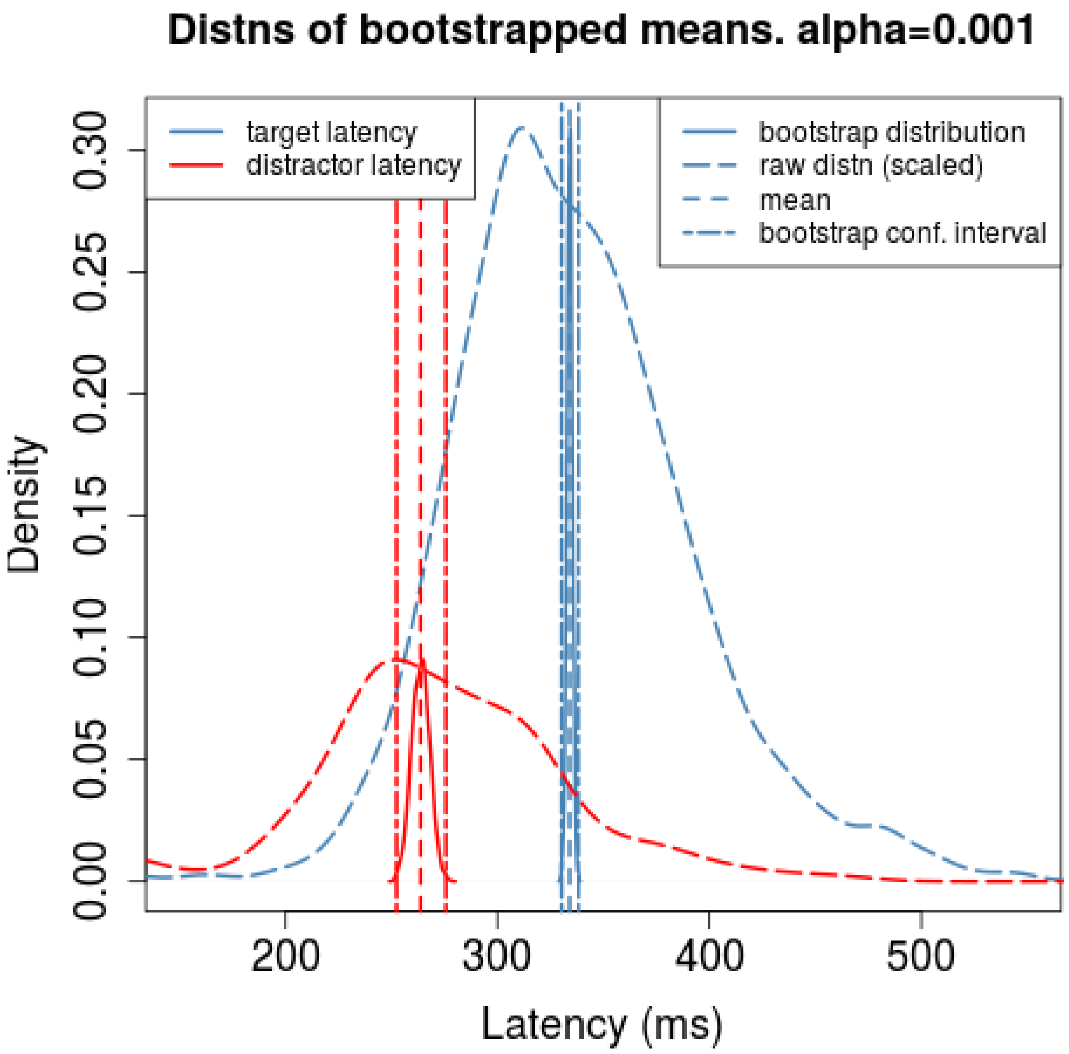


```
In [39]: print '6) What\'s the reaction time for error moves cf. correct, target mo
         Image(filename='./r_images/nodist_targ_vs_dist.png')
```

```
6) What's the reaction time for error moves cf. correct, target movements *within t
```

```
Out[39]:
```

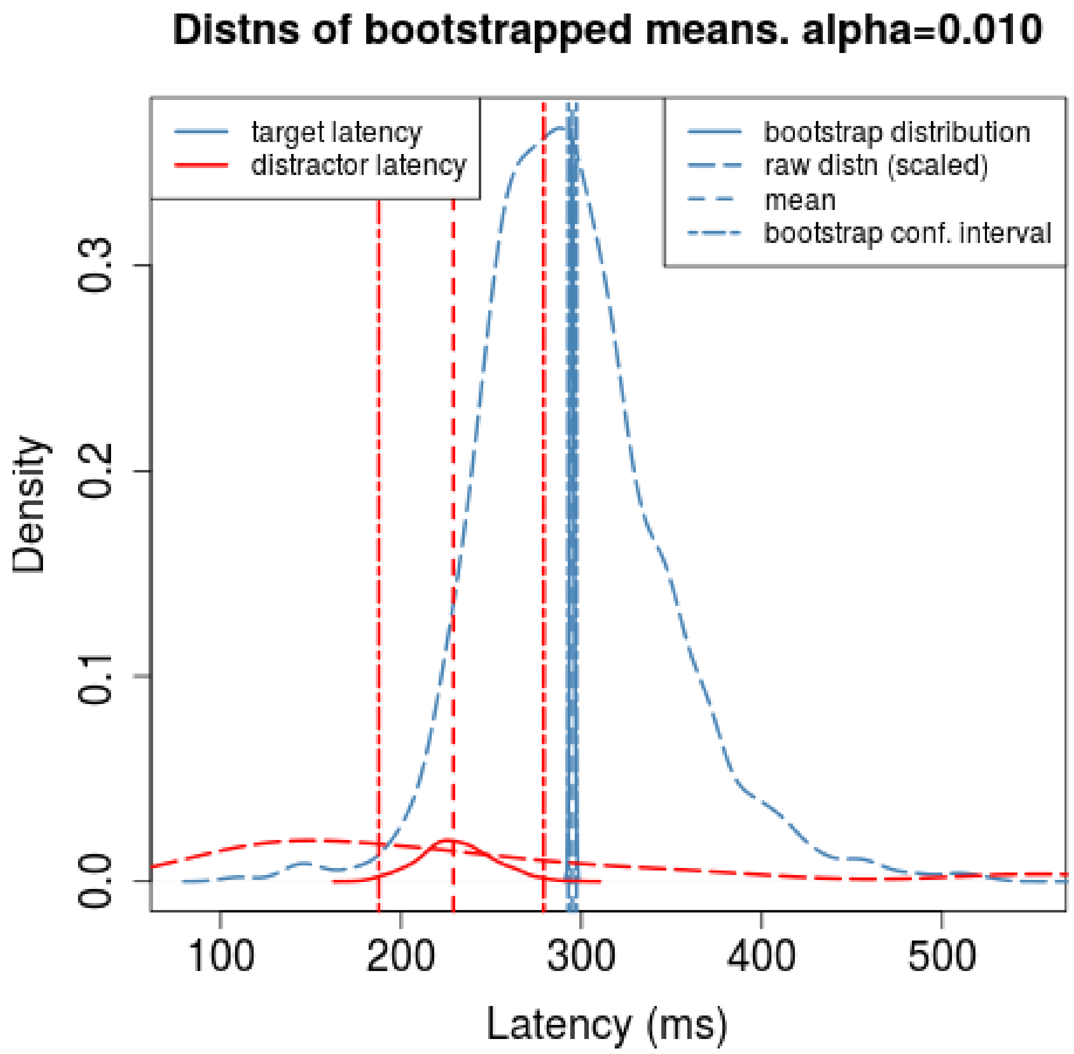

1.16 Analysis of latencies wrt target distance

This code (Bootstrap_targdist.r) looks at how the latency to first movement varies with distance to the target, and also wst distance to the last distractor.

```
In [40]: from subprocess import CalledProcessError, check_output
         try:
             out = check_output(["R", "-q --file=Bootstrap_targdist.r"])
         except CalledProcessError as err:
             print 'Error for:',err.cmd,'with error code:',err.returncode
             print "\nOutput:\n\n",err.output
         else:
             for line in out.splitlines():
                 print line
```

```
> ##
> ## Analysis of distance-to-target statistics.
> ## ALSO analysis of distance-to-distractor stats.
> ##
> ## 1) Does the distance to the target have an effect on the latency?
> ## This has been reported by Meegan & Tipper 99, Pratt and Abrams 94,
> ## Tipper et al 92 and 97.
> ##
>
> maxlatency <- 1000
>
> set.seed(197420168)
>
> ## a function which will bootstrap the standard error of the mean
> bs.mean <- function(data, num) {
+     resamples <- lapply(1:num, function(i) sample(data, replace=T))
+     r.mean <- sapply(resamples, mean)
+     std.err <- sqrt(var(r.mean))
+     list(std.err=std.err, resamples=resamples, means=r.mean)
+ }
> bs.median <- function(data, num) {
+     resamples <- lapply(1:num, function(i) sample(data, replace=T))
+     r.median <- sapply(resamples, median)
+     std.err <- sqrt(var(r.median))
+     list(std.err=std.err, resamples=resamples, medians=r.median)
+ }
>
> ## This takes a 2-D data set of latency vs time since last event. It
> ## collects "bins" of data by dividing time since last event into 30
> ## bins, then computes bootstrap mean & median values for each bin.
> binned <- function (data, fname) {
+     listPoints <- list()
+     listBStrap <-  list()
+     dfBStrap <- data.frame()
+     nBreaks <- 30
+     iter <- 1
+     nResamples <- 200
+     h <- hist(abs(data$direction), breaks=nBreaks, plot=F)
+     bLast <- -1
+     for (b in h$breaks) {
+         if (b==0) {
+             bLast = b
+             next
+         }
+
+         points <- data[which(abs(data$direction) >= bLast & abs(data$direction) <
+         listPoints[[iter]] <- points
+
```

```
+          ## Now bootstrap each member of listPoints, compute mean & std err of mea
+          bsmed <- bs.median(points$latency, nResamples)
+          bsmean <- bs.mean(points$latency, nResamples)
+
+          if (nrow(points)) {
+              dfBStrap <- rbind (dfBStrap, c((b-bLast/2), median(points$latency),
+                                             bsmed$std.err, mean(points$latency),
+                                             bsmean$std.err))
+          }
+
+          iter <- iter + 1
+          bLast = b
+      }
+      names(dfBStrap) <- c("distance","median","med.stderr","mean","mean.stderr")
+      return (dfBStrap)
+ }
>
> movingbinwidth <- 44 ## 44 corresponds to about 5 mm of screen; 1/7 foveal width
>
> movingbin <- function (data, fname, distance) {
+
+      ## First sort data wrt distance (absolute value of direction)
+      ## data$distance <- abs(data$direction) # moved outside.
+      data$distance <- distance
+      data <- data[order(data$distance),]
+
+      listPoints <- list()
+      listBStrap <-  list()
+      dfBStrap <- data.frame()
+      iter <- 1
+      nResamples <- 200
+      maxdist <- max(data$distance)
+      bLast <- -1
+      for (b in unique(data$distance)) {
+
+          if (b < movingbinwidth/2) {
+              next
+          }
+
+          if (maxdist - b < movingbinwidth/2) {
+              next
+          }
+
+          points <- data[which(data$distance >= b-movingbinwidth/2 & data$distance
+
+          listPoints[[iter]] <- points
+
+          ## Now bootstrap each member of listPoints, compute mean & std err of mea
```

```
+         bsmed <- bs.median(points$latency, length(points$latency))
+         bsmean <- bs.mean(points$latency, length(points$latency))
+
+         if (nrow(points)) {
+             ## 8.80734 mm per pixel, so this will make distance in mm.
+             dfBStrap <- rbind (dfBStrap, c(b/8.80734, median(points$latency),
+                                            bsmed$std.err, mean(points$latency),
+                                            bsmean$std.err))
+         }
+
+         iter <- iter + 1
+         bLast = b
+     }
+     names(dfBStrap) <- c("distance","median","med.stderr","mean","mean.stderr")
+
+     return (dfBStrap)
+ }
>
> movingbindir <- function (data, fname) {
+
+     ## First sort data wrt direction
+     data <- data[order(data$direction),]
+
+     listPoints <- list()
+     listBStrap <-  list()
+     dfBStrap <- data.frame()
+     iter <- 1
+     nResamples <- 200
+     maxdist <- max(data$direction)
+     mindist <- min(data$direction)
+     bLast <- -1
+     for (b in unique(data$direction)) {
+
+         if (b < mindist + movingbinwidth/2) {
+             next
+         }
+
+         if (maxdist - b < movingbinwidth/2) {
+             next
+         }
+
+         points <- data[which(data$direction >= b-movingbinwidth/2 & data$directio[illegible]
+
+         listPoints[[iter]] <- points
+
+         # Now bootstrap each member of listPoints, compute mean & std err of mea[illegible]
+         bsmed <- bs.median(points$latency, length(points$latency))
+         bsmean <- bs.mean(points$latency, length(points$latency))
```

```
+
+         if (nrow(points)) {
+             # 8.80734 mm per pixel, so this will make distance in mm.
+             dfBStrap <- rbind (dfBStrap, c(b/8.80734, median(points$latency),
+                                            bsmed$std.err, mean(points$latency),
+                                            bsmean$std.err))
+         }
+
+         iter <- iter + 1
+         bLast = b
+     }
+     names(dfBStrap) <- c("distance","median","med.stderr","mean","mean.stderr")
+
+     return (dfBStrap)
+ }
>
>
> ##
> ## 1) Does event destination magnitude affect latency of target movements?
> ##
>
> ## This selects out target latencies and allows me to plot the
> ## latencies vs. distance to target (magnitude of direction)
> d <- read.csv('AsyncTrials.csv')
> alt <- d[which (d$type == 1 & d$latency > 0 & d$correctmove == 1 & d$omit == 0 ),
> png (filename='./r_images/async_latency_vs_dist.png')
> plot (abs(alt$direction),alt$latency,xlab="Distance to target",ylab="Latency to m
> dev.off()
null device
          1
>
> d <- read.csv('SyncTrials.csv')
> slt <- d[which (d$type == 1 & d$latency > 0 & d$correctmove == 1 & d$omit == 0 ),
> png (filename='./r_images/sync_latency_vs_dist.png')
> plot (abs(slt$direction),slt$latency,xlab="Distance to target",ylab="Latency to m
> dev.off()
null device
          1
>
> d <- read.csv('NoDistTrials.csv')
> nlt <- d[which (d$type == 1 & d$latency > 0 & d$correctmove == 1 & d$omit == 0 ),
> png (filename='./r_images/nodist_latency_vs_dist.png')
> plot (abs(nlt$direction),nlt$latency,xlab="Distance to target",ylab="Latency to m
> dev.off()
null device
          1
>
> ## Compute moving bootstraps
```

```
> distance <- abs(alt$direction)
> amb <- movingbin (alt, './r_images/async_targ_binned_latvsdist.png', distance)
> distance <- abs(slt$direction)
> smb <- movingbin (slt, './r_images/sync_targ_binned_latvsdist.png', distance)
> distance <- abs(nlt$direction)
> nmb <- movingbin (nlt, './r_images/nodist_targ_binned_latvsdist.png', distance)
>
> ## A common plotting function
> plotfn <- function (nmb, smb, amb, xlimits) {
+     plot (amb$distance,amb$mean, xlim=xlimits, ylim=c(270,405),
+            pch=19, cex=0.2, cex.lab=1.5, cex.axis=1.5, cex.main=1.5, cex.sub=1.5,
+            xlab="Distance to target (mm)",ylab="Mean latency (ms)", col="white")
+
+     lines(amb$distance,amb$mean, lty=1, lwd=3, col="red")
+     lines(amb$distance,amb$mean-1.96*amb$mean.stderr, lty=1, lwd=1, col="red")
+     lines(amb$distance,amb$mean+1.96*amb$mean.stderr, lty=1, lwd=1, col="red")
+
+     lines(smb$distance,smb$mean, lty=1, lwd=3, col="blue")
+     lines(smb$distance,smb$mean-1.96*smb$mean.stderr, lty=1, lwd=1, col="blue")
+     lines(smb$distance,smb$mean+1.96*smb$mean.stderr, lty=1, lwd=1, col="blue")
+
+     lines(nmb$distance,nmb$mean, lty=1, lwd=3, col="black")
+     lines(nmb$distance,nmb$mean-1.96*nmb$mean.stderr, lty=1, lwd=1, col="black")
+     lines(nmb$distance,nmb$mean+1.96*nmb$mean.stderr, lty=1, lwd=1, col="black")
+
+     lines(c(11.9,11.9),c(270,400),lty=2, lwd=2, col='steelblue')
+
+     text (7,280, sprintf('Bin width: %.1f mm', movingbinwidth/8.807))
+
+     legend (20, 408, bg="white",
+             c("No distractor","Synchronous distractor","Asynchronous distractor",
+             lty=c(1,1,1,2),
+             lwd=c(3,3,3,2),
+             cex=1.0,
+             col=c('black','blue','red','steelblue'))
+ }
>
> ## Do the png plot
> png (filename='./r_images/movingbin_latvsdist.png')
> plotfn(nmb,smb,amb,c(0,55))
> dev.off()
null device
          1
>
> ## And the eps plot
> setEPS()
> postscript(file='../paper/figures/movingbin_latvsdist.eps',width=8,height=7)
> plotfn(nmb,smb,amb,c(0,55))
```

```
> dev.off()
null device
          1
>
> ##
> ## 2) This does the same analysis as above (latency vs. distance to
> ## target), but without throwing away direction information.
> ##
> ambdir <- movingbindir (alt, './r_images/async_targ_binned_latvsdir.png')
> smbdir <- movingbindir (slt, './r_images/sync_targ_binned_latvsdir.png')
> nmbdir <- movingbindir (nlt, './r_images/nodist_targ_binned_latvsdir.png')
> png (filename='./r_images/movingbin_latvsdir.png')
> plotfn(nmbdir,smbdir,ambdir,c(-55,55))
> dev.off()
null device
          1
>
> ##
> ## 3) Latency vs. distance to last distractor
> ##
> d <- read.csv('AsyncTrials.csv')
> ald <- d[which (d$type == 1 & d$latency > 0 & d$correctmove == 1 & d$omit == 0 ),
> png (filename='./r_images/async_latency_vs_lastdist.png')
> plot (abs(ald$last_distractor_offset),ald$latency,xlab="Distance to last distract
> dev.off()
null device
          1
> distance <- abs(ald$last_distractor_offset)
> amb2 <- movingbin (ald, './r_images/async_targ_binned_latvsdist2.png', distance)
>
> ## A copy of plotfn() specifically for the latency to distractor.
> plotfn2 <- function (aldmb, altmb, xlimits) {
+     plot (altmb$distance, altmb$mean, xlim=xlimits, ylim=c(270,405),
+           pch=19, cex=0.2, cex.lab=1.5, cex.axis=1.5, cex.main=1.5, cex.sub=1.5,
+           xlab="Distance (mm)", ylab="Mean latency (ms)", col="white")
+
+     lines(altmb$distance,altmb$mean, lty=1, lwd=3, col="red")
+     lines(altmb$distance,altmb$mean-1.96*altmb$mean.stderr, lty=1, lwd=1, col="re
+     lines(altmb$distance,altmb$mean+1.96*altmb$mean.stderr, lty=1, lwd=1, col="re
+
+     lines(aldmb$distance,aldmb$mean, lty=1, lwd=3, col="seagreen3")
+     lines(aldmb$distance,aldmb$mean-1.96*aldmb$mean.stderr, lty=1, lwd=1, col="se
+     lines(aldmb$distance,aldmb$mean+1.96*aldmb$mean.stderr, lty=1, lwd=1, col="se
+
+     lines(c(11.9,11.9),c(270,400),lty=2, lwd=2, col='steelblue')
+
+     text (80,400, sprintf('Bin width: %.1f mm', movingbinwidth/8.807))
+
```

```
+     legend (40, 300, bg="white",
+             c("vs. distance to last distractor","vs. distance to target","Approx.
+             lty=c(1,1,2),
+             lwd=c(3,3,2),
+             cex=1.0,
+             col=c('seagreen3','red','steelblue'))
+ }
>
> png (filename='./r_images/movingbin_latvslastdist_async.png')
> plotfn2(amb2,amb,c(0,105))
> dev.off()
null device
          1
>
> setEPS()
> postscript(file='../paper/figures/movingbin_latvslastdist_async.eps',width=8,heig
> plotfn2(amb2,amb,c(0,105))
> dev.off()
null device
          1
>
> ## Quick linear regression model for latency vs. distance to last distractor
> ## This gives F(1,2230) = 2.799, p=0.094
> lm1 <- lm(ald$latency ~ ald$last_distractor_offset)
> summary(lm1)

Call:
lm(formula = ald$latency ~ ald$last_distractor_offset)

Residuals:
    Min      1Q  Median      3Q     Max
-245.52  -50.61   -9.77   38.14  459.76

Coefficients:
                             Estimate Std. Error t value Pr(>|t|)
(Intercept)                344.849090   1.721159 200.359   <2e-16 ***
ald$last_distractor_offset  -0.005175   0.003093  -1.673   0.0945 .
---
Signif. codes:  0 '***' 0.001 '**' 0.01 '*' 0.05 '.' 0.1 ' ' 1

Residual standard error: 81.27 on 2230 degrees of freedom
Multiple R-squared:  0.001253,       Adjusted R-squared:  0.0008056
F-statistic: 2.799 on 1 and 2230 DF,  p-value: 0.09448

>
```

```
In [41]: print 'Moving-bin mean & 95% confidence intervals wrt distance to target'
```

```
Image(filename='r_images/movingbin_latvsdist.png')
```

Moving-bin mean & 95% confidence intervals wrt distance to target

Out[41]:

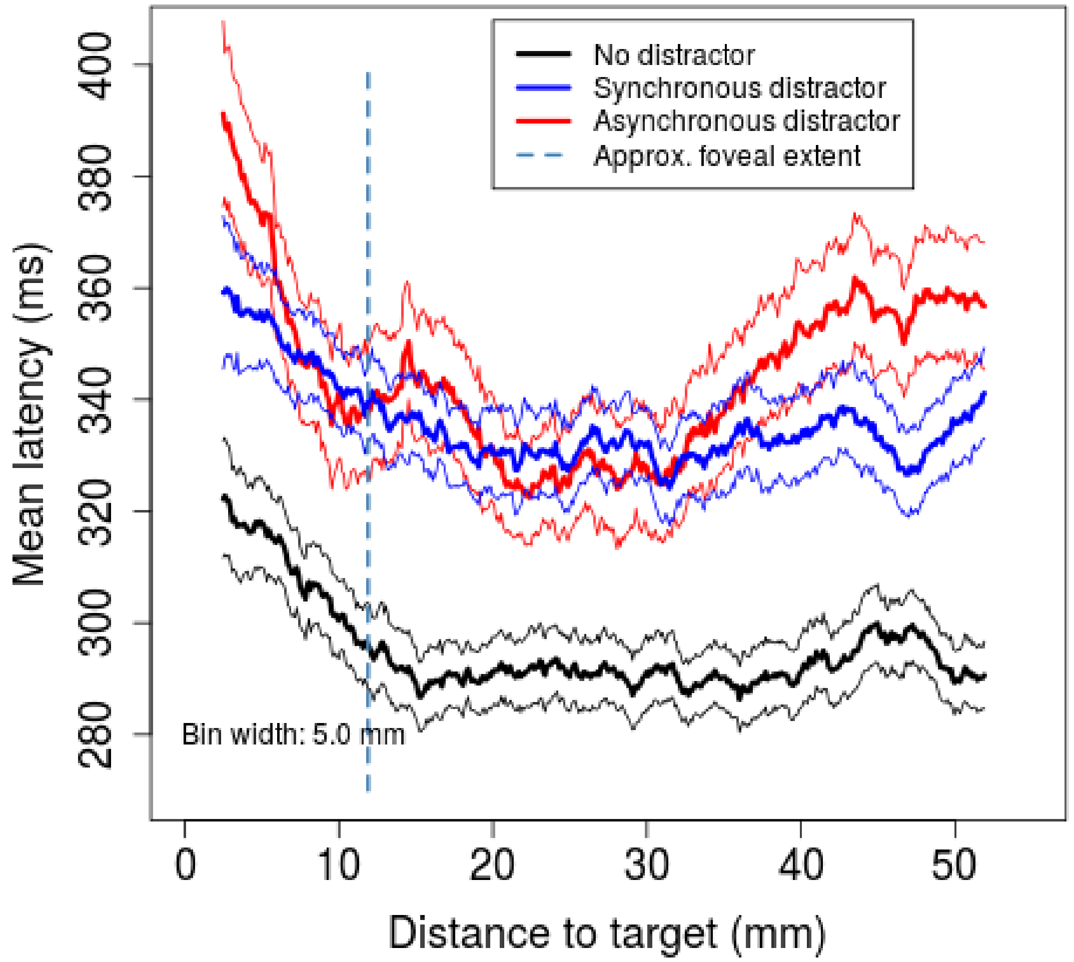


```
In [42]: print 'Directional version of the moving bin graph, above'
         Image(filename='r_images/movingbin_latvsdir.png')
```

Directional version of the moving bin graph, above

Out[42]:

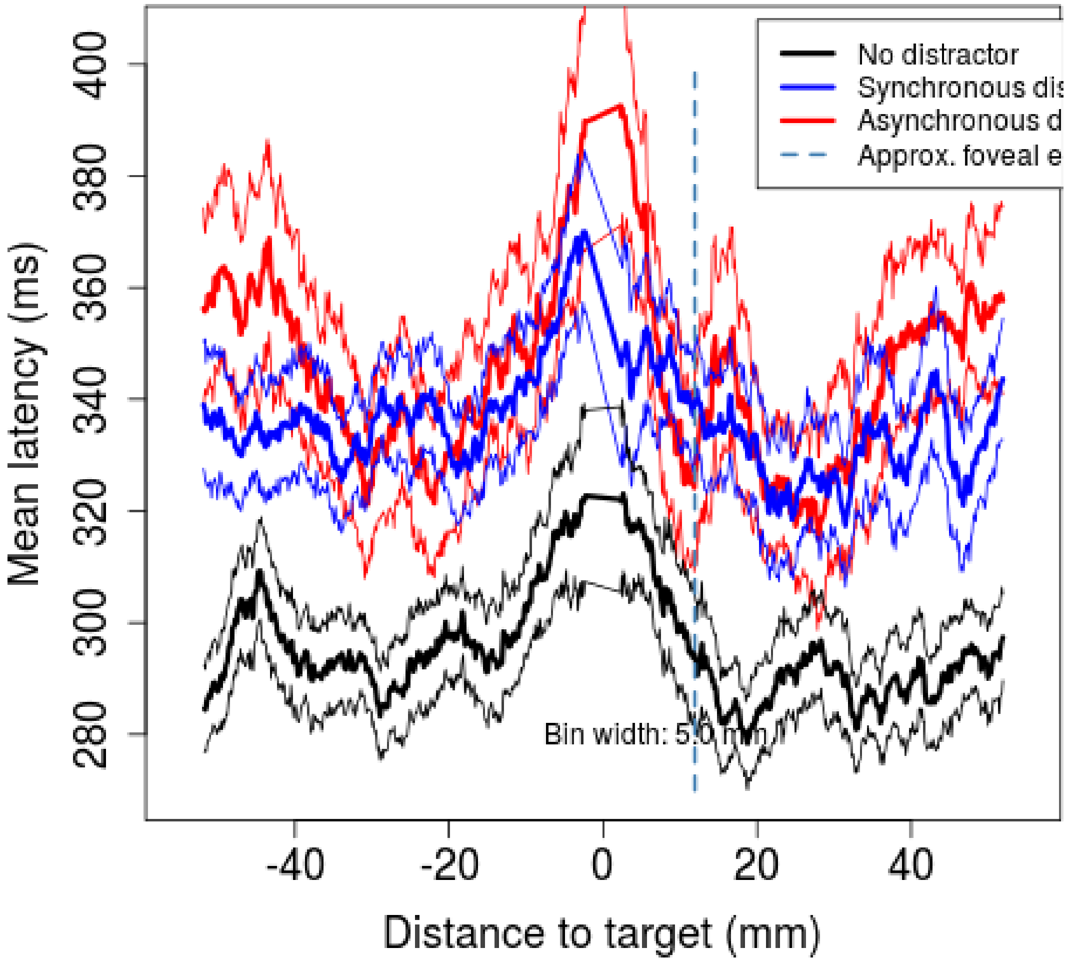


```
In [43]: print 'Moving-bin mean & 95% confidence intervals wrt distance to last dis
         Image(filename='r_images/movingbin_latvslastdist_async.png')
```

Moving-bin mean & 95% confidence intervals wrt distance to last distractor (green)

Out[43]:

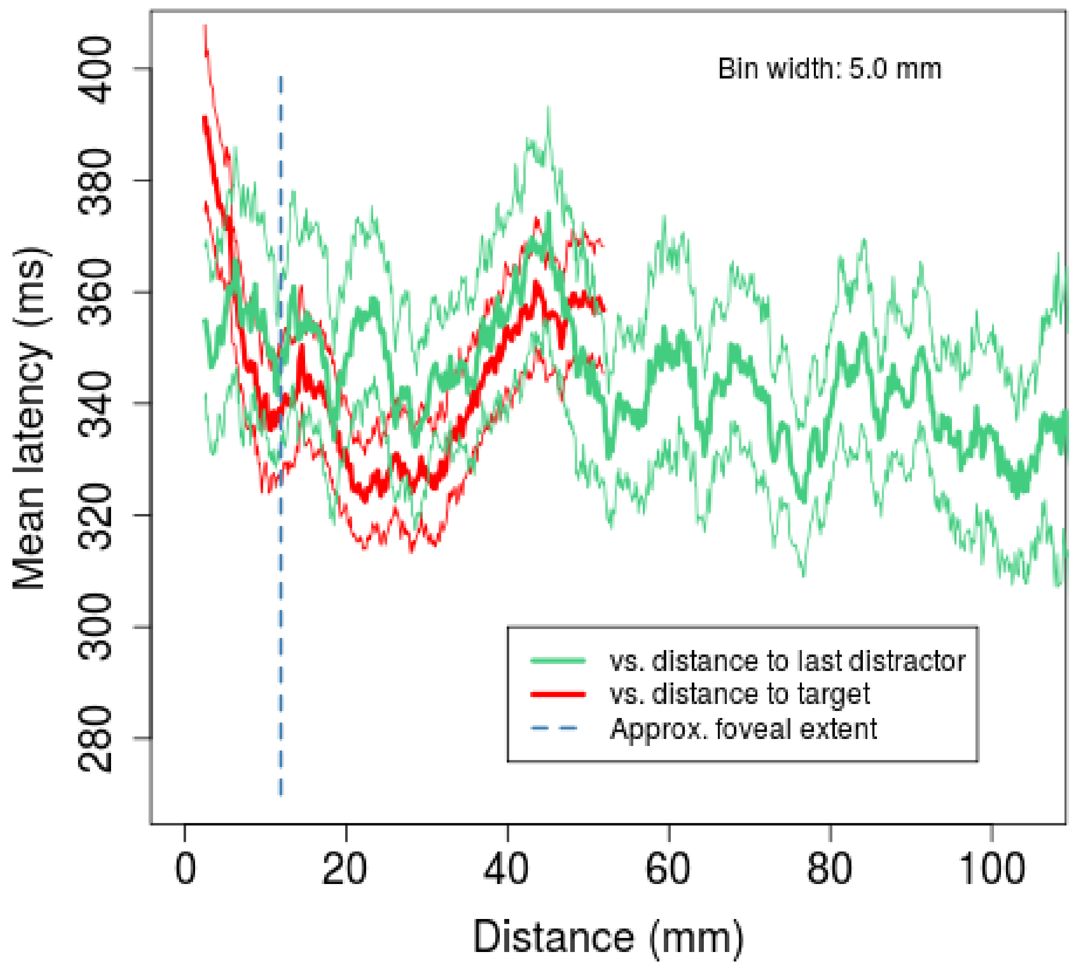

2 Alternative movement analysis

Mauro da Lio developed an alternative approach to finding movement latencies and errors from the raw position data; an alternative to the Octave script which creates fnames.mat. Here are scripts to load in the Summary.csv that is provided by his analysis, and also a code block to re-create an equivalent Summary.csv file from the Octave-generated data.

2.1 Reading data

To read data in for the alternative movement analysis, replace calls to readIndividuals() in the code blocks above with this alternative function readMauroIndividuals().

(I convert Summary.xls into Summary.csv before running this code.)

```
In [44]: # This function reads in data from Mauro's Summary.csv, producing the same
         # as is generated by readIndividuals()
         import csv
         def readMauroIndividuals ():
             with open('Mauro_analysis/Summary.csv', 'rb') as csvfile:

                 individuals = dict()
                 idnum_counter = 1
                 first = 1
                 dreader = csv.reader(csvfile, delimiter=',', quotechar='"')

                 for row in dreader:

                     # Skip first row (the header)
                     if first:
                         first = 0
                         continue

                     # Can access rows by column number:
                     #print row[3]
                     #print row
                     #print len(row)
                     #break

                     subj_id = row[1]
                     exp_name = row[0]

                     # condition index is for the no distractor/sync distractor/asy
                     condition_index = int(row[2])

                     # Need ONE individual object for each subj_id.
                     if subj_id not in individuals:
                         individuals[subj_id] = individual(subj_id)
                         individuals[subj_id].idnum = idnum_counter
                         individuals[subj_id].numSubsamples = 8
                         idnum_counter += 1

                     # No filename in Mauro's csv, just put name in each:
                     individuals[subj_id].filename_nd = exp_name
                     individuals[subj_id].filename_ad = exp_name
                     individuals[subj_id].filename_sd = exp_name

                     #individuals[subj_id].alldata_nd=np.ndarray(5)

                     # latencies for non-movement-error target events
                     ##tnoerr_latencies = fname[4] # R.latency_noerror_target - Use
                     # latencies for non-movement-error distractor events
                     ##dnoerr_latencies = fname[5]
```

```
# err_latencies = fname[6] # ALL error events, distractor and
# latencies for target movement error events
##terr_latencies = fname[7] # R.latency_error_target
# latencies for distractor movement error events
##derr_latencies = fname[8] # R.latency_error_distractor
if condition_index == 0:
    i=10 # starting col for the latencies in Mauro's spreadshe
    while i < len(row):
        if len(row[i]) == 0:
           break
        rowval = float(row[i])
        # New latency:
        newlat = np.array([i+1,rowval])
        if (rowval >= 0):
            # number, type (TARG=1 here), error, correct_move,
            newalldata = np.array([i+1, 1, 0, 1, rowval])
            if individuals[subj_id].nodist_latencies.size == (
                individuals[subj_id].nodist_latencies = newlat
            else:
                individuals[subj_id].nodist_latencies = np.vst
        else:
            newalldata = np.array([i+1, 1, 1, 0, rowval])
            if individuals[subj_id].nodist_err_latencies.size
                individuals[subj_id].nodist_err_latencies = -1
            else:
                individuals[subj_id].nodist_err_latencies = np

        if individuals[subj_id].alldata_nd.size == 0:
            individuals[subj_id].alldata_nd = newalldata
        else:
            individuals[subj_id].alldata_nd = np.vstack((indiv

        #print 'alldata',individuals[subj_id].alldata_nd
        i+=1
    # Compute ranks
    #print 'nodist latencies:',individuals[subj_id].nodist_lat
    #print 'nodist_err latencies:',individuals[subj_id].nodist
    if individuals[subj_id].nodist_latencies.size>0:
       individuals[subj_id].nodist_latencies_rank = stats.rank
    if individuals[subj_id].nodist_err_latencies.size>0:
        if individuals[subj_id].nodist_err_latencies.size==2:
            individuals[subj_id].nodist_err_latencies_rank = [
        else:
            individuals[subj_id].nodist_err_latencies_rank = s

    # nerrs code identical to that in readIndividuals
    nerrs = 0
    ntargets = 0
```

```
        for d in individuals[subj_id].alldata_nd:
            ntargets += 1 # "ndistractors" is really "ntargets" fo
            if d[2] > 0.0:
                nerrs += 1
        individuals[subj_id].n_errors_per_target_nd = (float(nerrs
        #print individuals[subj_id].n_errors_per_target_nd

    if condition_index == 1:
        i=10 # starting col for the latencies in Mauro's spreadshe
        while i < len(row):
            if len(row[i]) == 0:
               break
            rowval = float(row[i])
            # New latency:
            newlat = np.array([i+1,rowval])
            if (rowval >= 0):
                # number, type (TARG=1 here), error, correct_move,
                newalldata = np.array([i+1, 1, 0, 1, rowval])
                if individuals[subj_id].sync_latencies.size == 0:
                    individuals[subj_id].sync_latencies = newlat
                else:
                    individuals[subj_id].sync_latencies = np.vstac
            else:
                newalldata = np.array([i+1, 1, 1, 0, rowval])
                if individuals[subj_id].sync_err_latencies.size ==
                    individuals[subj_id].sync_err_latencies = -1*n
                else:
                    individuals[subj_id].sync_err_latencies = np.v

            if individuals[subj_id].alldata_sd.size == 0:
                individuals[subj_id].alldata_sd = newalldata
            else:
                individuals[subj_id].alldata_sd = np.vstack((indiv

            #print 'alldata',individuals[subj_id].alldata_sd
            i+=1
        #print 'sync latencies:',individuals[subj_id].sync_latenci
        #print 'sync_err latencies:',individuals[subj_id].sync_err
        # Compute ranks
        if individuals[subj_id].sync_latencies.size>0:
            individuals[subj_id].sync_latencies_rank = stats.rankd
        if individuals[subj_id].sync_err_latencies.size>0:
            if individuals[subj_id].sync_err_latencies.size==2:
                individuals[subj_id].sync_err_latencies_rank = [1]
            else:
                individuals[subj_id].sync_err_latencies_rank = sta

        # nerrs code identical to that in readIndividuals
```

```
            nerrs = 0
            ntargets = 0
            for d in individuals[subj_id].alldata_sd:
                ntargets += 1 # "ndistractors" is really "ntargets" fo
                if d[2] > 0.0:
                    nerrs += 1
            individuals[subj_id].n_errors_per_distractor_sync = (float
            #print individuals[subj_id].n_errors_per_distractor_sync

        if condition_index == 2:
            # FIXME: FINISH ME
            i=10 # starting col for the latencies in Mauro's spreadshe
            while i < len(row):
                if len(row[i]) == 0:
                   break
                rowval = float(row[i])
                # New latency:
                newlat = np.array([i+1,rowval])
                if (rowval >= 0):
                    # number, type (TARG=1 here), error, correct_move,
                    newalldata = np.array([i+1, 1, 0, 1, rowval])
                    if individuals[subj_id].async_latencies.size == 0:
                        individuals[subj_id].async_latencies = newlat
                    else:
                        individuals[subj_id].async_latencies = np.vsta
                else:
                    newalldata = np.array([i+1, 1, 1, 0, rowval])
                    if individuals[subj_id].async_err_latencies.size =
                        individuals[subj_id].async_err_latencies = -1*
                    else:
                        individuals[subj_id].async_err_latencies = np.

                if individuals[subj_id].alldata_ad.size == 0:
                    individuals[subj_id].alldata_ad = newalldata
                else:
                    individuals[subj_id].alldata_ad = np.vstack((indiv

                #print 'alldata',individuals[subj_id].alldata_ad
                i+=1
            #print 'async latencies:',individuals[subj_id].async_laten
            #print 'async_err latencies:',individuals[subj_id].async_e
            # Compute ranks
            if individuals[subj_id].async_latencies.size>0:
                individuals[subj_id].async_latencies_rank = stats.rank
            if individuals[subj_id].async_err_latencies.size>0:
                if individuals[subj_id].async_err_latencies.size==2:
                    individuals[subj_id].async_err_latencies_rank = [1
                else:
```

```
                    individuals[subj_id].async_err_latencies_rank = st

                # nerrs code identical to that in readIndividuals
                nerrs = 0
                ntargets = 0
                for d in individuals[subj_id].alldata_ad:
                    ntargets += 1 # "ndistractors" is really "ntargets" fo
                    if d[2] > 0.0:
                        nerrs += 1
                individuals[subj_id].n_errors_per_distractor_async = (floa
                #print individuals[subj_id].n_errors_per_distractor_async

        return individuals
```

2.2 Reproduce Summary Spreadsheet for comparison with Mauro's analysis

Mauro has Summary.xls, which lists the following columns:

Experimenter Subject Distractor type RT (M) RT (SD) N correct RT incorrect (M) RT incorrect (SD) N incorrect Error rate Delays (negative = incorrect)

The following code creates an equivalent Summary.csv from the data in fnames.dat.

```
In [45]: from __future__ import division
         import numpy as np
         from statsmodels.stats.multicomp import MultiComparison

         maxCount = 100 # 1 for debug or 100 for all
         count = 1
         csvdata = ''

         individuals = readIndividuals()

         for i in individuals:
             ind = individuals[i]

             if count == 1:
                 csvdata = ind.csvheader()

             # Remove outliers in standard way
             ind.excludeOutliers()

             csvdata += ind.csvlineset()

             # For debugging, break after maxCount
             if count >= maxCount:
                 break
             count += 1

         print 'Writing Summary.csv...'
```

```
        f = open('Summary.csv', 'w')
        f.write(csvdata)
        f.close()
```

```
Writing Summary.csv...
```

2.3 Alternative method analysis

This applies an analysis of movement latencies to the alternative latency extraction method.

```
In [46]: from subprocess import CalledProcessError, check_output
         try:
             out = check_output(["R", "-q --file=Analyse_AltExtrMethod.r"])
         except CalledProcessError as err:
             print 'Error for:',err.cmd,'with error code:',err.returncode
             print "\nOutput:\n\n",err.output
         else:
             for line in out.splitlines():
                 print line
```

```
> ###
> ### Analysis on Mauro's alternative method for latencies.
> ###
>
> set.seed(1974201701)
>
> ## Use functions from this script:
> source('Bootstrap_all_twosamples.r')
>
> ## Read Mauro's data in
> d <- read.csv('Mauro_analysis/Summary_reduced.csv')
> f1 <- function (type) {
+     ## Get all rows for distractor.type == 0
+     dd<-d[which(d$Distractor.type == type), ]
+     ## remove Subject and Distractor.type columns]
+     dd$Subject <- NULL
+     dd$Distractor.type <- NULL
+     ## Matrix to vector (don't care which individual is which):
+     dd <- unlist(dd)
+     ## Remove nd fields:
+     dd <- na.omit(dd)
+     ## Remove negative fields and convert to ms:
+     dd<-dd[dd>0]*1000
+ }
>
> ND<-f1(0)
> SD<-f1(1)
> AD<-f1(2)
```

```
>
> print (sprintf("ND mean: %f, Std Dev: %f", mean(ND), sd(ND)))
[1] "ND mean: 292.624346, Std Dev: 52.853583"
> print (sprintf("SD mean: %f, Std Dev: %f", mean(SD), sd(SD)))
[1] "SD mean: 329.931663, Std Dev: 55.533088"
> print (sprintf("AD mean: %f, Std Dev: %f", mean(AD), sd(AD)))
[1] "AD mean: 341.576577, Std Dev: 71.290504"
>
> print("------------------------------------------")
[1] "------------------------------------------"
> print("Bootstrap analysis of difference of means")
[1] "Bootstrap analysis of difference of means"
> ndsd <- b.diffste(SD, ND, 1024)
> print (sprintf("SD ND difference is %f, standard error estimate: %f", ndsd$meandi
[1] "SD ND difference is 37.307317, standard error estimate: 1.996550"
> ndad <- b.diffste(AD, ND, 1024)
> print (sprintf("AD ND difference is %f, standard error estimate: %f", ndad$meandi
[1] "AD ND difference is 48.952231, standard error estimate: 2.342485"
> sdad <- b.diffste(AD, SD, 1024)
> print (sprintf("AD SD difference is %f, standard error estimate: %f", sdad$meandi
[1] "AD SD difference is 11.644914, standard error estimate: 2.453404"
>
> print("------------------------------------------")
[1] "------------------------------------------"
> print ("Studentized bootstrapped hypothesis test (Algo 16.2)")
[1] "Studentized bootstrapped hypothesis test (Algo 16.2)"
> # ndsd does better than 1e-6 (set B to 1000000)
> ndsd_tst <- b.studentized_ttest(SD, ND, 1000)
> b.showsiglev (ndsd_tst, "SD vs ND")
[1] "Achieved significance level for SD vs ND < 0.001000"
> ndsd_tst <- b.studentized_ttest(AD, ND, 1000)
> b.showsiglev (ndsd_tst, "AD vs ND")
[1] "Achieved significance level for AD vs ND < 0.001000"
> ndsd_tst <- b.studentized_ttest(AD, SD, 1000)
> b.showsiglev (ndsd_tst, "AD vs SD")
[1] "Achieved significance level for AD vs SD < 0.001000"
>
> print("------------------------------------------")
[1] "------------------------------------------"
>
> # Now make a density graph, like Fig 6 in original submission.
> print ('raw distributions')
[1] "raw distributions"
>
> doplot <- function () {
+
+     par(mar=c(5,5,3,5))
+     par(oma=c(0,0,0,0))
```

```
+
+     lat.NDcol <- "black"
+     lat.SDcol <- "steelblue"
+     lat.ADcol <- "red"
+
+     lat.raw.xmin <- 200
+     lat.raw.xmax <- 800
+     lat.raw.ymin <- 0
+     lat.raw.ymax <- 0.012
+     lat.raw.legx <- 365
+     lat.raw.legy <- 0.012
+                                         # ND is blue
+     df <- density(ND, n=1024)
+
+     plot(df, lwd=3, col=lat.NDcol, ylim=c(lat.raw.ymin,lat.raw.ymax), xlim=c(lat.
+          cex.lab=1.8, cex.axis=1.8, cex.main=1.8, cex.sub=1.8,
+          main="Latency measurement distributions (alt method)", xlab="Latency (ms
+     abline(v=mean(ND), lty=2, lwd=2, col=lat.NDcol)
+
+     df1 <- density(SD, n=1024)
+     lines(df1, lwd=3, col=lat.SDcol, xlim=c(lat.raw.xmin:lat.raw.xmax))
+     abline(v=mean(SD), lty=2, lwd=2, col=lat.SDcol)
+
+     df2 <- density(AD, n=1024)
+     lines(df2, lwd=3, col=lat.ADcol, xlim=c(lat.raw.xmin:lat.raw.xmax))
+     abline(v=mean(AD), lty=2, lwd=2, col=lat.ADcol)
+
+     legend (lat.raw.legx, lat.raw.legy, bg="white",
+             c(sprintf("No distractor (mean %.1f ms)", mean(ND)),
+               sprintf("Synchronous (%.1f ms)", mean(SD)),
+               sprintf("Asynchronous (%.1f ms)", mean(AD))),
+             lty=c(1,1,1),
+             lwd=c(3,3,3),
+             cex=1.4,
+             col=c(lat.NDcol,lat.SDcol,lat.ADcol))
+ }
>
> setEPS()
> postscript(sprintf('../paper/figures/data_density_altmethod.eps'),width=8,height=
> doplot()
> dev.off()
null device
          1
>
> png(sprintf('../paper/figures/data_density_altmethod.png'),width=800, height=500,
> doplot()
> dev.off()
null device
```

```
            1
>
```

```
In [47]: print 'Alternative method, data density plot'
         Image(filename='../paper/figures/data_density_altmethod.png')
```

```
Alternative method, data density plot
```

```
Out[47]:
```

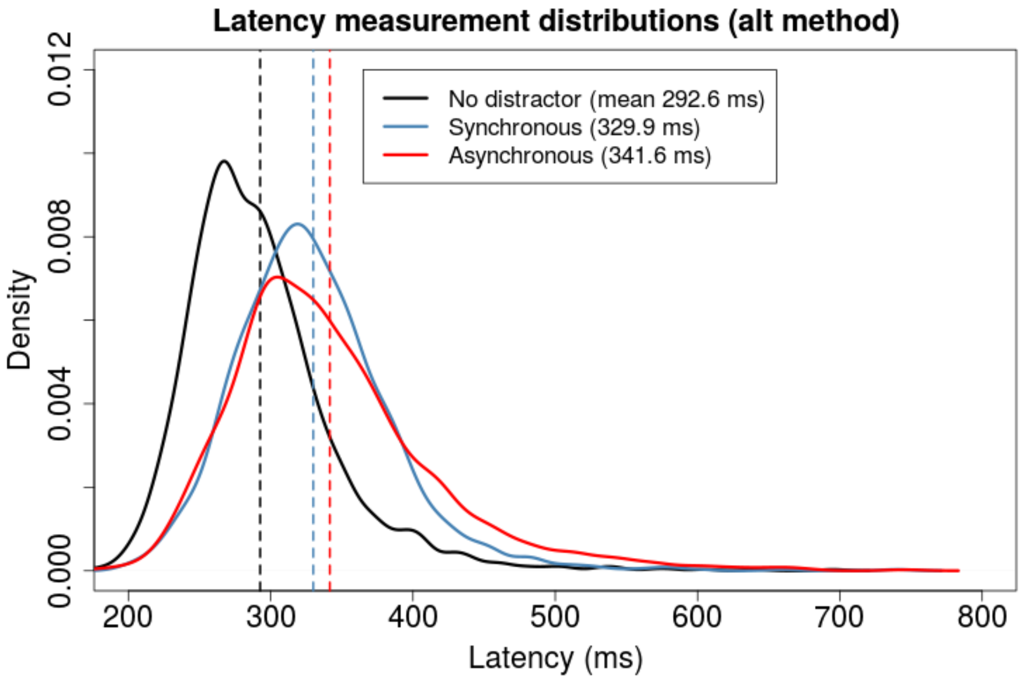


```
In [ ]:
```
